# The microglia-derived protein Sema4ab attenuates regenerative neurogenesis after spinal cord injury in zebrafish

**DOI:** 10.1371/journal.pbio.3003865

**Published:** 2026-06-18

**Authors:** Alberto Docampo-Seara, Mehmet Ilyas Cosacak, Kim Heilemann, Friederike Kessel, Ana-Maria Oprişoreanu, Markus Westphal, Özge Çark, Daniela Zöller, Josi Arnold, Anja Bretschneider, Alisa Hnatiuk, Nikolay Ninov, Catherina G. Becker, Thomas Becker

**Affiliations:** 1 Center for Regenerative Therapies Dresden at the TU Dresden, Dresden, Germany; 2 Institute for Neuroscience and Cardiovascular Research, Chancellor’s Building, University of Edinburgh, Edinburgh, United Kingdom; 3 Cluster of Excellence Physics of Life, TU Dresden, Dresden, Germany; University of Notre Dame, Center for Stem Cells and Regenerative Medicine, UNITED STATES OF AMERICA

## Abstract

Zebrafish, in contrast to mammals, regenerate neurons after spinal cord injury, but little is known about the control mechanisms of this process. Here we use scRNA-seq and *in vivo* experiments to show that *sema4ab*, mainly expressed by lesion-reactive microglia, attenuates regenerative neurogenesis by changing the complex lesion environment. After spinal injury, disruption of *sema4ab* doubles the number of newly generated progenitor cells and neurons but attenuates axon regrowth and recovery of swimming function. Disruption of the *plxnb1a/b* receptors, selectively expressed by neural progenitor cells, increases regenerative neurogenesis. In addition, disruption of *sema4ab* alters activation state and cytokine expression of microglia, such that fibroblasts increase expression of the cytokine *tgfb3*, which strongly promotes regenerative neurogenesis. Hence, we propose that *sema4ab* expression in microglia attenuates regenerative neurogenesis in multiple ways, likely directly through *plxnb1a/b* receptors and indirectly, by controlling the inflammatory milieu and *tgfb3* levels*.* Targeting Sema4A-dependent signaling in non-regenerating vertebrates may be a future strategy to improve regenerative outcomes.

## Abstract 2

Zebrafish can regenerate neurons after spinal cord injury, but the mechanisms influencing this ability remain unknown. This study shows that Sema4ab, a protein expressed in lesion-reactive microglia, attenuates regenerative neurogenesis by directly regulating neural progenitors and altering cytokine signaling in the niche.

## Introduction

Mammals, including humans, fail to regenerate neurons lost to protracted secondary cell death around a spinal injury site [1,2]. Spinal progenitor cells can generate new neurons in a conducive environment and can even be re-programmed *in vivo* to produce oligodendrocytes, but so far not neurons [3–5]. Reasons underlying lack of regenerative neurogenesis after spinal cord injuries are currently unknown, but this failure has been partially attributed to a complex and largely inhibitory environment, which consists of immune cells, such as invading blood-derived macrophages (BDMs) and tissue-resident microglia, astrocytes, oligodendrocytes, and fibroblasts. Interactions of these cell types result in an inhibitory environment for regenerative neurogenesis in which prolonged inflammation, as well as fibrotic and astrocytic scarring predominates, which supresses regenerative neurogenesis and axonal regrowth [6–10].

In contrast to mammals, zebrafish functionally regenerate their spinal cord at the adult and larval stage, restoring axonal connectivity and swimming function [11–15]. This includes injury-induced generation of new neurons from resident progenitors [14,16–19]. We use the term ependymo-radial glial cells (ERGs) for these progenitors, to illustrate their combination of ependymal and astroglial features [20]. Importantly, regenerative neurogenesis does not lead to over-proliferation and tumor formation, which may be encountered when transplanting neural progenitor cells to a spinal lesion [21], or exhaustion of the progenitor pool, for example, observed in a stroke model in mice [22]. Moreover, functionality of neural networks can be perturbed by overproduction of new neurons, such as in the hippocampus of mice after stroke-induced neurogenesis [23]. Hence, production of new neurons must be precisely regulated in zebrafish.

Successful spinal cord regeneration requires a precise balance between multiple repair processes, including neurogenesis, axon regrowth, inflammatory resolution, and tissue remodeling. A relatively large number of signaling molecules from various cellular sources have been discovered that promote regenerative neurogenesis and axon growth in the zebrafish model [13,24–30]. However, only a few negative regulators of regenerative neurogenesis are known in zebrafish [31].

Microglia are in a key position to provide control mechanisms during regeneration. After a spinal cord injury, microglial cells proliferate and invade the lesion site [32]. In humans, microglia are known to promote developmental neurogenesis [33], but activated microglia is mostly detrimental to it [34]. In regeneration, microglia likely influence the signaling milieu in the entire injury environment [35], which affects the state of other regeneration-relevant cell types that also influence regenerative neurogenesis. For example, ERGs and astrocyte-like cells produce a range of regeneration-promoting growth factors in central nervous system (CNS) injuries in zebrafish [13,26,27]. Additionally, microglia may influence fibroblasts in the spinal lesion site, which in turn promote regeneration by producing favorable extracellular matrix components [30,36,37].

To understand how microglia influence the signaling landscape in the injury site and regenerative neurogenesis in zebrafish, we analyzed the role of the conserved signaling molecule Semaphorin 4A (Sema4A), which is mainly expressed by microglia and can modulate their activation state [38,39]. Sema4A has been shown to promote a pro-fibrotic phenotype in fibroblasts *in vitro*, suggesting possible effects on fibroblasts [40]. Knock out of the principal Sema4A receptors Plexin B1 and B2 impairs neurogenesis in the developing mouse brain [41], suggesting microglia to progenitor cell communication via Sema4A.

Here, we find that disrupting a zebrafish orthologue of *Sema4A*, *semaphorin 4ab (sema4ab)*, increases regenerative neurogenesis, but impairs axon regrowth and functional recovery. *Sema4ab* potentially acts on ERGs via the receptor paralogs *plexin b1a* (*plxnb1a*) and *plexin b1b (plxnb1b*) and indirectly by altering the cytokine milieu, which leads to reduced production of the pro-neurogenic factor *tgfb3* in fibroblasts. Hence, we propose that *sema4ab+* microglia regulate regenerative neurogenesis in the regeneration-permissive cellular environment and promote recovery of the zebrafish spinal cord after lesion.

## Results

### *sema4ab*-dependent microglia signaling indicated by scRNA-seq

After a complete spinal lesion at 3 days post-fertilization (dpf), lesion-induced neurogenesis peaks at 24 hours post-lesion (hpl) in larval zebrafish. By 48 hpl, axons have regrown over the lesion site and swimming function is largely recovered [14,30], such that major events in spinal cord regeneration occur within 48 hours in this system.

To find potential signals from microglia that act on neural progenitors after injury, we interrogated previous scRNA-seq data sets of enriched immune cells and of enriched neural progenitors at 24 hpl in larval zebrafish that had received a spinal cord lesion at 3 dpf [42]. In the immune cell data set, immune cells were enriched by FACS sorting for GFP expression under the *mpeg1.1* regulatory sequences (S1A Fig). This strongly enriched BDMs and microglia, but the sample also contained neutrophils and other cell types, possibly because *mpeg1.1* was also expressed in other cell types [42–44]. We observed a sharp increase in microglia, identified by expression of *apoeb* [45], at 24 hpl (S1B and S1C Fig). We found that the signaling molecule *sema4ab* was expressed by microglia and BDMs (S1D Fig). Interestingly, of all the semaphorins detectably expressed in this dataset, *sema4ab* was markedly more strongly expressed and also upregulated after lesion, compared to all other family members, including the paralog *sema4aa* (S1E Fig).

We also searched for potential ligand-receptor interactions with progenitor cells using a data set enriched for expression of *her4.1*, a marker of ERGs, which contain spinal progenitor cells (S1F and S1G Fig). We found enriched expression of the potential *sema4ab* receptor paralogs *plxnb1a* and *plxnb1b* [39,46] in ERGs (S1H–S1J Fig). While this indicates the potential for direct ligand-receptor interactions between these two cell types, cell interactions between different types of neural and non-neural cells in the injury site are complex, and altered signaling from microglia is likely to change the entire lesion environment.

**Fig 1:**
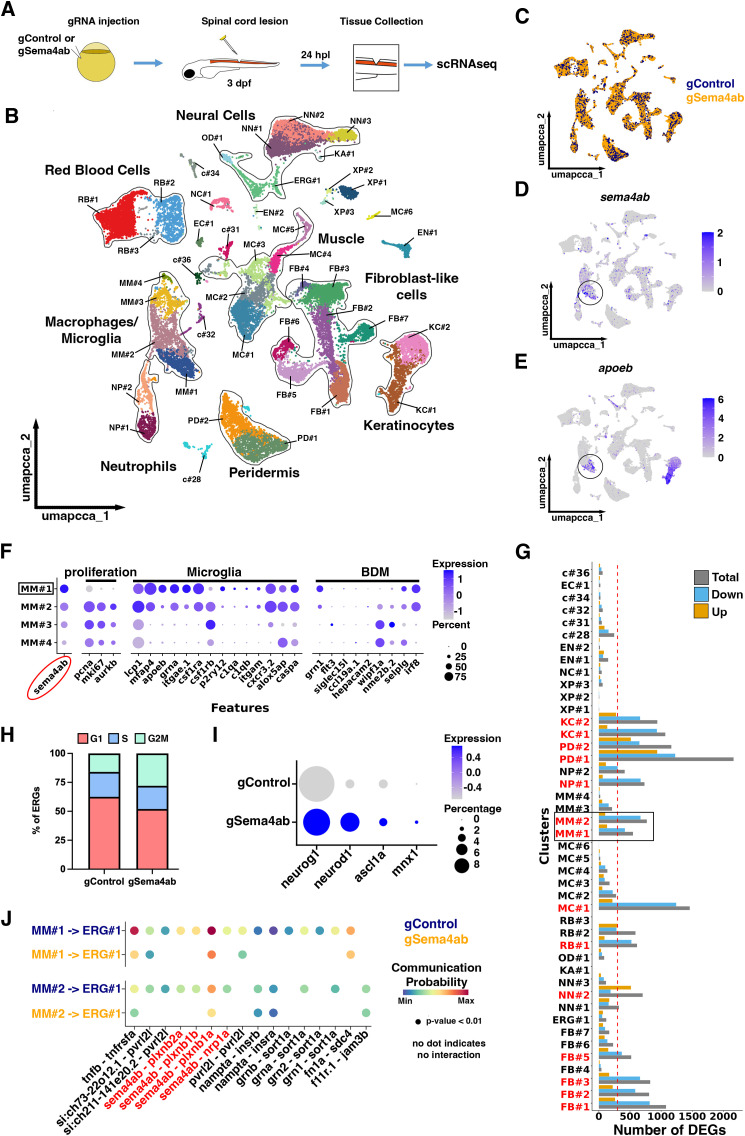
scRNA-seq indicates changes gene expression after *sema4ab* ablation and predicts changes in regenerative neurogenesis. **(A)** A schematic of the experimental design for scRNA-seq in somatic mutants is shown. **(B)** Cluster analysis identifies 44 cell clusters that represent the main expected cell types in the injury site at 24 hpl. **(C)** A UMAP shows no major changes in cell type representation resulting from *sema4ab* disruption (gControl in blue, gSema4ab in yellow). **(D,E)** Feature plots for *sema4ab* (D) and *apoeb* (microglia; E) show overlap (black circles). **(F)** A dot plot of the four BDMs/microglia clusters showing that *sema4ab*-enriched clusters align with the expression of microglia marker genes. **(G)** A graph indicating differential expression of genes (DEG) between samples is shown. Cell clusters with major changes (>300 DEGs) are indicated in red. Microglia clusters MM#1 and MM#2 are boxed. **(H)** Graph shows the proportions of ERGs in each cell cycle phase in control gRNA-injected larvae and *sema4ab* gRNA-injected larvae. Note that the proportion of cells in M/G2 phase is increased in *sema4ab* somatic mutants (gControl: 16% vs gSema4ab: 28%). **(I)** Dot plot showing an increase in differentiation marker gene expression in ERG progenitors when *sema4ab* is disrupted. **(J)** Dot plot illustrating the communication probabilities of all ligand-receptor interactions identified between MM#1 and ERGs, as well as between MM#2 and ERGs. The absence of dots indicates that no interaction was detected. For a key to cluster abbreviations in B and G, see S5 Table.

To get an overview of most, if not all cells in the injury site, and to assess the effect of *sema4ab* ablation simultaneously on all of these cell types, we generated scRNA-seq profiles of the entire trunk tissue flanking the spinal injury site with and without disruption of *sema4ab*. To disrupt *sema4ab,* we injected larvae with highly-active CrRNAs (haCRs) that lead to mutagenesis of the targeted site in nearly all somatic cells, allowing phenotype analysis already in haCR-injected somatic mutants for *sema4ab* [42,47–50]. We generated scRNA-seq profiles of lesion site tissue from 50 control gRNA-injected and 50 *sema4ab* haCR*-*injected larvae at 24 hpl, the peak of regenerative neurogenesis [14] (Fig 1A).

Our scRNA-seq experiments yielded 11,788 cells for the control gRNA-injected sample (gControl), 11,621 cells for sample 1 (gSema4abR1), and 11,330 cells for sample 2 (gSema4abR2) of two technical repeats of *sema4ab* haCR*-*injected larvae, after quality control (S1 and S2 Tables). The haCR manipulation of *sema4ab* was highly penetrant, as no intact reads of the targeted site could be retrieved, contrary to controls (151 correct reads in controls; 4 and 6 reads, none of which were correct, in *sema4ab* haCR*-*injected larvae; S3 and S4 Tables). Moreover, principal component analysis indicated that the two technical repeats of the *sema4ab* haCR-treated condition were closely related and more distant from the control sample, indicating reproducibility of the approach (S2A Fig).

Unsupervised clustering and annotation revealed 44 distinct cell clusters that fell into 15 major cell categories (Fig 1B and S5 Table). Fibroblast-like cells constituted the majority of all cells (21.13%), consisting of 7 clusters, followed by neural cells (14.86%), consisting of ERGs, oligodendrocytes and 4 neuronal clusters, red blood cells (13.54%), consisting of three clusters, muscle cells (13.54%), consisting of 5 clusters, and immune cells (11.63%), consisting of 4 BDM/microglia and 2 neutrophil clusters. These clusters were represented in both conditions, as observed in a UMAP projection showing intermingling between cells from control and *sema4ab*-disrupted samples (Fig 1C). Hence, all major cell types expected from histology were represented in the samples, allowing for comprehensive analyses of cell interactions in the lesioned larval zebrafish spinal cord.

High and selective expression of *sema4ab* was found in a cluster of macrophages/microglia and mainly in the *apoeb+* cluster MM#1 (Fig 1D and 1E). Using the microglia marker *apoeb* and a marker set for macrophages/microglia from adult zebrafish brain after traumatic brain injury [51], we found that MM#1 and MM#2 expressed higher levels of microglial marker genes in this set, compared to MM#3 and MM#4 (Fig 1F). Based on the majority of expressed markers, MM#1 and MM#2 were classified as microglia and MM#3 and MM#4 as BDMs. Of note, most of the markers co-segregated with microglia and BDM identities. However, some of the markers did not fully agree with the assigned cell identities. This may be due to regional heterogeneity within the CNS and/or differences across zebrafish developmental stages that affect marker expression by BDMs and microglia. Population MM#2, in contrast to population MM#1, expressed proliferation markers, but otherwise marker expression was very similar between the two populations (Fig 1F). Therefore, MM#2 likely is a proliferative sub-group of microglia and together with population MM#1 represent microglia. Hence, microglia is likely the main cell type expressing *sema4ab* in the injury site.

To analyze alterations in the scRNA-seq profiles resulting from *sema4ab* disruption, we first compared cell numbers between samples. This indicated no major changes in cell type representation resulting from *sema4ab* disruption with the possible exception of peridermis cluster 2 (PD#2), which showed a ~50% reduction in cell number (S2B Fig).

Next, we analyzed gene expression changes per cluster. Considerable changes in gene expression (>300 differentially expressed genes) were present in microglia (MM#1, MM#2), compared to BDM-like clusters (MM#3, MM#4), which correlates with the expression levels of *sema4ab* in these cell cluster. Fibroblasts (FB#1, FB#2, FB#3, FB#5), another regeneration-relevant cell population, also showed high levels of gene expression changes, together with neutrophils (NP#1), neurons (NN#2), muscle (MC#1), peridermal cells (PD#1, PD#2), keratinocytes (KC#1, KC#2) and red blood cells (RB#1) (Fig 1G). Interestingly, most clusters showed reduced gene expression in the absence of *sema4ab* function, with the exception of the neuronal cluster NN#2 that showed increased gene expression. This indicates changes in gene expression across many cell types when *sema4ab* is disrupted.

To determine whether such changes may have influenced regenerative neurogenesis, we analyzed changes in proliferation and in gene expression related to neuronal differentiation in ERGs. Using the “Cell-Cycling Scoring” analysis code in Seurat [52] we calculated the proportion of ERGs in different cell cycle phases. We found a substantial increase in ERGs in G2/M phase in sema4ab-injected larvae (gControl: 16%, gSema4ab: 28%) (Fig 1H). Additionally, a comparison of neuronal differentiation markers (*neurog1*, *neurod1*, *ascl1a*) and motor neuron cell fate marker *mnx1* showed increased expression of these genes in ERGs in the absence of *sema4ab* function (Fig 1I). This is consistent with enhanced progenitor proliferation and neurogenesis.

To determine potential direct signaling from microglia to ERGs, we analyzed altered signal/receptor interactions between microglia and ERGs using CellChat, which evaluates cell-cell interactions based on known ligand-receptor interactions in scRNA-seq data [53]. That indicated reduced interactions of *sema4ab* with *plxnb1a*, *plxnb1b*, and *plxnb2a* (Fig 1J). Hence, scRNA-seq predicts microglia to directly signal to ERGs using Sema4ab to Plexin receptors.

**Fig 2:**
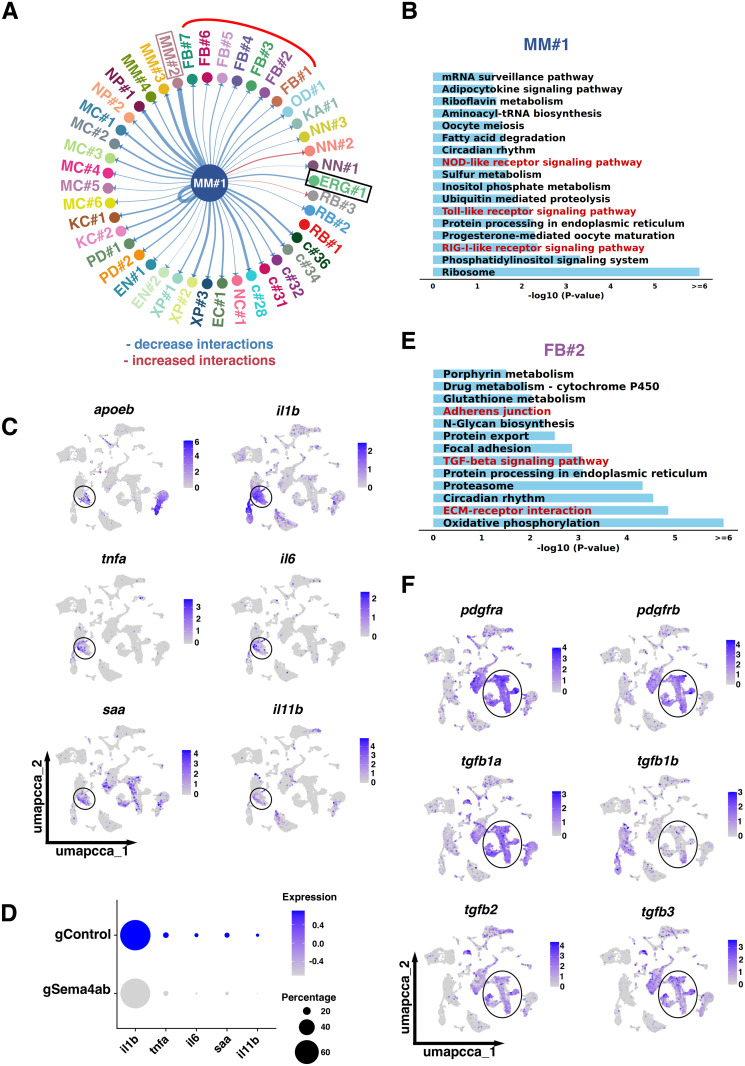
scRNA-seq indicates changes in cell-cell interactions between microglia and other cell types after *sema4ab* ablation. **(A)** CellChat analysis for MM#1 shows interactions with all cell types and a general decrease in these interactions (blue lines) when *sema4ab* is disrupted. Interactions of MM#1 with ERGs (black box), fibroblasts (red curved line) and MM#2 (pink box) are highlighted. **(B)** KEGG analysis of the main cluster that expresses *sema4ab* (MM#1) shows changes in immune activation pathways (highlighted in red). **(C)** Feature plots show enrichment of pro-inflammatory cytokines and inflammatory markers (*il1b, tnfa, il6, saa, il11b)* in microglia (*apoeb*, circle). **(D)** Dot plot showing reduction of expression of pro-inflammatory cytokines and inflammatory markers after *sema4ab* ablation. **(E)** KEGG analysis of the signaling pathways most affected by *sema4ab* disruption in fibroblast-like cell cluster FB#2 is shown. *tgfb* signaling and ECM interaction-related terms are highlighted in red. **(F)** Feature plots showing enrichment of anti-inflammatory cytokines (*tgfb1a, tgfb1b, tgfb2, tgfb3)* in fibroblasts (*pdgfra, pdgfrb;* circled).

To get a comprehensive view of how cell types could be affected by changes in microglia, we used CellChat to analyze the outgoing interactions of microglia cluster MM#1. This indicated potentially altered signal/receptor interactions with all identified cell clusters, including ERGs and fibroblasts, but mostly with themselves and their proliferative version MM#2. Most of these signal/ligand interactions were reduced when *sema4ab* was disrupted (Fig 2A).

To analyze the type of gene expression changes in microglia in the absence of *sema4ab* function, we used GOstats for KEGG analysis [54] on microglia cluster MM#1. This indicated changes in activation pathways, namely RIG-I-like receptor signaling [55], Toll-like receptor signaling pathways [56] and NOD-like receptor signaling [57] (Fig 2B). Most of the indicator genes for these GO terms were under-represented in the sample in which *sema4ab* was disrupted compared to the control sample (20 of 21 genes for RIG-I-pathway, 29 of 32 for Toll-like receptor pathway; 16 of 17 genes for NOD-like receptor signaling; S1 Data), suggesting reduced activation of microglia in the absence of *sema4ab*. Analysis of the top down-regulated genes in MM#1 confirmed this result (S6 Table). For example, one of the most underrepresented genes after *sema4ab* disruption, *lyz* (5.91-fold down), is related to a pro-inflammatory state [58,59]. Similarly, *grn1* (6.87-fold down), is linked to a pro-inflammatory state and cytokine production [60].

Based on this altered activation state of microglia, we analyzed expression levels in the scRNA-seq data of common pro-inflammatory cytokines in microglia (MM#1) and indeed found reduced expression for *il1b*, *tnfa*, *il6*, *saa*, and *il11b* (Fig 2C and 2D)*.* Reduced activation and pro-inflammatory cytokine production of microglia likely influences different cell types in the injury site.

Of note, fibroblasts have pivotal functions in successful spinal cord regeneration in zebrafish [61], and all 7 clusters of fibroblasts showed reduced incoming signals from microglia, when *sema4ab* was disrupted. Affected signal/receptor interactions included cytokine signaling (*e.g., tnfb, tgfb1b)*, among other pathways (S2C Fig). In addition, KEGG analysis for the three fibroblast populations that showed highest levels of differentially expressed genes after *sema4ab* disruption (FB#1, FB#2, FB#3) indicated ECM-receptor interactions, TGF-beta signaling (enriched in fibroblasts), and adherens junction pathways as changed (shown for FB#2 in Fig 2E). Interestingly, TGF-beta signaling genes *tgfb1a, tgfb2,* and *tgfb3,* but not *tgfb1b,* are enriched in fibroblasts, compared to BDM/microglia (Fig 2F) [62]. This data suggests that signaling and ECM-related functions were changed in fibroblasts when *sema4ab* was disrupted. Hence, scRNA-seq indicates multiple simultaneous signaling events of *sema4ab+* microglia with other non-neural lesion cells, such as fibroblasts, that likely condition the lesion environment. Overall, scRNA-seq suggests a scenario, in which *sema4ab* signaling from microglial cells to spinal progenitors, as well as via other cell types, attenuates neurogenesis.

### Expression of *sema4ab* and *plexinb1 receptors* *in vivo*

As a first step to verify scRNA-seq results *in vivo*, we determined gene expression patterns of *sema4ab* and *plxnb1a/b* in immune cells and neural progenitors, respectively. We used whole-mounted larvae for multiplexed hybridization chain reaction fluorescence *in situ* hybridization (HCR-FISH) in combination with detection of cell type-specific transgenic markers.

**Fig 3:**
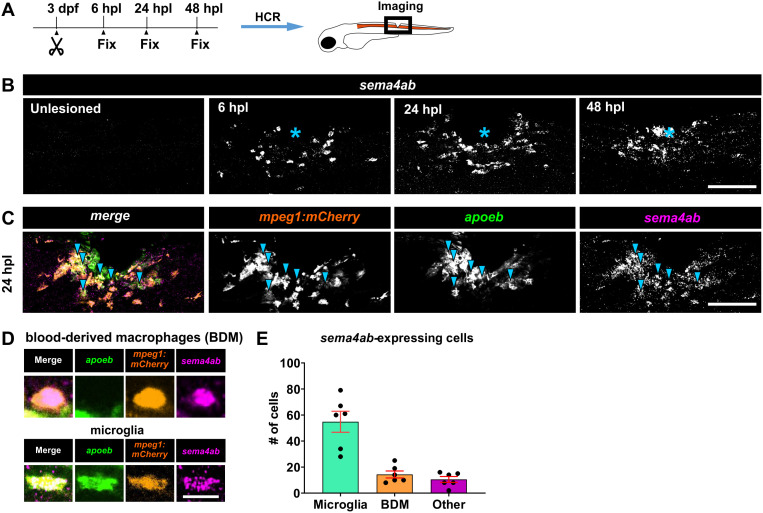
*Sema4ab* is expressed mainly in microglia in the spinal injury site. **(A)** Experimental timeline and schematic of a zebrafish larva indicating the lateral view of the spinal injury site shown throughout the article (unless specified differently) are shown. **(B)** A time-course analysis of HCR-FISH indicates increased expression of *sema4ab* during spinal cord repair. **(C)** Triple-multiplexed HCR-FISH at 24 hpl shows co-localization of *sema4ab, mpeg1:mCherry, and apoeb* at 24 hpl (blue arrowheads). **(D)** High magnifications of *apoeb/mpeg1:mCherry/sema4ab* labeling to differentiate blood-derived macrophages (BDMs; *apoeb−*) from microglia (*apoeb+*) for cell counting. **(E)** Quantifications at 24 hpl show that most *sema4ab-*expressing cells are microglia (*apoeb+/mpeg1:mCherry+*/*sema4ab+*). Error bars show SEM. Asterisks indicate the injury site. Scale bars: 100 µm **(B, C)**, 15 µm **(D)**. Data files for graphs available in S2 Data.

Microglia were identified in *mpeg1:mCherry* transgenic fish, in which mCherry is expressed under the *mpeg1.1* promoter [63], and by additional expression of *apoeb* (*mpeg1:mCherry*+/*apoeb*+). Putative BDMs were *mpeg1:mCherry*+/*apoeb*−. In uninjured three-day-old larvae, hardly any *sema4ab*+ cells were detectable, consistent with expression in injury-reactive immune cells. In contrast, at 6 hpl, numbers of *sema4ab*+ cells were strongly increased in the lesion site and remained high to at least 48 hpl (Fig 3A and 3B; see S3A Fig for method control). Co-labeling experiments at 24 hpl indicated that the majority of *sema4ab*+ cells were microglia (68.8%; *sema4ab+/mpeg1:mCherry+/apoeb+*). Eighteen percent were BDMs (*sema4ab*+/*mpeg1:mCherry*+/*apoeb−*), and 13.2% were unidentified (*sema4ab*+ only; Fig 3C–3E).

scRNA-seq analysis from Cavone and colleagues [42] showed that *sema4ab* was also expressed in neutrophils (see S1D Fig). To determine whether unidentified cells might be neutrophils we determined *sema4ab* expression in neutrophils using the *mpx:GFP* transgenic fish, in which GFP is expressed under the *mpx* promoter [64]. Co-labeling of *sema4ab* with *mpx:GFP* expressing neutrophils indicated that 16% of *sema4ab+* cells were neutrophils at 6 hpl and at 24 hpl (S3B Fig), indicating that the few *sema4ab+* cells not labeled by *mpeg1:mCherry* were likely mostly neutrophils. In summary, a spinal lesion induced the presence of *sema4ab+* cells that were mostly microglial cells.

**Fig 4:**
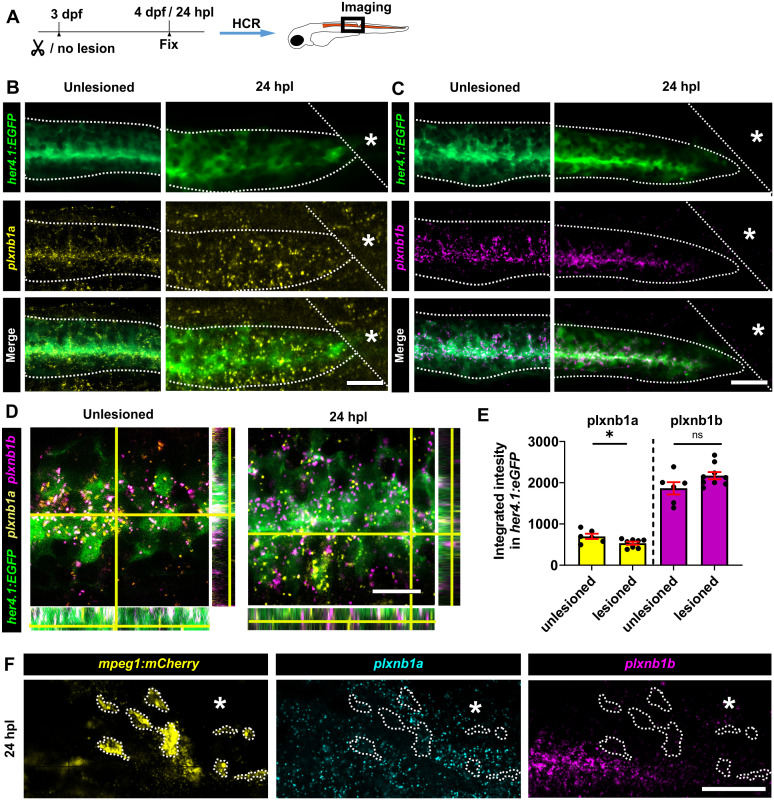
*plxnb1a* and *plxnb1b* are selectively expressed by spinal progenitors. **(A)** A schematic indicating the experimental design for HCR. Photomicrographs are oriented as in Fig 3. **(B-C)** HCR-FISH in the *her4.1:EGFP* line indicate expression of *plxnb1a* (B) and *plxnb1b* (C) in spinal progenitor cells (*her4.1:EGFP*+) before and after injury. Dotted lines indicate the spinal cord boundaries. **(D)** High magnifications with orthogonal views of co-localization of *plxnb1a* and *plxnb1b* in *her4.1:EGFP*+ cells before and after injury are shown. **(E)** Quantification showing a slight decrease of *plxnb1a* expression after injury, but no changes in *plxnb1b (plxnb1a:* unlesioned 699.4 a.u, lesioned 532.9 a.u, Unpaired *t* test: *p* = 0.0340; *plxnb1b:* unlesioned 1,865 a.u., lesioned 2,171 a.u, Unpaired *t* test: *p* = 0.0797). **(F)** HCR-FISH in the *mpeg1:mCherry* line indicates very low expression of *plxnb1a* and no detectable expression of *plxnb1b* in BDM/microglial cells (outlined) after injury. Asterisks indicate the injury site in (B, C). Scale bars: 50 µm (B, C, F), 10 µm (D). Data files for graphs available in S2 Data.

To detect *plexin b1* receptors expression *in vivo*, we performed multiplexed HCR-FISH for *plxnb1a* and *plxnb1b* in combination with the *her4.1:EGFP* transgenic reporter line, to label ERGs [65]. *plxnb1a* mRNA was detectable at low levels in ERGs in unlesioned animals at 3 dpf (Fig 4A and 4B). At 24 hpl, the signal appeared slightly weaker, as predicted by lower expression levels in scRNA-seq (see S1G Fig). The signal for *plxnb1b* was generally stronger in ERGs (*her4.1:EGFP+*) than that for *plxnb1a* both before and after lesion (Fig 4C). High magnifications indicated co-expression of *plxnb1a and plxnb1b* in the same ERGs in unlesioned and lesioned animals. Notably, outside the ERGs only very low (*plxnb1a*) or no signal (*plxnb1b*) was detectable in spinal tissue (Fig 4D). Quantifications of *plxnb1a* and *plxnb1b* comparing unlesioned and lesioned fish showed a small (24%) reduction in expression of *plxnb1a* after injury, but no changes in *plxnb1b* (Fig 4E). This is also predicted by Cavone *her4.1:eGFP*-enriched scRNA-seq dataset [42]. In the non-neural lesion site, BDMs/microglia (*mpeg1:mCherry*+) were also negative for *plxnb1a and plxnb1b* (Fig 4F). This indicated that transcripts for the paralogous *plxnb1a* and *plxnb1b* were enriched in ERGs, as predicted by scRNA-seq (see S1I Fig).

### *sema4ab* disruption increases regenerative neurogenesis

For a loss-of-function analysis of *sema4ab* in regenerative neurogenesis, we targeted *sema4ab* in somatic mutants by injecting haCRs into fertilized eggs. We controlled each experiment by an internal control RFLP to show >90% effectiveness of guides in destroying a targeted restriction enzyme recognition site (S4A Fig). Further evidence for the effectiveness of gene disruption comes from the complete disappearance of correct DNA sequences in scRNA-seq analysis (see above and see S3 and S4 Tables). Potential stress responses to the injection are controlled for by always using control gRNA injections in internal controls for each experiment [66]. Somatic targeting reduces mRNA detectability of *sema4ab* in qRT-PCR by 50%, indicating that gene disruption perturbed mRNA transcription (S4A Fig). Finally, we raised a germline stable mutant for *sema4ab* to verify gene disruption phenotypes. The germline mutant introduced a 14 bp insertion in exon 6, leading to a premature stop codon, which is predicted to lead to a truncated protein lacking functionally important domains, such as sema superfamily domains, plexin repeat domains and the intracellular part, containing a sema4F domain (S4B Fig).

Somatic mutants for *sema4ab* showed no differences in growth compared to control gRNA injected larvae, as measured by body length (S4C–S4E Fig), notochord thickness (S4F Fig), and spinal cord thickness (S4G Fig). However, a small decrease in eye size was observed (11% reduction, S4H Fig). Germline mutants showed no change in body length (S4I–S4K Fig) and replicated the reduction in eye diameter seen in somatic mutants (9% reduction; S4L Fig). Slightly reduced eye size may be related to compromised photoreceptor maintenance in the absence of Sema4a function [67]. Hence, *sema4ab* disruption was effective in somatic and germline mutants and gross development of larvae was largely unimpaired in the absence of *sema4ab*.

**Fig 5:**
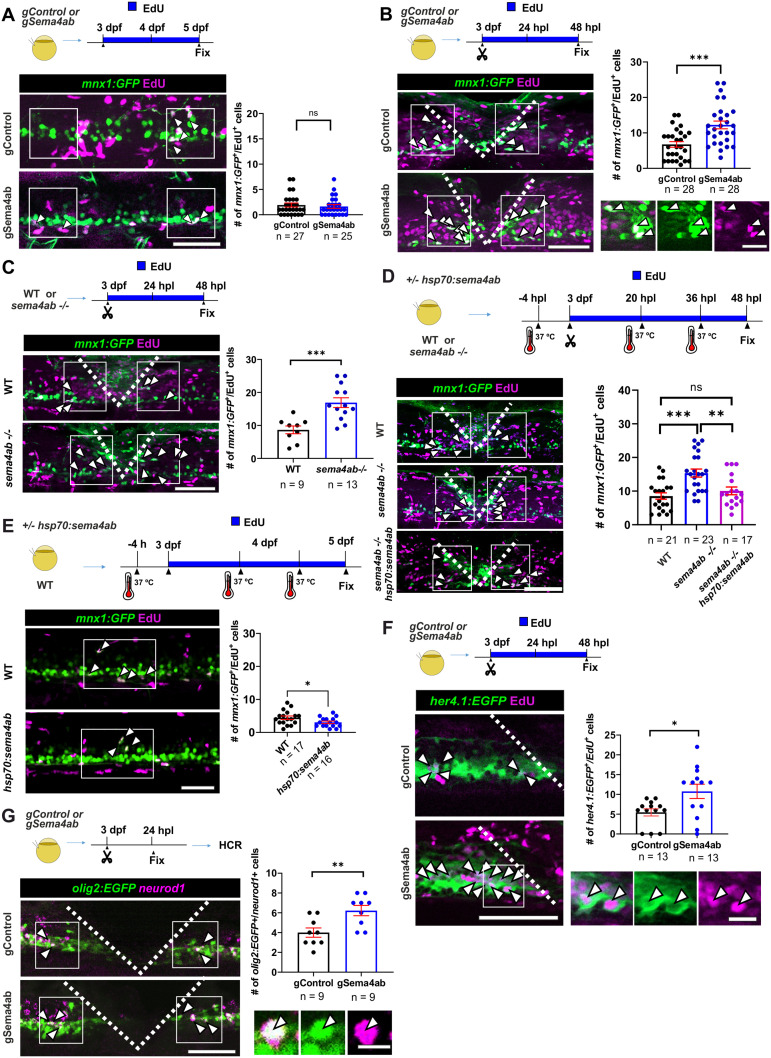
*Sema4ab* loss of function increases regenerative neurogenesis. **(A)** Ongoing developmental neurogenesis of motor neurons (*mnx1:GFP*+/EdU+) is not different between gControl- and gSema4a-injected unlesioned larvae (gControl: 1.9 cells per larvae ± 0.3920; gSema4ab: 1.7 cells per larvae ± 0.3646; Mann–Whitney *U* test: *p* = 0.5972). **(B)** Lesion-induced generation of motor neurons (*mnx1:GFP*+/EdU+) is increased in somatic mutants for *sema4ab* (gControl: 6.9 cells per larva ± 0.8; gSema4ab: 12.9 cells per larva ± 1.1; Mann–Whitney *U* test: *p* = 0.0001). (**D)** Lesion-induced generation of motor neurons (*mnx1:GFP*+/EdU+) in germline mutants for *sema4ab* is also increased (WT: 8.7 cells per larva ± 1.2; *sema4ab*−/−: 16.9 cells per larva ± 1.5; Mann–Whitney *U* test: *p* = 0.0001). **(D)** Overexpression of *sema4ab* driven by a heat-shock promotor (*hsp70:sema4ab*) reduces over-production of motor neurons (*mnx1:GFP*+/EdU+) back to wild type levels in *sema4ab* germline mutants (WT: 8.5 cells per larva ± 0.95; *sema4ab*−/−: 15.4 cells per larva ± 1.2; *sema4ab*−/− +  *hsp70:sema4ab*: 10 cells per larva ± 1.15; Kruskal–Wallis test: p = 0.0004; Dunn’s multiple comparisons test: ***p = 0.006; n.s > 0.9999, **p = 0.0168). All conditions shown were heat-shocked. **(E)** Overexpression of *sema4ab* in unlesioned larvae reduces ongoing developmental neurogenesis of motor neurons (*mnx1:GFP*+/EdU+) (WT: 4.4 cells per larva ± 0.52; *hsp70:sema4ab*: 3.0 cells per larva ± 0.37; Unpaired *t* test: *p* = 0.0374). **(F)** Lesion-induced proliferation of ERGs (*her4.1:EGFP+*/*EdU+*) is increased in somatic mutants for *sema4ab* (gControl: 5.4 cells per larva ± 0.9; gSema4ab: 10.77 cells per larva ± 1.8; Mann–Whitney *U* test: *p* = 0.0219). **(G)** The number of *neurod1*-labeled motor neuron progenitor cells (*olig2:EGFP+/neurod1+*) is increased after *sema4ab* disruption (gControl: 4.0 cells per larvae ± 0.4714; gSema4ab: 6.2 ± 0.5212 cells per larvae; Unpaired *t* test: *p* = 0.0060). Error bars show SEM. Scale bars: 50 µm (A, B, C, D, E, F, G), 10 µm (insets). Data files for graphs available in S2 Data.

To analyze regenerative neurogenesis in somatic and germline stable mutants, we used 5-Ethynyl-2′-deoxyuridine (EdU) labeling in the *mnx1:GFP* transgenic line that indicates motor neurons [68]. Motor neurons represent the main cell type that is newly generated during lesion-induced neurogenesis [42]. In unlesioned *sema4ab* haCR-injected somatic mutant larvae (EdU added at 72 hpf, analysis at 96 hpf) few newly generated motor neurons (EdU+/*mnx1*:*GFP*+) were present, similar to control gRNA-injected larvae (Fig 5A). This was expected, since *sema4ab* was not detectable in the spinal cord without lesion, and shows that *sema4ab* does not have a detectable role in ongoing low-level developmental neurogenesis.

After a lesion at 3 dpf, we observed a ~ 4-fold lesion-induced increase in the number of new motor neurons in control gRNA-injected larvae in the vicinity of the lesion at 48 hpl, as previously reported [14]. Remarkably, in *sema4ab* haCR injected larvae, this number was further increased by 85%, compared to lesioned wild type control gRNA-injected larvae (Fig 5B). Similarly, in the lesioned *sema4ab* germline mutant, the number of new motor neurons almost doubled (95% increase) compared to lesioned wild type control larvae (Fig 5C). This result is in agreement with the prediction by scRNA-seq of increased neurogenesis after *sema4ab* disruption.

To determine whether this phenotype is likely due to *sema4ab* loss-of-function, we over-expressed the protein in *sema4ab* germline mutants to see a possible rescue effect. To that aim, we generated a construct that leads to marked overexpression of *sema4ab* and a fluorescent reporter under the control of a heat-shock promotor (*hsp70l:sema4ab-t2a-mCherry,* abbreviated as *hsp70:sema4ab)* in heat-shocked plasmid-injected, but not in control animals (S5A–S5D Fig). Indeed, over-expression in the *sema4ab* germline mutant reduced the number of newly generated motor neurons to that observed in wild type controls (Fig 5D), indicating a phenotypic rescue. Over-expression of *sema4ab* in lesioned wildtype animals did not alter the number of newly generated motor neurons (S5E Fig), which might have been due to a ceiling effect for *sema4ab* expression. We therefore over-expressed *sema4ab* in unlesioned wildtype animals in which no detectable *sema4ab* expression is present. Indeed, we observed a 32% reduction in ongoing developmental neurogenesis (Fig 5E). This supports the assumption that there was a ceiling effect in lesioned animals and it shows that progenitors do not need any lesion signal to react to Sema4ab. The phenotypic rescue in the *sema4ab* mutant confirmed that the germline mutant carried a *loss-of-function* mutation, and that *sema4ab* was a strong negative regulator of neurogenesis.

To determine how the increased number of new motor neurons observed after *sema4ab* disruption was related to progenitor proliferation, we cumulatively labeled *her4.1:EGFP*-expressing ERGs with EdU (Fig 5F). This indicated a doubling (97% increase) in EdU-labeled ERGs (*her4.1:EGFP*+/EdU+) in lesioned *sema4ab* somatic mutant larvae, compared to control-gRNA injected lesioned larvae. This suggests that *sema4ab* negatively affects progenitor proliferation.

To determine whether there was a change in the number of ERGs undergoing neuronal differentiation, we analyzed the expression of the proneural transcription factor *neurod1* [69] in motor neuron progenitor cells by HCR-FISH. The number of *neurod1*-expressing motor neuron progenitor cells (*olig2:EGFP+/neurod1+*) was increased by 50% in lesioned somatic mutants for *sema4ab*, compared to lesioned control-gRNA injected larvae, at 24 hpl (Fig 5G). Hence, in the absence of *sema4ab* function, the number of EdU label-retaining progenitor cells, the number of neurogenic progenitors, and newly generated neurons were all increased. This is consistent with scRNA-seq data and indicates that in unperturbed regeneration, *sema4ab* restricts proliferation of neurogenic progenitors to reduce regenerative neurogenesis.

### sema4ab disruption impairs recovery of swimming function and axon regrowth

**Fig 6:**
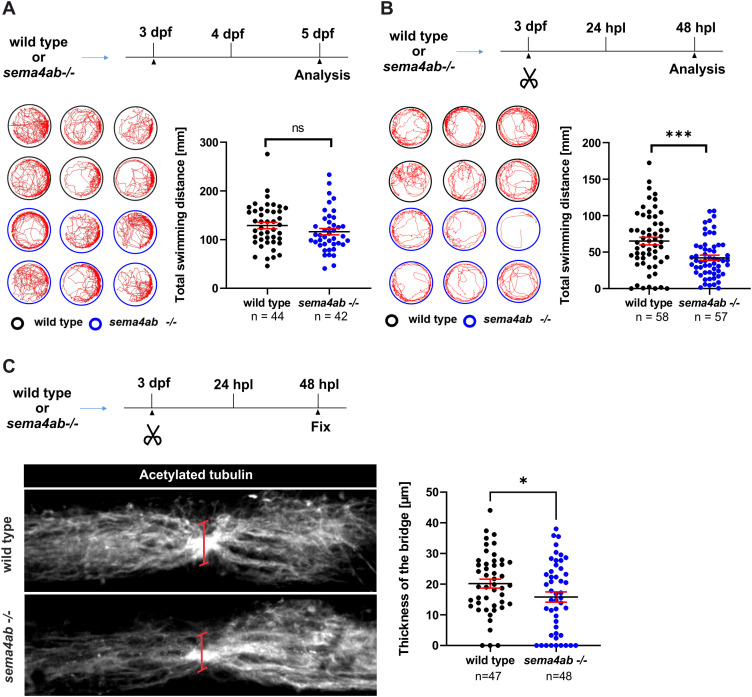
Disruption of *sema4ab* impairs recovery of swimming behavior. **(A)** A test of swimming behavior at 5 dpf in unlesioned larvae shows no change in swimming distance in *sema4ab* germline mutants, compared to wildtype controls (wild type: 128.8 mm/min ± 6.657; *sema4ab* −/−: 116.2 mm/min ± 6.504; Unpaired *t* test: *p* = 0.1798). **(B)** A test of swimming behavior at 48 hpl in 5 dpf larvae shows a reduction in swimming distance in *sema4ab* germline mutants (wild type: 76.98 mm/min ± 8.846; *sema4ab* −/−: 42.15 mm/min ± 5.508; Mann–Whitney *U* test: *p* = 0.0005). **(C)** In *sema4ab* mutants the axonal fiber bundle crossing the injury site shows reduced thickness, compared to controls at 48 hpl (wild type: 20.20 µm ± 1.5; *sema4ab* −/−: 15.8 µm ± 1.7; one-tailed *t* test: *p* = 0.0253). Error bars show SEM. Scale bars: 50 µm. Data files for graphs available in S2 Data.

Behavioral recovery is an important aspect of spinal repair. Therefore, we assessed functional recovery by measuring the distance swum after a vibration stimulus. Uninjured *sema4ab* germline mutants swam distances that were not different from those of uninjured wild type larvae at 5 dpf (Fig 6A). In contrast, after a previous spinal cord transection at 3dpf, we observed a 35% reduction in swim distance in lesioned *sema4ab* germline mutants, compared to lesioned wild type animals (Fig 6B).

Since recovery from spinal injury strongly correlates with axon regrowth across the lesion [30], we tested whether axon regrowth would be reduced in *sema4ab* mutants. We assessed the thickness of the neuronal tissue bridge across the lesion by immunolabeling of axons re-connecting the spinal cord stumps in germline mutants for *sema4ab* (Fig 6C) [30]. Indeed, we observed a 25% reduction in the thickness of the axonal bridge at 48 hpl in lesioned *sema4ab* germline mutant animals, compared to lesioned wild type control animals. These results demonstrate that *sema4ab* does not detectably affect development of the locomotor system, but is necessary for efficient recovery of swimming function and axon regrowth after injury.

### Disruption of *plxnb1a/b* augments regenerative neurogenesis

**Fig 7:**
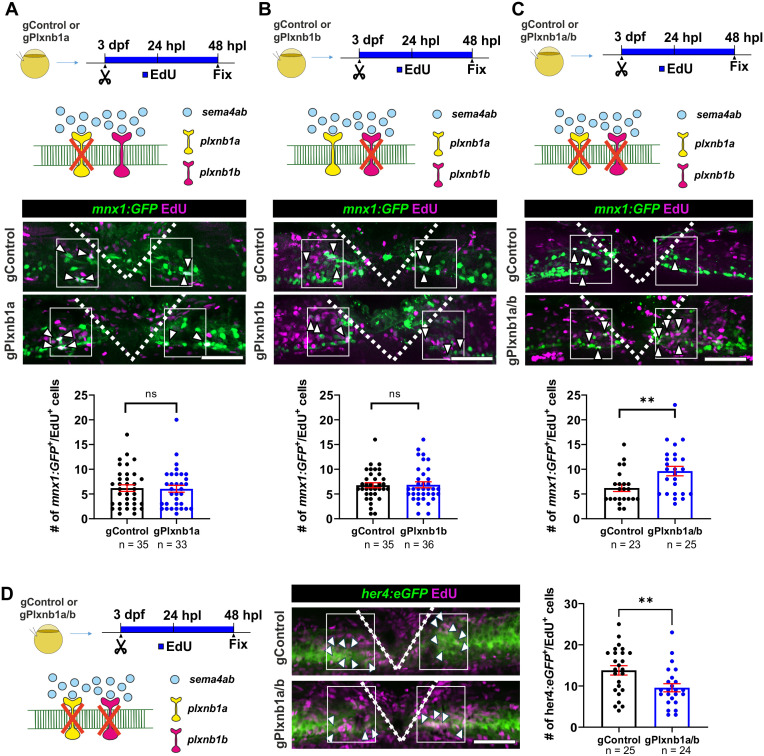
Combined *plxnb1a* and *plxnb1b* loss of function increases regenerative neurogenesis. Photomicrographs are oriented as in Fig 2. **(A,B)** Regenerative neurogenesis is not changed in single somatic mutants for *plxnb1a* (A, gControl: 6.2 cells per larva ± 0.66; gPlxnb1a: 6.1 cells per larva ± 0.71; Mann–Whitney *U* test: *p* = 0.8371) and *plxnb1b* (B, gControl: 6.8 cells per larva ± 0.52; gPlxnb1b: 6.9 cells per larva ± 0.60; Mann–Whitney *U* test: *p* = 0.6511). **(C)** Regenerative neurogenesis is increased in double somatic mutants of *plxnb1a and plxnb1b* (gControl: 6.2 cells per larva ± 0.71 cells per larva; gPlxnb1a/b: 9.6 cells per larva ± 0.96; Mann–Whitney *U* test: *p* = 0.0057). **(D)** Lesion-induced proliferation of ERGs (*her4.1:EGFP+*/*EdU+*) is decreased in double somatic mutants for *plxnb1a/b* (gControl: 5.4 cells per larva ± 0.9; gSema4ab: 10.77 cells per larva ± 1.8; Mann–Whitney *U* test: *p* = 0.0219). Error bars show SEM. Scale bars: 50 µm. Data files for graphs available in S2 Data.

Enriched expression of *plxnb1a* and *plxnb1b* in ERGs after injury made these good candidates for receiving a *sema4ab* signal. Therefore, we disrupted *plxnb1a* and *plxnb1b* by haCR injection. Internal RFLP controls confirmed high efficiency of the haCRs and mRNA expression was reduced by 55%–60% for these genes (S6A Fig). While single somatic mutations had no effect (Fig 7A and 7B), simultaneous mutation of *plxnb1a* and *plxnb1b* by co-injecting the respective haCRs increased numbers of newly generated motor neurons (EdU+/*mnx1:GFP*+) by 55% at 48 hpl (Fig 7C). Disruption of *plxnb2b*, which was also expressed in ERGs, and its paralog *plxnb2a* (S6B–S6E Fig), had no effect in regenerative neurogenesis (S6F–S6H Fig).

To determine whether disrupting *plxnb1a* and *plxnb1b* affected ERG proliferation, we cumulatively labeled *her4.1:EGFP*-expressing ERGs with EdU in lesioned animals in which both *plxnb1a* and *plxnb1b* were disrupted. We observed a 30% reduction in the number of EdU-labeled ERGs, compared to lesioned control gRNA-injected larvae (Fig 7D). Together, that indicated that *plxnb1a* and *plxnb1b* activity attenuated neuronal differentiation. Hence, disrupting *plxnb1a/b* partially replicated the effect of *sema4ab* mutation, with a lower increase in the number of newly generated neurons and, differently from *sema4ab* disruption*,* a decrease in the number of EdU-labeled ERGs. This suggested that while some direct signaling from microglia to ERGs was likely, there was probably also a major indirect effect of *sema4ab* on ERGs via the lesion site environment.

### Disruption of *sema4ab* reduces the number of microglia but does not change numbers of other invading cell types

To characterize the influence of *sema4ab* on the lesion site environment *in situ*, we analyzed the numbers of cells invading the lesion site with known roles in regeneration, namely neutrophils [70], microglia, BDMs [71], and fibroblast-like cells [37,30]. The number of neutrophils (*mpx:GFP*+) in the lesion site was unchanged between lesioned control gRNA-injected larvae and *sema4ab* haCR-injected larvae at 4 hpl (peak of neutrophil invasion, [71] and 24 hpl (S7A–S7C Fig). Also, the density of fibroblasts (*pdgfrb:GFP+*) also remained unchanged in the absence of *sema4ab* function at 24 hpl (S7D Fig).

**Fig 8:**
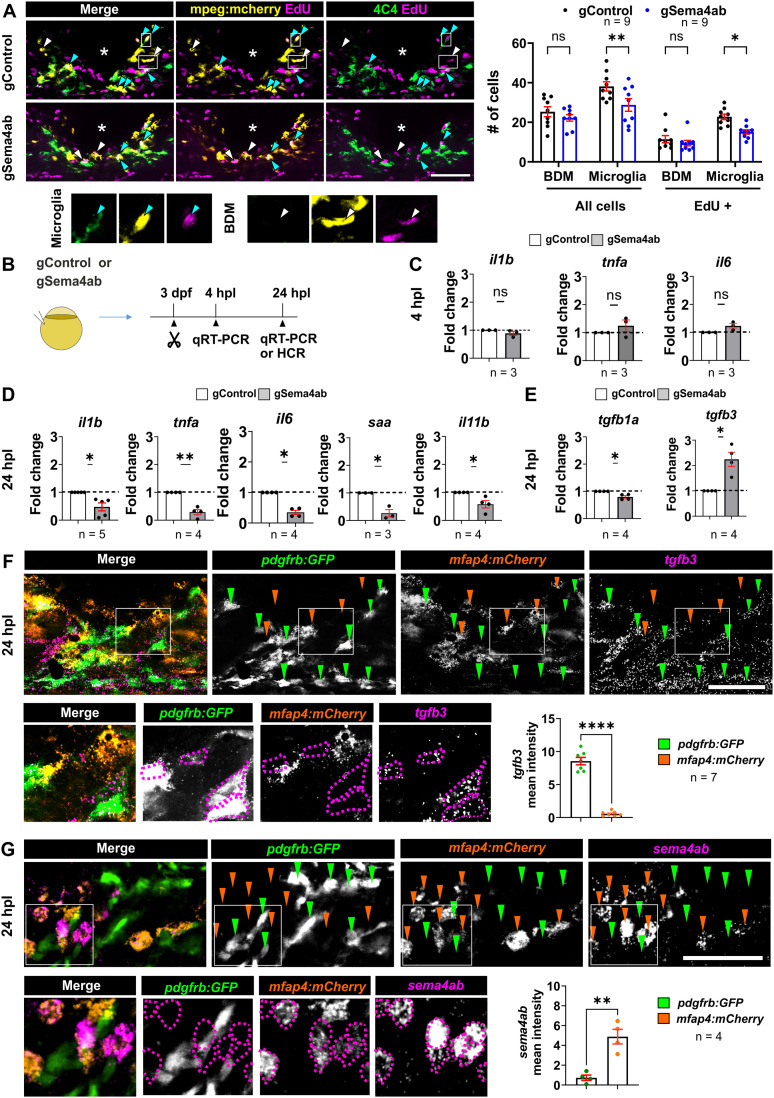
Loss of *sema4ab* function in microglia changes the cytokine profile of the lesion site and increases *tgfb3* expression fibroblasts. **(A)** There is a reduction in microglia cell number (gControl: 38.11 ± 2.3 cells per larvae, Microglia 28.78 ± 3.2 cells per larvae) but not for BDMs (gControl: 25.33 cells per larvae ± 2.5, gSema4ab: 22.22 cells per larvae ± 1.6) in sema4ab somatic mutants. The number of EdU-incorporating cells (incubation from lesion at 3 dpf to analysis at 24 hpl) is also reduced for microglia (gControl 22.8 ± 1.4 cells per larvae; gSema4ab 15.11 ± 1.1 cells per larvae), but not BDMs (gControl 11.4 ± 1.8 cells per larvae; gSema4ab 9.4 ± 1.4 cells per larvae) (Šídák’s multiple comparisons test: BDM *p* = 0.7388; Microglia *p* = 0.0077, BDM/EdU *p* = 0.9328, Microglia/EdU *p* = 0.0392). **(B)** A schematic indicating the experimental design for cytokine quantification by qRT-PCR is shown. **(C)** qRT-PCR analyses of major pro-inflammatory cytokines in *sema4ab* somatic mutants show no differences to injured control gRNA-injected larvae at 4 hpl (One-sample *t* test; *il1b*: *p* = 0.2526; *tnfa*: *p* = 0.3623; *il6*: *p* = 0.1028). **(D)** At 24 hpl, *sema4ab* somatic mutants show decreased expression levels of pro-inflammatory cytokines and markers (One-sample *t* test*; il1b*: −2.12 fold-change, *p* = 0.0212; *tnfa*: −3.58 fold-change, *p* = 0.0047; *il6*: −2.94 fold-change, *p* = 0.0024; *saa*: −3.84 fold-change, *p* = 0.0311; *il11b*: −1.73 fold-change, *p* = 0.0495). **(E)** While the anti-inflammatory cytokine *tgfb1a* shows a slight decrease (−1.26 fold-change, One-sample *t* test; *p* = 0.0251), *tgfb3* is substantially increased in expression in *sema4ab* somatic mutants (+2.24 fold-change, One-sample *t* test; *p* = 0.0210). (**F)** HCR-FISH against *tgfb3* in the *pdgfrb:GFP* × *mfap4:mCherry* double transgenic line shows expression of *tgfb3* in *pdgfrb:GFP*+ cells (green arrowheads) and hardly in *mfap4:mCherry*+ cells (orange arrowheads) (*pdgfrb:GFP*: 8.557 [a.u]; *mfap4:mCherry:* 0.5726 [a.u]; Unpaired *t* test: *p* < 0.0001). Purple dotted circles point at *tgfb3*-expressing cells. **(G)** HCR-FISH against *sema4ab* in the the *pdgfrb:GFP* × *mfap4:mCherry* double transgenic line shows hardly any expression of *sema4ab* in *pdgfrb:GFP*+ cells (green arrowheads), but in *mfap4:mCherry*+ cells (orange arrowheads) (*pdgfrb:GFP*: 0.7313 [a.u]; *mfap4:mCherry:* 4.883 [a.u]; Unpaired *t* test: *p* = 0.0019). Purple dotted circles point at *sema4ab*-expressing cells. Each dot for qRT-PCR represents a pool of 50 larvae. β-actin was used as housekeeping gene. Raw data for qRT-PCR can be found in S5 and S6 Data. Error bars show SEM. Scale bars: 50 µm. Data files for graphs available in S2 Data.

For BDMs and microglia (*mpeg1:mCherry*+) we observed a moderate reduction in number by 12% in *sema4ab* haCR injected larvae (S7E Fig) at 24 hpl. To determine whether microglia and BDMs would be differently affected in the absence of *sema4ab*, we separately quantified BDMs and microglia by co-labeling with the 4C4 microglia marker antibody [32]. This showed that BDMs (*mpeg1:mCherry+/*4C4−) did not change in cell numbers, whereas microglia numbers (*mpeg1:mCherry+/*4C4+) were reduced by 25% (Fig 8A).

We also assessed potential changes in proliferation of BDMs and microglia using cumulative EdU labeling in combination with immunohistochemistry with 4C4 labeling in the *mpeg1:mCherry* reporter line (Fig 8A). This showed that 44% of BDMs and 57% of microglia were newly generated in control animals after injury. The number of newly generated BDMs (*mpeg1:mCherry+*/ EdU+ /4C4−) was not changed in *sema4ab* somatic mutants after lesion. In contrast, the number of newly generated microglia (*mpeg1:mCherry+*/EdU+/4C4+) was reduced by 33%. Hence, microglia numbers were reduced, likely related to impaired proliferation in the absence of *sema4ab.*

We next analyzed phagocytosis of cellular debris, which is one of the major behaviors of BDMs/microglia [72]. We used acridine orange staining to detect levels of cellular debris as a proxy for efferocytosis. The average fluorescence intensity of acridine orange in the lesion site was comparable between control gRNA-injected larvae and *sema4ab* somatic mutants at 24 hpl (S7F Fig). Similarly, the number of BDMs/microglia with acridine orange-positive inclusions, as indicators of phagocytosed debris, did not show differences in these experimental groups (S7G Fig). This indicates that efferocytosis of debris by BDMs/microglia was not quantitatively altered in *sema4ab-*deficient animals.

### Cytokine signaling is altered in *sema4ab*-deficient animals

Changes in cytokine levels, as indicated by our scRNA-seq analysis, can have profound effects on cell behavior. Additionally, changes in immune cell numbers, like a microglia reduction, can affect the overall cytokine release in the injury site and condition the inflammatory milieu. Therefore, we analyzed expression levels of *serum amyloid A* (*saa*), an inflammation marker and regulator of cytokine release [73], and major pro- (*il1b, tnfa, il6, il11b*) and anti- (*tgfb1a, tgfb3*) inflammatory cytokines [74] in the lesion site environment in control gRNA- and *sema4ab* haCR-injected lesioned larvae by qRT-PCR at 4 and 24 hpl (Fig 8B). *il1b*, *tnfa*, and *il6* did not show any changes at 4 hpl (Fig 8C), when the immune response was dominated by neutrophils. However, reduced expression levels of pro-inflammatory cytokines *il1b* (by 50%), *tnfa* (by 75%), and *il6* (by 65%), together with those of *il11b* (by 45%) and *saa* (by 75%), were detected at 24 hpl (Fig 8D), when microglia dominated the injury site. At that time point, levels of *tgfb1a* expression were slightly reduced (by 20%), whereas those of *tgfb3* were more than doubled (120% increase; Fig 8E). In histology, overall fluorescence intensity for *il1b:GFP* and *tnfa:EGFP* was likewise reduced in *sema4ab* somatic mutants (S8A–S8C Fig). There was also a reduction (11%) in the labeling intensity for *il1b* specifically in microglia (*apoeb+,* S8D Fig). Overall, a combination of a reduction in microglia number, activation state, and influence on other lesion site cells led to a shift from a pro-inflammatory to an anti-inflammatory lesion environment in the absence of *sema4ab* function.

Interestingly, after co-disruption of *plxnb1a/b,* cytokine expression levels of *il1b, tnfa, il6 and tgfb3* remained unchanged in these animals in qRT-PCR (S8E and S8F Fig). Hence, *plxnb1a/b* do not mediate the *sema4ab*-dependent changes in the inflammatory milieu, supporting that their action on neurogenesis could be due to ligand-receptor interactions in ERGs and not by changing the inflammatory milieu of the lesion.

### Fibroblasts increase *tgfb3* expression in the absence of *sema4ab* function in immune cells

Next, we analyzed the cell type(s) expressing *tgfb3*, since scRNA-seq predicted the cytokine not to be strongly expressed in immune cells. In HCR-FISH, the labeling intensity of *tgfb3* mRNA was generally increased in *sema4ab* somatic mutants compared to control gRNA-injected controls, in agreement with qRT-PCR results (S9A Fig). Remarkably, expression of *tgfb3* in fibroblasts (*pdgfrb*+), but not in BDMs or microglia was observed in scRNA-seq (see Fig 2F). In agreement with scRNA-seq, in HCR-FISH co-localization studies the *tgfb3* signal was hardly detectable in BDM/microglia cells (*mfap4*:*mCherry*+). Instead, the signal was present mostly in fibroblasts (*pdgfrb:GFP*+; Fig 8F) and increased by 20% in the absence of *sema4ab* function (S9B Fig). Conversely, *sema4ab* was hardly detectable in fibroblasts in the lesion by HCR-FISH, but in *mfap4:mCherry*+ cells (Fig 8G). This suggested a scenario in which predominant expression of *sema4ab* in microglia influences the lesion site environment in such a way that *tgfb3* production by fibroblasts is reduced.

Indeed, live observation of BDMs/microglia (*mfap4:mCherry*) and fibroblasts *(pdgfrb:GFP)* from 10 to 25 hpl indicated close physical interactions between these cell types in the injury site, where *mfap4:mCherry+* cells move along elongated stationary fibroblasts (S9C and S9D Fig, S1 and S2 Movies). No obvious differences in these cell behaviors were detectable between control gRNA- and *sema4ab* haCR-injected larvae. These data suggest that BDMs/microglia are in close contact with fibroblasts and may modify the microenvironment, such that *tgfb3* expression in fibroblasts is attenuated in a *sema4ab*-dependent manner.

To determine whether microglia were indeed responsible for attenuating *tgfb3* expression, we genetically depleted microglia using the *csf1ra/b* double mutant line, in which disruption of *csf1r* receptors lead to a strong impairment in microglia development and recruitment to a spinal lesion [71,75], which we confirm here (S10A and S10B Fig). In this mutant, we observed an 1.9-fold increase in *tgfb3* levels in lesioned *csf1ra/b* double mutants, compared to lesioned wild type larvae, which was comparable to the consequences of *sema4ab* loss of function (S10C Fig). This observation is consistent with microglia negatively impacting on *tgfb3* expression in fibroblasts.

To determine whether microglia may have affected *tgfb3* expression via the observed increase in the expression of major pro-inflammatory signals *il1b* and *tnfa* after *sema4ab* disruption, we disrupted these pathways by YVAD treatment (inhibition of IL-1b processing; [71]) and in *tnfrsf1a* (expressed by fibroblasts; S10D–S10F Fig; [42]) somatic mutants. However, these manipulations failed to change *tgfb3* expression levels in lesioned animals at 24 hpl, as measured by qRT-PCR, indicating more complex potential interactions between microglia and fibroblasts. This could be via additional cytokine receptors expressed by fibroblasts (S10F Fig).

### *tgfb3* is enriched in specific fibroblasts clusters and in close contact with ERGs and growing axons

To characterize *tgfb3*-expressing fibroblasts, we analyzed the fibroblast clusters defined by unsupervised clustering in our scRNA-seq dataset (S11A Fig), and we performed an analysis of the most highly enriched gene expression per cluster (S11B Fig). We found that the 7 clusters of fibroblasts were differentially enriched in marker genes (S11C Fig; see S4 Data for citations). Based on these, we tentatively annotated them as: ECM-associated fibroblasts (FB#1; enriched marker genes: *hapln1b, HAPLN1, fbln1c*), mesenchymal fibroblasts (FB#2; enriched marker genes: *six1b, eya1, eomesa*), perivascular fibroblasts (FB#3; enriched marker genes: *foxc1a, fixd1, col18a1b, cldn11a*), signaling-related fibroblasts (FB#4; enriched marker genes: *fgf8a, fgf8b, fsta*), signaling-related fibroblasts 2 (FB#5; enriched marker genes*: asip1, agrp, grem1a*), immune-related fibroblasts (FB#6; enriched marker genes: *masp1, fgl2, COLEC10, tnfsf14*) and myofibroblasts (enriched marker genes: *acta2, desmb, cald1b, mylkb, pdlim3a*). Among these clusters, those representing mesenchymal fibroblasts, signaling-related 1 fibroblasts, immune-related fibroblasts, and myofibroblasts showed enriched expression of *tgfb3* (S11D Fig). However, scRNA-seq did not indicate specific increases in expression or cell numbers after *sema4ab* disruption, making it difficult to positively identify the relevant sub-population(s).

To assess whether *tgfb3*-expressing fibroblasts were close to ERGs and axons to potentially directly affect these, we performed histology and *in vivo* imaging. *tgfb3*-expressing fibroblasts (*tgfb3+/pdgfrb:GFP*) were found throughout the thickness of the trunk tissue by HCR, including in the lesion, where axons grow, and on the surface of the spinal stumps, which contained the ERGs (S11E Fig). In live the double-transgenic animals, in which fibroblasts and axons are labeleld (*pdgfrb:GFP x elavl3:mkate*), we observed axons in close contact with fibroblasts (S11F Fig). In double transgenic animals labeling fibroblasts and motor neuron progenitors (*pdgfrb:GFP x olig2:dsRed*), we found fibroblasts in the vicinity of ERGs (S11G Fig). This is consistent with potential diffusible signaling of *tgfb3* to both axons and ERGs.

### Fibroblasts promote regenerative neurogenesis via *tgfb3*

**Fig 9:**
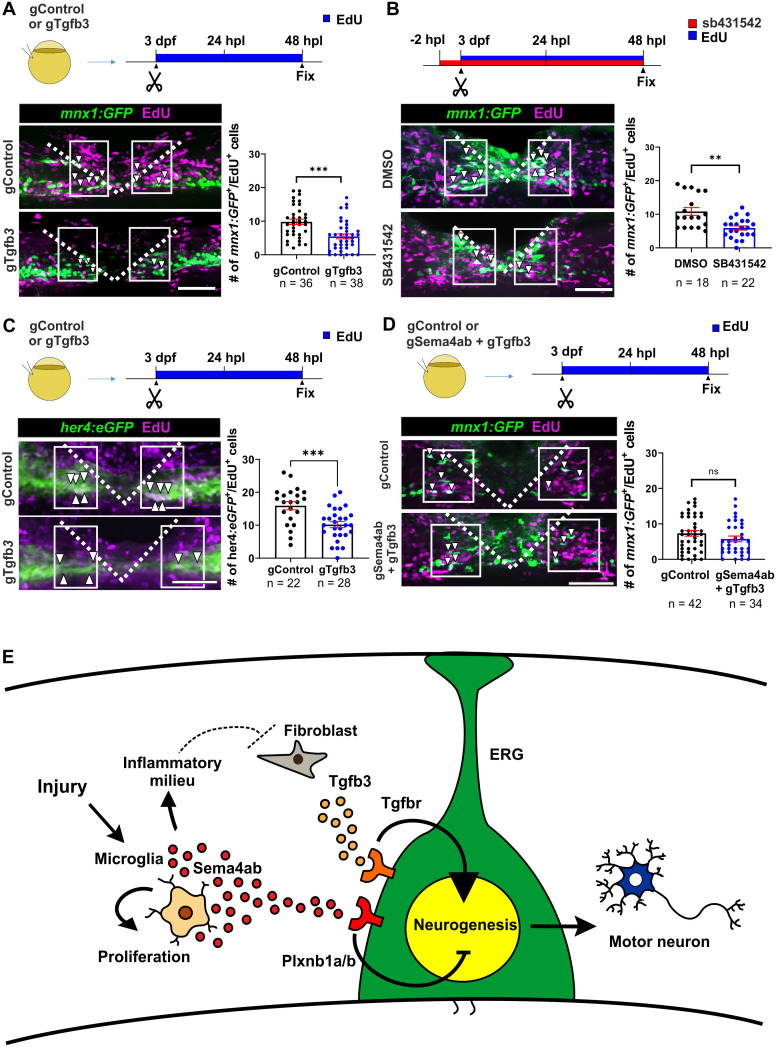
Fibroblasts promote regenerative neurogenesis via *tgfb3.* **(A)** Somatic mutants for *tgfb3* show a reduction in the number of newly generated motor neurons (gControl: 9.8 cells per larva ± 0.8; gTgfb3: 5.5 cells per larva ± 0.7; Mann–Whitney *U* test: *p* = 0.0001). **(B)** SB431542 treatment reduces regenerative neurogenesis (DMSO: 10.8 cells per larva ± 1.15; SB431542: 5.9 cells per larva ± 0.63; Mann–Whitney *U* test: *p* = 0.0011). **(C)** Lesion-induced proliferation of ERGs (*her4.1:EGFP+*/*EdU+*) is decreased in somatic mutants for *tgfb3* (gControl: 15.91 cells per larva ± 1.2; gTgfb3: 10.18 cells per larva ± 0.9; Unpaired *t* test: *p* = 0.0004). **(D)** Double haCR injection against *sema4ab* and *tgfb3* restores control levels of regenerative neurogenesis (gControl: 8.1 cells per larva ± 0.7; gSema4ab+gTgfb3: 6.6 cells per larva ± 0.8; Mann–Whitney *U* test: *p* = 0.1523). **(E)** A graphical summary of proposed interactions of *sema4ab* during spinal cord regeneration is shown. After spinal injury, microglia likely control neurogenesis directly by signaling through Sema4ab and Plxnb1a/b on ERGs, and indirectly by changing the injury site environment, which leads to lower expression of *tgfb3*, a positive regulator of regenerative neurogenesis, in fibroblasts. Additionally, Sema4ab controls microglia number by promoting cell proliferation. Error bars show SEM. Dotted lines show the injury site. Scale bars: 50 µm. Data files for graphs available in S2 Data.

The observed increase of *tgfb3* expression levels in fibroblasts after *sema4ab* disruption could affect neurogenesis [76]. To test the role of *tgfb3* in regenerative neurogenesis in the zebrafish spinal cord, we effectively disrupted the gene in somatic mutants, as shown previously [48]. We verified effective gene disruption here by demonstrating resistance of the target site of the haCR to restriction enzyme digestion in RFLP and by showing a 70% reduced *tgfb3* mRNA expression in qRT-PCR (S9E Fig). In addition, we used a temporally conditional way to reduce Tgfb signaling by pharmacologically blocking Tgfb receptors with the small molecule inhibitor SB431542 [77]. Somatic mutants for *tgfb3* showed a 45% reduction in the number of newly generated motor neurons (EdU+/*mnx1:GFP*+) after spinal lesion, compared to control gRNA-injected lesioned larvae at 48 hpl (Fig 9A). Similarly, adding SB431542 to lesioned larvae reduced the number of newly generated motor neurons by 45%, compared to DMSO treated controls, at 48 hpl (Fig 9B).

To determine whether disrupting *tgfb3* also affected ERG proliferation, we cumulatively labeled *her4.1:EGFP*-expressing ERGs with EdU in *tgfb3 somatic* mutants (Fig 9C). We observed a 35% reduction in the number of EdU-labeled ERGs. These observations support a promoting role of *tgfb3* for regenerative neurogenesis by promoting ERG progenitor cell proliferation. The results are also consistent with the observed increase in ERG proliferation after *sema4ab*, but not *plxnb1a/b,* disruption, potentially by increasing *tgfb3* expression.

### Disruption of *tgfb3* abolishes the effect of *sema4ab* disruption on lesion-induced neurogenesis

To determine whether control of *tgfb3* expression by Sema4ab signaling could explain at least in part the role of *sema4ab* in attenuating regenerative neurogenesis, we disrupted both genes simultaneously by co-injecting *sema4ab* and *tgfb3* haCRs. Disruption of both genes was effective, as indicated by internal RFLP controls for both targets. In these larvae, lesion-induced generation of motor neurons (EdU+/*mnx1:GFP+*) was comparable to those in lesioned control gRNA-injected larvae (Fig 9D), fully abolishing the enhancing effect of *sema4ab* disruption on neurogenesis. These data show that *tgfb3* is a strong neurogenesis-promoting signal in fibroblasts and that it is controlled via *sema4ab,* which is predominantly expressed in microglia.

Interestingly, the reduction of neurogenesis when *sema4ab* and *tgfb3* were co-disrupted was stronger than expected, given the potential contribution of a *sema4ab* to *plxnb1a/b* direct signaling axis, which would still be disrupted in the absence of *sema4ab*. A likely reason for that was that haCRs almost completely disrupted gene function and could not be titrated to match expression levels of *tgfb3* present in control lesioned animals. Overall, these observations suggest that control of *tgfb3* expression in fibroblasts is a major mechanism by which *sema4ab*^*+*^ microglia attenuate lesion-induced neurogenesis.

### Potential *sema4ab* signaling in adult zebrafish and mouse spinal cord injury

To determine whether cell type-specific expression of *sema4ab, plexins* and *tgfb3* is conserved across ontogeny and phylogeny, we analyzed expression of relevant genes in published scRNA-seq profiles of the lesioned spinal cord of adult zebrafish [26] and adult mice [78].

In adult zebrafish, we found expression of *plxnb1a* and *plxnb1b* in clusters of *her4.1*+ ERGs. In scRNA-seq data, *sema4ab* reads were present in microglia (*p2ry12+*)*. tgfb3* reads were present in fibroblasts (*pdgfrb+*), but not in immune cells (S12A and S12B Fig). We confirmed expression of *plxnb1a and plxnb1b* this by HCR-FISH in cross sections of adult spinal cord, where we found expression in the ventricular layer (S12C Fig).

Similarly, in the sample from the lesioned spinal cord of adult mice, we found selective expression of *Sema4A* in microglia (*P2ry12+*) and *Plxnb1* selectively in potential ventricular progenitors (*Foxj1+*). *Tgfb3* was expressed in fibroblasts (*Pdgfra+*), but not in microglia (S13A and S13B Fig). This indicated that in the injured adult zebrafish and mouse spinal cords, the relevant genes are expressed in comparable cell types. However, whether expression levels and dynamics are sufficient for similar interactions between cell types as we observe in larval zebrafish to occur in other systems needs to be directly tested.

In summary, we propose a model by which Sema4ab from microglia controls neurogenesis directly through Plxnb1a/b on ERGs and indirectly via control of fibroblast-derived Tgfb3 in successful spinal cord regeneration in zebrafish (Fig 9E).

## Discussion

Here, we elucidate a mechanism by which regenerative neurogenesis is controlled in a regeneration-competent vertebrate. We show that *sema4ab*, mainly expressed by microglia, influences several cell types. We provide evidence for major functions of Sema4ab in preventing ERG progenitor over-proliferation directly via Plxnb1a/b receptors on ERGs and indirectly, by inhibiting Tgfb3 signaling from fibroblasts.

Even though mostly regeneration-promoting signals are reported for regenerating systems, regulation of growth is indispensable for appropriate repair. Here we show that expression of *sema4ab* attenuates regenerative neurogenesis. Some other attenuating signals have also been reported. For example, notch signaling is re-activated after spinal injury in adult zebrafish and attenuates neurogenesis there [79] and *myostatin a* is a secreted factor by spinal ventricular cells that also attenuates regenerative neurogenesis [31]. In a recent functional screen in the regenerating retina of adult zebrafish, 11 out of 100 regeneration-associated genes have been found to promote and 7 genes to attenuate neuronal regeneration [80]. Interestingly, when a stroke is induced or Notch signaling is disrupted, limited neurogenesis can be elicited from ependymal cells in the lateral ventricles of mice, but this leads to depletion of the ependymal cells [22]. Hence, attenuating factors are likely needed to keep the balance between progenitor maintenance and neuronal differentiation in successful regenerative neurogenesis.

Microglia may signal directly to ERGs to attenuate regenerative neurogenesis. Both signal (*sema4ab*) and receptors (*plxnb1a/b*) were expressed with high selectivity in microglia and ERGs, respectively, as indicated by HCR-FISH. However, other cell types may also express low levels of these genes, as suggested by scRNA-seq. Direct signaling from BDMs to ERGs has been shown for Tnfa, but with a promoting effect on neurogenesis [42]. In mammals, microglia also attenuate regenerative neurogenesis in the subventricular zone and hippocampus, which is related to the microglia’s pro-inflammatory status and may in part explain the poor regenerative outcome there [81–83].

Sema4ab interactions may affect regenerative neurogenesis in two ways, firstly by attenuating neuronal differentiation via *plxnb1a/b* expression on ERGs and secondly by reducing ERG proliferation via negatively controlling *tgfb3* expression in fibroblasts. Disruption of *plxnb1a/b* only partially replicated the *sema4ab* phenotype by also showing increased neuronal differentiation, but at the expense of ERGs. Interestingly, in mice, mutating Sema4A receptors *PlxnB1* and *PlxnB2* similarly decreased proliferative activity of progenitors, but increased their differentiation in the developing cortex, leading to a net effect of reduced neurogenesis [41]. Increased EdU labeling in ERGs after *sema4ab* disruption, may be attributable to increased levels of *tgfb3* expression, since we demonstrate here that the *tgfb3* promotes ERG proliferation (discussed below). A balance between the activation of Plexinb1 receptors and Tgfb3 levels might explain the overall effects of Sema4ab in attenuating both proliferation and differentiation in ERGs.

We here demonstrate that *sema4ab* signaling regulates expression of *tgfb3*, a crucial signaling molecule, in fibroblast-like cells *in vivo* [62]. We detect *tgfb3* expression only in fibroblasts by HCR-FISH and scRNA-seq, and *sema4ab* mostly in microglia. Hence, changes of *tgfb3* expression after *sema4ab* disruption are most likely a consequence of the perturbed cytokine environment. Our data indicate that fibroblast-produced Tgfb3 promotes regenerative neurogenesis. Exogenous TGFB3 has been shown to be a mitogen for retinal cells of the developing rat in vitro [84], such that Tgfb3 could act on progenitor cells directly in the lesioned spinal cord. However, fibroblast-derived Tgfb3 also mitigates inflammation in the lesioned spinal cord of larval zebrafish, indicating additional systemic functions of the cytokine [62]. In the adult zebrafish retina, Tgfb3 has been shown to increase pSmad levels in Müller glia, the retinal progenitor cells, and inhibit their injury-induced proliferation [76]. However, there is also evidence for pro-regenerative functions of Tgfb3 in zebrafish retina regeneration, perhaps through indirect effects [85–87]. This indicates context-dependent functions for Tgfb3 in neural development and regeneration.

In addition to signaling to other cell types, *sema4ab* may also maintain the activation state of microglia. KEGG analysis in microglia clusters highlighted reduced activity of important activation pathways, related to bacterial (Toll-like receptors), viral (RIG-I) response, and initiation of the inflammatory response [55–57] to be changed after disruption of *sema4ab*. In addition, the top down-regulated genes, including activation markers such as *grn1* [88], confirm this change in activation state. Finally, we found reduced expression of pro-inflammatory cytokines in microglia. Since the activation state of microglia affects cytokine signaling [89], it was not surprising to detect significant changes in the abundance of different cytokines, in particular a reduction of pro-inflammatory ones, in the absence of *sema4ab.* In mammals, microglia cells also express Sema4A [38,39], including in a spinal lesion site, as analyzed here. In agreement with our findings, injecting exogenous Sema4A into the mouse brain increased activation of microglia there [90]. Mammalian macrophages also increase *Sema4A* expression when activated by lipopolysaccharides [39].

Interestingly, we observed reduced expression of *tgfb1* when *sema4ab* was disrupted. Although Tgfb1 and Tgfb3 belong to the same ligand family, reports show that Tgfb1 and Tgfb3 have opposing roles in terms of scar resolution; Tgfb1 is widely described as pro-fibrotic, whereas Tgfb3 has been associated with scar resolution and scar-less healing in wound injury models [91]. Thus, antagonistic roles have been described. Therefore, the reduction of *tgfb1a* in our spinal cord injury together with the upregulation of *tgfb3* may contribute to a pro-regenerative shift in the lesion environment after *sema4ab* disruption.

How microglia signal to fibroblasts is still unclear. While we show that *sema4ab* and microglial cells are necessary to reduce *tgfb3* expression in fibroblasts, disruption of of potential receptors had no effect on *tgfb3* expression levels (*plxnb1a/b*) or regenerative neurogenesis (*plxnb2a/b*). Inhibiting Il-1beta or Tnf signaling also produced negative results. However, a number of other potential receptors for Sema4ab (*havcr1, havcr2, nrp1b, plxnd1*) [46,92] or for microglia-derived cytokines (e.g., *il6st* or *il11ra*) could also play a role in the complex signaling interactions of different cell types in the injury site.

Our re-analysis of mouse scRNA-seq data shows that microglia in a spinal injury site in mice selectively express *Sema4A,* spinal progenitors express *Plxnb1* and fibroblasts express *Tgfb3*. Hence, in principle, Sema4A could also contribute to inhibition of neurogenesis in the lesioned spinal cord of mice. A relatively stronger Sema4A signal in mice than in zebrafish could tip the balance to inhibition of neurogenesis after spinal injury. Selective expression of *Tgfb3* in fibroblasts indicates the possibility for similar suppression of *Tgfb3* in fibroblasts in an environment that is conditioned by Sema4A from microglia in a non-regenerating system. The interactions between fibroblasts and immune cells after CNS injury in mammals are highly dynamic, such that *in vivo* experiments would be needed to determine whether expression levels in mice are adequate to establish such interactions [35].

We show that *sema4ab* promotes axonal growth and swimming function recovery, while regenerative neurogenesis is reduced. This raises the possibility that enhancing neurogenesis beyond an optimal level may interfere with other regenerative outcomes, such as axon regrowth or functional recovery, perhaps through mal-adaptive integration into the repaired circuitry. For example, excessive neurogenesis in the hippocampus after stroke impairs memory, which in turn can be rescued by experimentally reducing levels of neurogenesis [23]. However, whether the observed effects on regenerative neurogenesis and axonal regrowth are connected is still unclear. Alternatively, *sema4ab* could act directly on axonal guidance, for instance via Sema4 receptors such as neuropilins [93], which can act as axon guidance receptors [94]. The altered cytokine environment is also likely to contribute to reduced axonal regrowth in the absence of *sema4ab* function*.* This could happen, for example, through modulation of the ECM [95,96].

### Study limitations

First, while the genes we manipulate (*sema4ab, plxnb1a/b,* tgfb3) show strong enrichment in the cell types of interest, we cannot exclude that our global perturbation approaches had inadvertent effects on other cell types with low expression levels. Second, direct signaling from microglia to fibroblasts cannot be comprehensively addressed, indicating a high degree of complexity of cell interactions within the injury environment. Third, although the pharmacological inhibition of *tgfb* signaling (SB431542) aligns with the results obtained using somatic mutants for *tgfb3*, the drug can affect multiple ALK receptors, and therefore the conclusions obtained from this experiment should be considered cautiously.

### Conclusions

We conclude that the microglia activation state is crucial for the outcome of a spinal injury. The balance of pro- and anti-regenerative signals from multiple cell types determines the regenerative outcome. Single-cell -omics approaches are well placed to unravel such complexity. Here, we identify *sema4ab,* predominantly expressed in microglia, as a potentially evolutionarily conserved signal in the regulation of the cytokine milieu and as an inhibitor of regeneration of neurons and axons. Hence, *Sema4a* may present a potential therapeutic target in non-regenerating mammals*.*

## Materials and methods

### Ethics statement

All experiments were performed under state of Saxony licenses TVV 36/2021, TVV 45/2018, and holding licenses DD24-5131/364/11, DD24-5131/364/12 (approved by Landesdirektion Sachsen, Referat 25 Veterinärwesen und Lebensmittelüberwachung); conformed to the guidelines established by the European Communities Council Directive of 22 September 2010 (2010/63/UE) and were in accordance with German animal law and regulations (Tierschutzgesetz TierSchG version 18 May 2006 with all modifications included thereafter until the date of publication of this manuscript).

### Animals

All zebrafish lines were raised and kept under standard conditions [97]. For experimental conditions, larvae up to an age of 5 days post-fertilization (dpf) were used of the following lines: wild type (AB); Tg(*mnx1:GFP*)^ml2tg^, abbreviated as *mnx1:GFP* [68]; Tg(*her4.1:EGFP*)^y83Tg^, abbreviated as *her4.1:EGFP*) [65]; Tg(*olig2:EGFP*), abbreviated as *olig2:EGFP* [98]; Tg(*olig2:dsred2*)^vu19^, abbreviated as *olig2:dsRed* [99]; Tg(*mpx:GFP*)^uwm1^, abbreviated as *mpx:GFP* [64]; Tg(*mpeg1.1:mCherry*)^gl23^, abbreviated as *mpeg1:mCherry* [63]; Tg(*pdgfrb:Gal4ff*)^ncv24^; Tg(*UAS:GFP*), abbreviated as *pdgfrb:GFP* [100]; TgBAC(*il1b:eGFP*)^sh445^, abbreviated as *il1b:EGFP* [101]; Tg(*tnfa:EGFP*)^ump5Tg^, abbreviated as *tnfa:EGFP* [102]; Tg(*elavl3:mrasKate2*)^mps1Tg^, abbreviated as *elavl3:mKate [37];* and *csf1ra*^*j4e1/j4e1*^ *× csf1rb*^*+/re01*^*,* abbreviated as *csf1ra/b −/−* [75].

### Spinal cord lesion

Lesions of larvae were performed as previously described [14]. Briefly, at 3 dpf, zebrafish larvae were deeply anaesthetized in E3 medium [103] containing 0,02% of MS-222 (Sigma). Larvae were then transferred to an agarose plate. After removal of excess water, larvae were placed in lateral positions. A transection of the entire spinal cord was made using a 30G syringe needle at the level of the 15th myotome, without injuring the notochord.

### Generation of somatic mutants

Somatic mutations were generated mainly following a previous protocol [48]. CRISPR gRNAs were selected using the CRISPRscan website (www.crisprscan.org/gene) webtools. gRNAs were chosen based on the CRISPRScan score (preferentially >60), and on the availability of a suitable restriction enzyme recognition site (www.snapgene.com) that would be destroyed by the gRNA. Additional criteria were to avoid the first exon and to target functional domains. 1 nL of gRNA mix (1 µl of Tracer (5nM, TRACRRNA05N, Sigma-Aldrich), 1 µl of gRNA (20µM, Sigma-Aldrich), 1 µL of phenol red (P0290, Sigma-Aldrich), and 1 µL of Cas9 (20µM, M0386M, New England Biolabs) was injected into one-cell stage embryos. gRNA efficiency was tested by restriction fragment length polymorphism analysis (RFLP) in a proportion of larvae for each experiment as an internal experimental control [48]. Sequences for gRNAs and primers for RFLPs can be found in S7 Table. All experiments conducted with somatic mutants were carried out with a control injected group with a non-targeting gRNA.

### Generation of s*ema4ab* germline mutant and *Tg(mfap4:mCherry-CAAX;myl7:mCerulean*) line

The *sema4ab* mutant stable line (*sema4ab*^tud123^) was generated by raising somatic mutants as potential founders, as described [48]. The founder selected to generate the stable line carried a 14 bp insertion in exon 6 that generated a frameshift with a premature stop codon. Sequencing primers were the same as for RFLP.

BDMs/microglia selectively express *mfap4* [104]*.* To generate the *Tg(mfap4:mCherry-CAAX;myl7:mCerulean)* line (abbreviated as *mfap4:mCherry)*, pDEST *mfap4:turquoise2* vector purchased from Addgene (#135218) was used to PCR-amplify the *mfap4* promoter sequence with primers designed to introduce 5′ SacI and 3′ AscI. In parallel, a second plasmid backbone containing *ins:nls-mCerulean; cryaa:mCherry* was digested with SacI/AscI and ligated with *mfap4* using SacI/AscI sites *(mfap4:nls-mCerulean; cryaa:mCherry).* Subsequently, mCherry was PCR amplified by using primers designed to introduce 5′ EcoRI and 3′ CAAX box followed by a stop codon and PacI. Then *mfap4:nls-mCerulean; cryaa:mCherry* were digested and ligated using EcoRI/PacI to mCherry-CAAX fragment *(mfap4:mCherry-CAAX; cryaa:mCherry).* Finally, the newly generated *mfap4:mCherry-CAAX; cryaa:mCherry* construct and *ins1.1:ins-sepHLourin; myl7:NLS-mCerulean* constructs were digested with EcoRI/PacI to yield compatible fragments which were ligated to generate the final construct *(mfap4:mCherry-CAAX;myl7:mCerulean).* The entire construct was flanked with I-Sce sites to facilitate transgenesis and was injected in wild type AB eggs. Several founders were selected based on mCerulean (blue) expression in the heart and mCherry (red) expression in BDMs and the founder with mendelian segregation in the F1 was selected. Primers are described in S8 Table.

### *In situ* hybridization chain reaction (HCR-FISH)

Larvae were collected in E3 medium and incubated with 0.003% 1-phenyl 2-thiourea (PTU) at 6 hpf to suppress melanocyte development. Larvae were fixed overnight in 4% paraformaldehyde (PFA) at 4 °C and then transferred to 100% Methanol (MeOH) at −20 °C overnight for permeabilization. The next day, larvae were rehydrated in a graded series of MeOH at room temperature (RT) and subsequent washes in phosphate-buffered saline with 0.1% Tween (PBST). Thereafter, larvae were treated with 500 μL proteinase K (Roche) at a concentration of 30 μg/mL for 45 min at RT, followed by post-fixation in 4% PFA for 20 min RT and washes with PBST (3 × 5 min). Pre-hybridization was performed using 500 μL of pre-warmed probe hybridization buffer (Molecular Instruments [105]) for 30 min at 37 °C. The probe sets, which were designed based on the NCBI sequence by Molecular Instruments [105], were prepared using 2 pmol of each probe set in 500 μL of pre-warmed hybridization buffer. Then, buffer was replaced with probe sets and incubated for 16 h at 37 °C. On the following day, samples were washed 4 × 15 min with pre-warmed probe washing buffer (Molecular Instruments) at 37 °C followed by 2 × 5 min in 5× sodium chloride sodium citrate with 0.1% Tween (SSCT) at RT. Larvae were then treated with 500 μL of room temperature equilibrated amplification buffer (Molecular Instruments, LA, USA) for 30 min at RT. Hairpin RNA preparation was performed following the manufacturer’s instructions. Samples were incubated with Hairpin RNAs for 16 h in the dark at RT. Finally, samples were washed for 3 x 30 min in 5 x SSCT at RT and immersed in 70% glycerol for imaging on a Zeiss LSM980 confocal microscope.

For HCR-FISH on adult animal, fish were killed, perfusion-fixed with 4% para-formaldehyde, and tissue slices of 50 µm thickness were obtained from dissected unlesioned spinal cord using a vibrating blade microtome (VT1200S, Leica, Wetzlar, Germany), as described [106]. HCR was performed right away following the protocol described above for larvae, with the exception that no permeabilization steps were applied (starting directly with hybridization blocking buffer step).

### Combining EdU with motor neuron and ERG labeling

Labeling newly generated motor neurons followed previous protocols [14]. Briefly, lesioned and unlesioned larvae were incubated in E3 medium containing 1% dimethylsulfoxide (DMSO, Sigma-Aldrich, Taufkirchen, Germany) and 1:100 EdU (Thermo-Fisher, Schwerte, Germany) for 48 h. Samples were fixed in 4% PFA for 3 h at RT and permeabilised in 100% MeoH at −20 °C for at least overnight. Larvae were then rehydrated, tails were dissected and incubated in proteinase K (Roche, Sigma-Aldrich) for 45 min at RT at a concentration of 10 μg/mL and then postfixed at RT in 4% PFA for 15 min. Then, larvae were incubated for 20 min at RT in 0.5% DMSO-PBST and incubated for 2 h at RT with the Click-it Chemistry Edu Alexa 647 kit (Thermo-Fisher, Waltham, USA) as described by the manufacturer. Subsequently, tails were washed in PBST and incubated with blocking buffer solution (2% BSA, 1% DMSO, 1% Triton, 10% Normal donkey serum, in PBST) for 1 h at RT and then with a chicken anti-GFP antibody, (1:200, Abcam, Cambridge, UK) at 4 °C for 72 h. Secondary antibody (alexa-488 anti-chicken, 1:200, Jackson Immuno) incubation took place overnight at 4 °C. Finally, samples were washed in PBST and transferred to 70% glycerol in PBST, mounted and imaged. Imaging was performed using a Widefield Axio Observer (Inverted) with ApoTome 2 Unit (Zeiss). Images were analyzed using Fiji [107,108]. Newly generated motor neurons were counted by assessing the number of double-positive cells for GFP and EdU. Cells were counted manually along the Z-stack, ensuring counting individual cells in single optical sections, in 50 µm windows located at both sides of the injury site (100 µm in total). To mitigate bias, experimental conditions were occluded for the analysis. At least two independent experiments were performed. Individual experiments were merged and analyzed for statistical significance.

To label proliferating ERGs, we followed the same experimental procedure, imaging and cell counting analysis as for newly generated motor neurons using the ERG reporter line *her4.1:EGFP* instead of the *mnx1:GFP* transgenic line.

### Immunofluorescence

Samples were fixed in 4% PFA at 24 hpl for 1.5 h at room temperature and permeabilised in 100% methanol at −20 °C for at least overnight. Larvae were then rehydrated, tails were dissected, and incubated for 10 min in 100% chilled acetone at −20 °C. Samples were washed and incubated with 500 μL proteinase K (Roche) at a concentration of 12.5 μg/mL for 45 min at room temperature, followed by post-fixation in 4% PFA for 20 min at room temperature and washes with PBST (3 × 5 min). Tails were then incubated with blocking buffer solution (2% BSA, 1% DMSO, 1% Triton, 10% Normal donkey serum, in PBST) for 1 h at room temperature and then with the corresponding antibody, (mouse anti-acetylated tubulin, 1:300, Sigma, T6793; rabbit anti-mCherry, 1:200, Invitrogen, PA5-34974; mouse anti-4C4, 1:50, European Collection of Authenticated Cell Cultures, 7.4.C4) at 4 °C for 72 h. After washings in PBST, incubation with matching secondary antibody (1:200, Jackson Immuno) took place overnight at 4 °C. Finally, samples were washed in PBST and transferred to 70% glycerol in PBST, mounted and imaged.

### Generation of heat shock plasmid and treatment

To create the pTol-hsp70l:sema4ab-T2a-mcherry plasmid, we modified the pTol-hsp70l:mCherry-T2A-atho1b vector [109]. We PCR-amplified the *sema4ab* coding sequence from genomic cDNA and PCR-amplified T2a-mcherry with specific primers (S9 Table). Then, we performed a simultaneous multiple fragment ligation in the opened backbone vector. The final construct pTol-hsp70l:sema4ab-T2a:mcherry was verified by sequencing, qRT-PCR for over-expression and mCherry fluorescence after injection.

### Measurement of axonal regrowth

As an indicator for successful axon regrowth, we determined the extent of continuous labeling of axons across the spinal cord injury site, termed axonal bridging. We determined the thickness of the axonal bridge at its narrowest point. Spinal lesion was performed at 3 dpf as described in larvae above. At 2 hpl, larvae were inspected under a fluorescence stereo-microscope and those larvae with incomplete spinal cord transection and those in which the notochord was inadvertently injured (over-lesioned) were excluded from the experiment. At 48 hpl, larvae were fixed and immuno-labeled against acetylated tubulin. Images were taken with a Dragonfly Spinning Disk microscope (Andor/Oxford Instruments, Belfast, UK) with a step size of 1 µm.

The bridge thickness was determined using a custom script in Fiji [107,108]. Measures were taken at the narrowest point of the axonal bridge, automatically detected by using a maximum projection of the z-stack and a binary segmentation. Measurements of three independent experiments were obtained and merged for statistical analysis.

### Analysis of swimming behavior

The swimming behavior was assessed using the ZebraBox (View Point, Lyon, France). As above, under- and over-lesioned larvae were excluded from the experiment at 2 hpl. Subsequently, the larvae were individually arranged in 24-well plates, ensuring representation of all groups on each plate, and kept in the incubator at 28 °C until 48 hpl. Larval movements were automatically tracked during three repetitions of the same protocol. The protocol comprised 1 min of rest, a 1-s vibration stimulus (frequency set at 1,000 Hz [target power 100]), followed by 59 s of no stimulus. The analysis focused on the time period of 1 min encompassing the vibration stimulus (including the 1-s stimulus and the subsequent 59 s). For each larva, the mean of their swimming distances over the three repeats was computed. Two independent experiments with an average of 30 larvae per condition were performed and the results were merged for statistical analysis (see below and figure legends).

### Quantitative RT-PCR

Following spinal cord lesion of 3 dpf, RNA extraction was performed at 1 dpl using the RNeasy Mini Kit (Qiagen) according to the manufacturer’s instructions (50 larvae/condition). The injury site was enriched by cutting away rostral and caudal parts from the larvae at positions approximately 3 somites away from the lesion site in either direction. cDNA was synthesized using the iScript cDNA Synthesis Kit (BioRad, cat #. 1708890) according to the manufacturer’s instructions. qRT-PCR was performed using the SsoAdvanced Universal SYBR Green Supermix kit (BioRad) according to the manufacturer’s instructions and run in triplicates on a LightCycler 480SW instrument (Roche). For gene-specific primers see S10 Table. For both control and experimental conditions, the average CP value from three technical replicates was first normalized to the average CP value of β-actin from the corresponding sample (ΔCP = CPgene − CPβ-actin). Subsequently, values were normalized to the control condition (ΔΔCP = ΔCPsample − ΔCPcontrol), and relative expression levels were calculated using the 2^ − ΔΔCP method [110,111]. This approach is performed for each biological replicate individually. This normalization strategy results in control samples always having a value of 1, while experimental samples are expressed relative to the control, thereby representing the fold-change values shown in the graphs. For statistical analysis, a One-Sample *T* test was used on 2^ − ΔΔCP values [112–114]. Raw data can be found in S5 and S6 Data. At least three biological repeats were performed for each gene, but further repeats were made occasionally as positive controls for effective lesion in subsequent experiments and pooled for statistical analysis to improve robustness.

### Immune cell counting in whole-mounted larvae

Larvae were lesioned at 3 dpf and fixed for 1 h in 4% PFA at different time points. Larvae were washed in PBST and transferred to glycerol (70% in PBST), mounted and imaged immediately using a Widefield Axio Observer (Inverted) with an ApoTome 2 Unit. Images were analyzed using Fiji with the experimental condition unknown to the observer. A window of 200 µm width and 75 µm height was placed in the center of the injury site of individual images, right above the notochord. Cells were counted manually inside the window along the Z-stack with the experimental condition occluded. At least two individual experiments were carried out and merged.

### Acridine orange labeling

Larvae of the *mpeg1:mCherry* line were incubated after a lesion in 1 µg/ml of Acridine Orange (stock solution 10 mg/ml in water) in E3 for 24 h. Then, fish were washed for 3 × 10 min in E3. Larvae were then mounted in 0.5% agarose in E3 for live imaging on the Dragonfly Spinning Disk microscope (Andor). To estimate total debris, GFP mean intensity was analyzed using a 250 × 100 µm counting window placed in the center of the injury site. To determine the number of BDMs/microglia that ingested debris, cells positive for both mCherry and GFP were counted in the injury site using same window as before.

### Fluorescence intensity/area measurements

Images were analyzed with Fiji ImageJ (https://imagej.net/ij/). The region of interest was defined as a rectangle of 400 × 100 µm, centered on the injury site and located right above the notochord. Z-stacks were collapsed in sum intensity projections and then background subtraction was performed before analyzing the mean intensity with a custom Fiji macro to automatize the process [107,108]. Mean intensity was represented by normalizing each value to the control average value.

### Drug treatment of larvae

SB431542 (Fisher Scientific) or YVAD (Sigma-Aldrich, Cat# SML0429) was dissolved in DMSO to a stock concentration of 10 mM. The working concentration was 50 µM prepared by a dilution of the stock solution in E3. Larvae were pre-treated for 2 h before injury and then incubated for 48 h for SB431542 or pre-treated for 4 h before injury and then incubated for 24 h for YVAD. Control larvae were treated with DMSO at the same concentration.

### Cell dissociation and fluorescence-activated sorting

Fifty larvae per sample were used. Lesion-site tissue was enriched by dissecting 3 somites rostral and caudal of the injury site and collecting them in cold 1× PBS. The tissue was dissociated using the Miltenyi Neural Tissue Dissociation Kit (P) with addition of collagenase. In brief, we removed excess 1x PBS and added 950 µL Buffer X. 6 µL of 1/1000 diluted β-mercaptoethanol, 50 µL Enzyme P and 8 µL collagenase (50 mg/mL) were added. The tissue was pipetted 10 times with 1 mL pipette tips and let it rotate at RT (~22–23 °C) for approximately 1 min. In the meantime, 10 µL of Buffer Y and 10 µL of Enzyme A were mixed and added this to the dissociation mix. Tissue was triturated slowly using fire-polished small-orifice glass Pasteur pipette. Each trituration was performed 10 times (6 s each) for 1 min, and we let the sample rotate for 1 min. This step was repeated until complete dissociation was achieved. The reaction was stopped after ~14 min by adding 1 mL of DMEM + FBS (1%) + Protease Inhibitor (1×) mix (DFP), followed by centrifugation at + 4 °C at 500 *g* for 5 min. The supernatant was removed and the pellet was resuspended in 1 mL of HBSS (Mg2+/Ca+) + Protease Inhibitor (1×) and then passed through a 40 µm filter equilibrated with 200 µL of DFP. Cells were recentrifuged at +4 °C at 500 *g* for 5 min. The pellet was resuspended in 300 µL of DFP. Just before cell sorting, 0.5 µL of propidium iodide (100 µg/mL) was added and mixed well by pipetting. Viable, propidium iodide-negative cells were sorted using FACS Aria (BD Biosciences) using a 100 µm nozzle at flow rate 1. 100,000 cells per sample were sorted into BSA-coated 1.5 mL Eppendorf tubes with 500 µL of a 0.4% BSA solution in 1x PBS. The sorted cells volume was around 200 µL and the final concentration of BSA was 0.28%. One control gRNA and two *sema4ab* gRNA samples were processed by the Dresden Concept Genome Center for cell encapsulation using the 10X Genomic V3.

### Droplet encapsulation and single-cell RNA sequencing

Cells were pelleted at 500 *g* for 5 min at 4 °C, leaving 20 µL of suspension. Cell viability was assessed with trypan blue, and samples with >80% viability were prepared for encapsulation. Single-cell RNA sequencing followed the 10x Genomics (Chromium Single-cell Next GEM 3′ Library Kit V3.1) workflow [115]. In brief, cells were mixed with Reverse Transcription Reagent and loaded onto a Chromium Single Cell G Chip, targeting 10,000 cells per sample. GEM generation, barcoding, reverse transcription, cDNA amplification, fragmentation, and purification were performed according to the manufacturer’s protocol. Libraries were prepared using 25% of cDNA, quantified, and sequenced on an Illumina NovaSeq 6000 in 100 bp paired-end mode.

### Alignment to zebrafish genome

The raw sequencing data were processed with the ‘count’ command of the Cell Ranger software (v7.1) provided by 10x Genomics. To build the reference, the zebrafish genome (GRCz11) and gene annotation (Ensembl 109), were downloaded from Ensembl (https://www.ensembl.org/). The ‘count’ command was used to generate count matrices that were further used by Seurat V5 for downstream analyses.

### Quality control and doublet removal

For data analyses, cells were kept based on the following conditions: (i) nFeature_RNA ≥ 200 (number of genes detected per cell), 500 < nCount_RNA < 50,000 (number of total reads detected per cell), (ii) percent.mt < 20% (mitochondrial RNA percentage), (iii) nCount_RNA/nFeature_RNA > 20-fold (an indication of possible doublets), (iv) genes found in fewer than 3 cells were removed from the analyses. Additionally, doublets were determined using scDblFinder V1.18.0 [116]. However, rare cells expressing markers for cell types distinct from the clusters they were allocated to remained and were possible doublets. Cells were removed, if cells other than neurons expressed *elavl3* or *elavl4*, if cells other than red blood cells expressed *hbbe1.3/hbbe2*, if cells other than muscle, fibroblast-like cells or keratinocytes expressed *col12a1a*, *col12a1b*, or if cells other than muscle or fibroblast-like cells expressed *fgl1,* or *vwde*. Using this method an additional ~5% of cells per sample were removed.

### Seurat data analyses

After removing low-quality cells and doublets, we utilized Seurat V5 for data integration using CCAIntegration method and followed its standard pipeline. Briefly, a Seurat object was created and the data were normalized. The top 2,000 variable genes were identified, and the data were scaled using all genes, with nCount_RNA regressed out. Clustering was performed at resolution 1, yielding 44 distinct clusters (numbered as 0–43).

### Finding marker genes and annotation of clusters

To identify marker genes (the genes which are specific to a cluster compared to the remaining all other cell types), the FindAllMarkers function from Seurat, using the default options was used. The “Wilcoxon rank sum test”, and *p*_val_adj (*p*-value adjusted) < 0.1 and avg_log2FC ≥ 0.25 (average log2 fold change) was used for selecting significantly expressed marker genes. Additionally, genes were selected using pct.1 (percentage in the cluster) > 0.2 and ordered, based on their avg_log2FC in descending order for each cell cluster. Based on the literature, known cell type marker genes were selected from the top ranked genes and used to annotate the main cell types accordingly.

The main cell types were annotated using the following marker genes and the top-ranked genes for each cluster [42,117,118]: Ependymoradial Glia (ERG; *fabp7a, sox2*), Enteric Nervous System (ENS; phox2aa, phox2b), Fibroblast-Like Cells (FBL; 3 main clusters, *col12a1a*, *col12a1b*, *pdgfrb*+), Keratinocytes (KC; *pfn1, krt4*), Peridermis (PD; *krt5, krt91, icn2)*, Muscle Cells (MC; m*eox1, mylz3*), Xanthophores (XP; *gch2, aox5)*, Oligodendrocyte (OD; *cldnk, mbpb, mpz*), Kolmer-Agduhr Cells (KA; *npcc, sst1.1*), neutrophils (NP; *lcp1, mpx, lyz*), BDMs/microglia (MM; *lcp1, mfap4, mpeg1.1*), Neurons (NN; *elavl3, elavl4*), Endothelia Cells (EC; *kdrl, cldn5b*), Notochord (NC; s*hha, gas2b*), Red Blood Cells (RBC; *hbbe1.3, hbbe2*) and 5 clusters that remained not annotated (c#28, c#31, c#32, c#34 and c#36).

Fibroblasts cluster were annotated using 10 top-ranked genes for each cluster (See gene list and references in S11A–S11C Fig and S4 Data). Tentative names were assigned when at least 3 genes out of 10 had a shared function that differentiate the cluster from the rest.

### Differentially expressed gene (DEG) analysis

The FindMarkers function in Seurat was used to determine the differentially expressed genes using “Wilcoxon rank sum test”. *p*_val_adj < 0.1 and abs(avg log2FC) ≥ 0.25 were used for selecting significantly expressed genes.

### Gene ontology term and KEGG analyses

For KEGG (Kyoto Encyclopedia of Genes and Genomes) analyses, GOstats [54], giving all differentially expressed genes (over- or under-represented) using 0.25 log2FC and *p*_value < 0.05 as input, were used. Enriched terms were selected with a *p*-value < 0.05.

### Cell–cell interaction

CellChat V2.1.0 [53] was used for cell-cell interaction and a custom cell-cell interaction as described [119]. In brief, the CellChat Database was modified by adding *sema4ab* as ligand and *plxnb1a, plxnb1b, plxnb2a, plxnb2b, plxnd1, havcr1, havcr2, nrp1a and nrp1b* as its receptors. As there was a pathway named SEMA4, the pathway was renamed to “SEMA4AB” for *sema4ab* and to “SEMA4other” for *sema4c/sema4g*. Then, CellChat objects were created for each data set using normalized data from the integrated Seurat object. Finally, the CellChat objects were merged for comparative analyses.

### Cell cycle scoring analysis

Cell cycle scoring analysis was performed using the “Cell-Cycling Scoring” code for Seurat in R [52] based in a published gene list [120] (loaded in Seurat) and adapted to zebrafish orthologues manually using ZFIN [121].

### Time-lapse microscopy

Zebrafish larvae were prepared for time lapse microscopy based on our established protocol (https://ibidi.com/img/cms/support/UP/UP12_Zebrafish_Co_Culture.pdf). Briefly, double transgenic zebrafish larvae (*pdgfrb:GFP*; *mfap4:mCherry*) were lesioned at 3 dpf and anaesthetized with MS-222 (160 mg/L in E3 medium). Low-melting-point agarose (0.5%, Biozym Plaque Agarose—Low melting Agarose #840100) was prepared in E3 medium and allowed to cool down to 38 °C before adding MS-222 (final concentration 80 mg/L). Anaesthetized zebrafish larvae were transferred to the agarose and one by one positioned into individual wells of the chambered coverslip. Six larvae were used in total; three control gRNA-injected and three *sema4ab* haCR-injected ones. Microscopy was performed with a Dragonfly Spinning Disk microscope (Andor/Oxford Instruments, Belfast, UK) with a temperature-controlled stage at 28 °C using a 20× objective (20×/0.75 U Plan SApo, Air, DIC, OLYMPUS, Tokyo, Japan). The GFP fluorophore was excited with a 488 nm laser (laser power 20%) with an exposure time of 180 ms and the mCherry flourophore was excited with a 561 nm laser (laser power 50%) with an exposure time of 900 ms. We acquired z-stacks of 43 optical sections, with a step size of 1 µm. The interval between single frames of the time-lapse movies was 6 min. Time-lapse was performed from 10 hpl until 25 hpl.

### Bias mitigation and statistical analysis

The experimental conditions were occluded before manual counting and analysis and processing was automated where possible (e.g., measurements of axonal regrowth and intensity measurements). For manual counts of EdU+ cells, pilot experiments were performed by two independent experimenters, both showing comparable effect sizes. Experiments were performed in duplicate, if not indicated differently.

Statistical methods to analyze scRNA-seq data are described above. For all other experiments, quantitative data was tested for normality using Shapiro–Wilk’s *W* test. Then, parametric (unpaired Student *t* test or one-way ANOVA) and non-parametric (Mann–Whitney *U* test or Kruskal–Wallis test) were applied as appropriate. Error bars in figures indicate standard error of the mean (SEM), *p*-values were represented as asterisks in the figures (*p < 0.05; **p < 0.01; *** *p* < 0.001) and exact p-values are given in figure legends. To generate graphs and for statistical analysis we used GraphPad Prism 11 (GraphPad Software, Boston, USA).

### Figure preparation

Images were adjusted for brightness, contrast and intensity. Drawings were made using CorelDraw X8 (Corel/Alludo, Ottawa, Canada). Figure plates were prepared using CorelDraw X8 (Corel/Alludo, Ottawa, Canada) and Adobe Photoshop 2024 (Adobe, CA, USA).

## Supporting information

S1 Fig Potential interaction between *sema4ab* in microglia and plexin receptors in ERGs revealed in cell type-enriched scRNA-seq datasets. (A) UMAP showing clusters in an mpeg1.1:GFP-enriched scRNA-seq. **(B)** UMAPs comparing unlesioned and lesioned conditions are shown. Note an increase of the microglia cluster after injury (circle). **(C,D)** Feature plots comparing expression of *apoeb* (C) and *sema4ab* (D) between unlesioned and lesioned larvae, indicating increased presence of both after injury (circled). **(E)** Dot plot showing all semaphorins expressed in the scRNA-seq dataset before and after lesion. Note that only *sema4ab* is substantially expressed and also upregulated after injury (rectangle). **(F**) UMAP showing clusters in a *her4.1:EGFP*-enriched scRNA-seq. Circle indicates ERGs. **(G)** UMAPs indicate presence of ERGs in unlesioned and lesioned conditions. **(H–J)** Feature plots indicating expression of *plxnb1a* (H) and *plxnb1b* (I) in ERGs of unlesioned and lesioned larvae (circled). Plotting only cells co-expressing receptors shows strong enrichment in ERGs (J, circled). Data are described in Cavone et al., Dev Cell 2021 Jun 7;56(11):1617–1630.e6.
(TIF)

S2 Fig Cell representation and potential interactions in scRNA-seq after *sema4ab* disruption. **(A)** Principal component analysis (PC1 and PC2) shows that gSema4ab replicates (R1 and R2) are more closely related to each other than to gControl. **(B)** A graph indicates similar percentages of cells per cluster across scRNA-seq samples. **(C)** A dot plot illustrates the communication probabilities of all ligand-receptor interactions identified by CellChat between microglia clusters MM#1 and MM#2 and fibroblast clusters. No dot indicates no interaction detected.
(TIF)

S3 Fig HCR-FISH method control and detection of *sema4ab* expression in neutrophils. **(A)** HCR-FISH for the negative control gene (*Drosophila* BicoidXA) shows non-specific labeling in some blood vessels, but not in the spinal cord in uninjured and injured larvae. **(B)** Multiplexed HCR-FISH at 6 and 24 hpl shows that some *sema4ab+* cells express *mpx:GFP* (white arrows: double-positive cells; green arrows: *mpx:GFP+* only; magenta arrows: *sema4ab*+ only). Scale bars: 100 µm (A, B), 10 µm (inset). Data files for graphs available in S3 Data.
(TIF)

S4 Fig Efficient disruption of *sema4ab* does not lead to large changes of larval growth. **(A)** RFLP and qPCR showing gRNA injection efficiency for *sema4ab*. For RFLP each lane represents one larva with and without digestion with the indicated restriction enzymes and with and without targeting these sites with gRNAs as indicated. Targeting the recognition sites with haCR gRNAs leads to efficient somatic mutation, as indicated by the almost complete resistance to digestion. Note a **~**50% reduction in mRNA abundance detected by qRT-PCR. **(B)** A schematic of the *sema4ab* gene structure and position of the mutation in the *sema4ab* germline mutant is shown. Note the insertion of 14 bp in exon 6 (sequence underlaid in red) and the frameshift that predicts a stop codon (red frame and asterisk). **(C)** A schematic indicating the experimental design for D-H is shown. **(D)** Photomicrographs of gControl and gSema4ab larvae are shown at 5 dpf. **(D–H)** Quantifications show no differences in body length (E: gControl: 3,836 µm ± 15.65; gSema4ab: 3,838 µm ± 15.72; Unpaired *t* test: *p* = 0.9917), notochord thickness (F: gControl: 92.09 µm ± 1.527; gSema4ab: 92.20 µm ± 0.9314; Mann–Whitney *U* test: *p* = 0.3423) or spinal cord thickness (G: gControl: 62.49 µm ± 2.215; gSema4ab: 57.83 µm ± 1.677; Mann–Whitney *U* test: *p* = 0.1134) between gControl and gSema4ab larvae. Eye diameter is slightly reduced in gSema4ab larvae (H: gControl: 378.2 µm ± 3.320; gSema4ab: 349.3 µm ± 2.784; Unpaired *t* test: *p* < 0.0001). **(I)** A schematic indicating the experimental design for I-K is shown. **(J)** Photomicrographs show wild type and germline mutant larvae for *sema4ab* at 5 dpf. **(K)** No difference was observed in body length of wild type and mutant larvae (wild type: 3,941 µm ± 15.95; *sema4ab −/−*: 3,940 µm ± 17.50; *t* test: Unpaired *t* test: *p* = 0.6855). **(L)** Eye diameter of mutants is slightly decreased (wild type: 383.3 µm ± 3.788; *sema4ab −/−*: 349.9 µm ± 2.56; Unpaired *t* test: *p* < 0.0001). Each dot for qRT-PCR represents a pool of 50 larvae. β-actin was used as housekeeping gene. Raw data for qRT-PCR can be found in S5 and S6 Data. Error bars show SEM. Scale bars = 1 mm. Data files for graphs available in S3 Data. Original gels with no adjustments can be found in S1 Raw Images.
(TIF)

S5 Fig Efficient heat-shock overexpression of *sema4ab* does not alter regenerative neurogenesis in lesioned wild-type larvae. **(A)** A map of the heat-shock vector injected to over-express *sema4ab* and a reporter (*mCherry*) is shown. **(B)** Heat-shock leads to detectability of mCherry protein mainly in muscle cells surrounding the injury site (position of ventral spinal cord indicated by *mnx1:GFP* transgene) at 24 hpl. **(C, D)** Experimental timeline to assess *sema4ab* over-expression by qRT-PCR after a single heat shock is shown in (C). qRT-PCR analysis shows that a single heat-shock leads to strongly increased *sema4ab* expression for at least 16 hours post heat-shock (hpHS, D). **(E)** Over-expression of *sema4ab* does not elicit any changes in the number of newly generated neurons after spinal lesion (F; gControl: 8.8 cells per larva ± 0.98; hsp70:sema4ab: 6.4 cells per larva ± 0.86; Mann–Whitney *U* test: *p* = 0.3850). Each dot for qRT-PCR represents a pool of 50 larvae. β-actin was used as housekeeping gene. Raw data for qRT-PCR can be found in S5 and S6 Data. Error bars show SEM. Dotted lines show the injury site in B and E. Scale bars: 50 µm. Data files for graphs available in S3 Data.
(TIF)

S6 Fig Somatic mutations of *plxnb2a and plxnb2b* do not affect regenerative neurogenesis. **(A)** RFLP and qPCR showing gRNA injection efficiency for *plxnb1a* and *plxnb1b*. For RFLP each lane represents one larva with and without digestion with the indicated restriction enzymes and with and without targeting these sites with gRNAs as indicated. Targeting the recognition sites with haCR gRNAs leads to efficient somatic mutation, as indicated by the almost complete resistance to digestion. Note a **~**50% reduction in the RNA detected by qRT-PCR for either receptor. **(B, C)** HCR-FISH shows expression of *plxnb2a* (B) and *plxnb2b* (C) in a narrow domain in the spinal cord that does not change after lesion. Additional signal can be observed in the injury site after lesion for both genes (asterisks). **(D, E)** High magnifications of HCR-FISH for *plxnb2a* and *plxnb2b* show no detectable expression of *plxnb2a* at the level of ERGs (D), but of *plxnb2b* in *her4.1:EGFP*+ cells (E). **(F)** RFLP showing high gRNA injection efficiency for *plxnb2a* and *plxnb2b*. Each lane represents one larva with and without digestion with the indicated restriction enzymes and with and without targeting these sites with gRNAs as indicated. Targeting the recognition sites with haCR gRNAs leads to efficient somatic mutation, as indicated by the almost complete resistance to digestion. **(G, H)** haCR gene disruptions of *plxnb2a* (G, gControl: 5.6 cells per larva ± 1.26; gPlxnb2a: 5.5 cells per larva ± 1.00; Mann–Whitney *U* test: *p* = 0.6938) or *plxnb2b* (H, gControl: 6.9 cells per larva ± 0.83; gPlxnb2b: 5.9 cells per larva ± 0.73; Mann–Whitney *U* test: *p* = 0.3336) do not lead to changes in the number of newly generated motor neurons, compared to lesioned gControl-injected larvae. Each dot for qRT-PCR represents a pool of 50 larvae. β-actin was used as housekeeping gene. Raw data for qRT-PCR can be found in S5 and S6 Data. Error bars show SEM. Dotted lines delineate the position of the spinal cord. Scale bars: 100 µm (B, C), 25 µm (D, E). Data files for graphs available in S3 Data. Original gels with no adjustments can be found in S1 Raw Images.
(TIF)

S7 Fig Cell recruitment and phagocytosis in the injury site are mostly comparable after *sema4ab* ablation. **(A)** A schematic indicating the experimental design for B–E is shown. **(B, C)** Neutrophil numbers do not show differences at 4 hpl (B, gControl: 45.89 cells per larva ± 2.1; gSema4ab: 42.69 cells per larva ± 1.8; Mann–Whitney *U* test: *p* = 0.1854) and 24 hpl (C, gControl: 14.63 cells per larva ± 1.7; gSema4ab: 16.33 cells per larva ± 2.5; Mann–Whitney *U* test: *p* = 0.5005) in *sema4ab* somatic mutants compared to control gRNA-injected larvae. **(D)** The abundance of fibroblast-like cells, measured by GFP-positive volume, shows no differences between controls and somatic mutants for *sema4ab* at 24 hpl (gControl: 135,342 µm^3^ ± 17,127; gSema4ab: 118,911 µm^3^ ± 16,774; Mann–Whitney *U* test: *p* = 0.6842). **(E)** There is a reduction in the number of BDMs/microglia in gSema4ab compared to gControl larvae larvae at 24 hpl (gControl: 83.03 cells per larva ± 9.3; gSema4ab: 73.73 cells per larva ± 3.7; Mann–Whitney *U* test: *p* = 0.0156). **(F)** Cell death, measured by average labeling intensity of acridine orange in the lesion site, shows no differences between experimental groups (gControl: 56.67 ± 7.5; gSema4ab 54.27 ± 5.0; Mann–Whitney *U* test: *p* = 0.8123). **(G)** BDMs/microglia phagocytosis, evaluated by counting *mpeg1:mCherry+* cells with engulfed acridine orange+ particles, shows no differences between groups (gControl: 22.21 cells per larva ± 4.327; gSema4ab 17.23 cells per larva ± 1.912; Mann–Whitney *U* test: *p* = 0.9714). White boxes designate quantification windows. Dotted lines outline the edges of the injury site. Error bars show SEM. Scale bars: 50 µm. Data files for graphs available in S3 Data.
(TIF)

S8 Fig Loss of *sema4ab* reduces *il1b* and *tnfa* expression *in vivo, but plxnb1a/plxnb1b* disruption does not alter the cytokine profile of the lesion site. **(A)** A schematic indicating the experimental design for cytokine quantification *in situ* is shown. **(B)** *il1b:GFP* fluorescence intensity reported in transgenic fish is decreased in lesioned *sema4ab* somatic mutants compared to control gRNA-injected fish at 24 hpl (−1.45 fold-change; Unpaired *t* test: *p* = 0.0004). **(C)** *tnfa:EGFP* fluorescence intensity reported in transgenic animals is decreased in lesioned *sema4ab* somatic mutants compared to control injected fish at 24 hpl (−1.49 fold-change; Unpaired *t* test: *p* < 0.0001). **(D)** *il1b* expression in microglia (*apoeb+* cells) detected by HCR shows a reduction of 11% in mean intensity (Unpaired *t t*est: *p* = 0.0380). **(E)** A schematic indicating the experimental design for qRT-PCR. **(F)** qRT-PCR analyses of major pro-inflammatory cytokines (*il1b, tnfa, il6*) and *tgfb3* in *plxnb1a/plxnb1b* double somatic mutants show no differences to injured control gRNA-injected larvae at 24 hpl (One-sample t *t*est; *il1b*: *p* = 0.537; *tnfa*: p = 0.4840; *il6*: p = 0.5464; *tgfb3*: p = 0.687). Each dot for qRT-PCR represents a pool of 50 larvae. β-actin was used as housekeeping gene. Raw data for qRT-PCR can be found in S5 and S6 Data. Error bars show SEM. Scale bars: 50 µm. Data files for graphs available in S3 Data.
(TIF)

S9 Fig Experimental manipulations and cell interactions in the lesion. **(A)** HCR-FISH for *tgfb3* shows an increase in fluorescence intensity in lesioned *sema4ab* somatic mutants compared to lesioned control gRNA-injected fish at 24 hpl (gControl: 1.0 ± 0.058; gSema4ab: 1.3 ± 0.046; Unpaired *t* test: *p* = 0.0011). Dotted lines show the injury site. **(B)** HCR-FISH at 24 hpl shows *tgfb3* expression in fibroblast-like cells (*pdgfrb:GFP*^+^; arrowheads). The mean intensity of the *tgfb3* signal in *pdgfrb:GFP*^+^ cells shows an increase in *sema4ab* haCR-injected larvae at 24 hpl (+1.22-fold change, Unpaired *t* test p = 0.0322). **(C)** Individual frames of time-lapse movies show *mfap4:mCherry*+ cells in close contact to *pdgfrb:GFP*+ cells in both control gRNA-injected animals and in *sema4ab* somatic mutants. **(D)** Time series of movie frames show that *mfap4:mCherry+* cells (arrowheads) migrate along fibroblast processes in both conditions**. (E)** RFLP and qPCR showing gRNA injection efficiency for *tgfb3*. For RFLP each lane represents one larva with and without digestion with the indicated restriction enzymes and with and without targeting these sites with gRNAs as indicated. Targeting the recognition sites with haCR gRNAs leads to efficient somatic mutation, as indicated by the almost complete resistance to digestion. Note a **~** 50% decay in the RNA detected by qPCR. Each dot for qRT-PCR represents a pool of 50 larvae. β-actin was used as housekeeping gene. Raw data for qRT-PCR can be found in S5 and S6 Data. Scale bars: 100 µm (A, C,), 50 µm (B) 15 µm (D). Data files for graphs available in S3 Data. Original gels with no adjustments can be found in S1 Raw Images.
(TIF)

S10 Fig Microglia depletion increases *tgfb3* levels while cytokine/cytokine receptor loss of function drives no changes. **(A–C)** A Microglia depletion line shows an increase in *tgfb3* levels at 24 hpl by qRT-PCR (One-sample *t* test: *p* = 0.0281). **(D, E)** Impairing cytokine signaling by YVAD (impairs il1b cleavage) and *tnfrsf1a* loss of function, results in no meaningful changes in tgfb3 levels at 24 hpl by qRT-PCR (One-sample *t* test; YVAD: *p* = 0.2303; gTnfrsf1a: *p* = 0.6461). **(F)** Feature plots showing main pro-inflammatory cytokine receptor expression pattern. Note a strong expression in fibroblasts (circle). Each dot for qRT-PCR represents a pool of 50 larvae. β-actin was used as housekeeping gene. Raw data for qRT-PCR can be found in S5 and S6 Data. Scale bars: 50 µm. Data files for graphs available in S3 Data.
(TIF)

S11 Fig *tgfb3* is expressed in subsets of fibroblasts and in close contact with ERGs and growing axons. **(A)** UMAP of fibroblasts subset showing 7 clusters of cells. **(B)** Top 10-ranked gene analysis showing differential expressing genes among clusters. **(C)** UMAP showing genes used for annotation and tentative cluster names. **(D)** Feature plot showing expression pattern of *tgfb3*. Arrows point enriched clusters for the gene**. (E)** Single-Stacks of the lesion site at the level of the spinal cord showing *tgfb3*-expressing fibroblasts (arrow). **(F)** Live imaging showing that fibroblasts (green, yellow arrows) are in the vicinity of motor neuron progenitors (magenta, cyan arrows). **(G)** Live imaging showing that fibroblasts (green, yellow arrows) are in close contact with growing axons (magenta, cyan arrows). Yellow dotted lines mark the spinal cord. Scale bars: 50 µm.
(TIF)

S12 Fig scRNA-seq shows that genes analyzed in this study are also expressed in a spinal injury site in adult zebrafish. **(A)** UMAP of the main cell types in the lesion site of adult zebrafish from Cigliola et al. 2023 *Nat Commun* 14:4857 is shown. **(B)** Feature plots show the expression of reference genes for relevant cell types (microglia: *p2ry12*; ERGs: *her4.1*; and fibroblasts-like cells: *pdgfrb*), as well as the expression of genes of interest for this study, *sema4ab, plxnb1a, plxnb1b* and *tgfb3*. Circles label expression in cell types that is conserved between larvae and adults. **(C)** HCR-FISH in cross sections of unlesioned spinal cords of 4-month-old adult zebrafish show expression of *plxnb1a and plxnb1b* in the ventricular zone*.* Dorsal is up and ventral is down. Dotted circle shows the central canal. Scale bar: 50 µm.
(TIF)

S13 Fig scRNA-seq shows that genes analyzed in this study are also expressed in a spinal injury site in adult mice. **(A)** A UMAP representation indicating the main cell types in a mouse spinal lesion from Xue et al., 2024 *Nat Commun* 15:6321 is shown. **(B)** Expression pattern of mouse ortholog genes *Sema4A*, *Tgfb3* and *Plxnb1* is conserved in microglia (*P2ry12*), fibroblasts (*Pdgfra*) and ependymal cells (*Foxj1*), respectively. Circles label expression in cell types that is conserved between zebrafish and mouse.
(TIF)

S1 Table Cell yields of scRNA-seq experiments. (DOCX)

S2 Table The mean, median, and total number of reads (nCount_RNA) and unique genes (nFeatures_RNA) detected per sample. (DOCX)

S3 Table The number of reads mapped to the *sema4ab* gene and the number of reads mapped to the *sema4ab* haCR-targeted site. (DOCX)

S4 Table Examples of reads that span the haCR target site in the *sema4ab* haCR-injected samples in scRNA-seq experiments. (DOCX)

S5 Table Cell cluster annotation reference. The table shows the correspondence between each cluster obtained by unsupervised clustering and the correspondent main cell type and cluster name assigned.
(DOCX)

S6 Table Top 10 downregulated genes in MM#1 after *sema4ab* somatic mutation in scRNA-seq. (DOCX)

S7 Table Table showing the gene sequences targeted with gRNAs. Each column represents the targeted sequence of a gene and the primers and restriction enzymes used to validate the efficiency of the mutation.
(DOCX)

S8 Table Primers used for cloning the *mfap4:mCherry-CAAX;myl7:mCerulean* vector. (DOCX)

S9 Table Primers used for cloning the *hsp70l:sema4ab-t2a-mCherry* vector. (DOCX)

S10 Table List of qRT-PCR primers. (DOCX)

S1 Data Changes in transcript abundance for macrophage activation pathways detected by KEGG analysis. Note that most genes are down-regulated.
(XLSX)

S2 Data Individual data points for all the experiments shown in the Main Figures. (XLSX)

S3 Data Individual data points for all the experiments shown in the Supplementary Figures. (XLSX)

S4 Data Citation list for genes used for fibroblast cluster annotation. (XLSX)

S5 Data qRT-PCR raw data for Main Figures including all values used in the qRT-PCR analysis (CP, average CP, ΔCP, ΔΔCP, and 2^ − ΔΔCP). Roman numbers indicate individual experiments. (XLSX)

S6 Data qRT-PCR raw data for Supplementary Figures including all values used in the qRT-PCR analysis (CP, average CP, ΔCP, ΔΔCP, and 2^ − ΔΔCP), as well as a calculation template. Roman numbers indicate individual experiments. (XLSX)

S1 Movie lesioned control-gRNA injected. 10 to 25 hpl (6 min intervals).
(MP4)

S2 Movie lesioned *sema4ab* haCR-injected. 10 to 25 hpl (6 min intervals).
(MP4)

S1 Raw Images Original gels with no adjustments. Gels were acquired using the Axygen Gel Documentation System (Corning, NY, USA) and imaged from the pocket to the electrophoresis front. No pockets or lane were excluded from the image.
(TIF)

## Abbreviations

BDMs: blood-derived macrophages
CNS: central nervous system
DEG: differentially expressed gene
DMSO: dimethylsulfoxide
dpf: days post-fertilization
ERGs: ependymo-radial glial cells
haCRs: highly-active CrRNAs
HCR-FISH: hybridization chain reaction fluorescence *in situ* hybridization
hpl: hours post-lesion
PBST: phosphate-buffered saline with 0.1% Tween
PFA: paraformaldehyde
PTU: 1-phenyl 2-thiourea
RFLP: restriction fragment length polymorphism analysis
RT: room temperature
Sema4A: Semaphorin 4A
