# Supplementary figures and images for: The microglia-derived protein Sema4ab attenuates regenerative neurogenesis after spinal cord injury in zebrafish
Source: PLoS Biol. 2026 Jun 18;24(6):e3003865. doi: 10.1371/journal.pbio.3003865 (PMC13309017; doi:10.1371/journal.pbio.3003865)

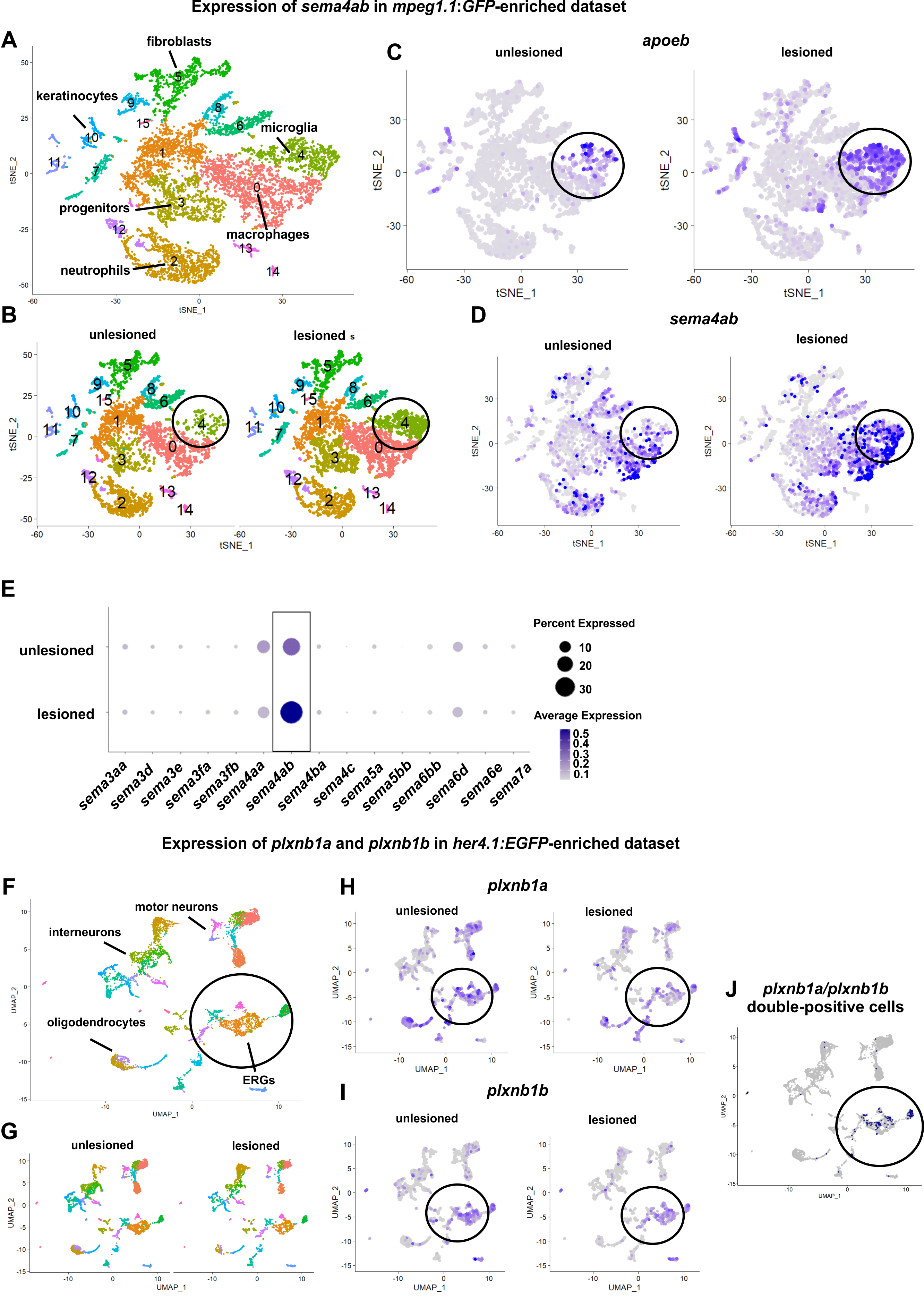

Supplement: S1 Fig — (A) UMAP showing clusters in an mpeg1.1:GFP-enriched scRNA-seq. (B) UMAPs comparing unlesioned and lesioned conditions are shown. Note an increase of the microglia cluster after injury (circle). (C,D) Feature plots comparing expression of apoeb (C) and sema4ab (D) between unlesioned and lesioned larvae, indicating increased presence of both after injury (circled). (E) Dot plot showing all semaphorins expressed in the scRNA-seq dataset before and after lesion. Note that only sema4ab is substantially expressed and also upregulated after injury (rectangle). (F) UMAP showing clusters in a her4.1:EGFP-enriched scRNA-seq. Circle indicates ERGs. (G) UMAPs indicate presence of ERGs in unlesioned and lesioned conditions. (H–J) Feature plots indicating expression of plxnb1a (H) and plxnb1b (I) in ERGs of unlesioned and lesioned larvae (circled). Plotting only cells co-expressing receptors shows strong enrichment in ERGs (J, circled). Data are described in Cavone et al., Dev Cell 2021 Jun 7;56(11):1617–1630.e6. (TIF) [file pbio.3003865.s001.tif]

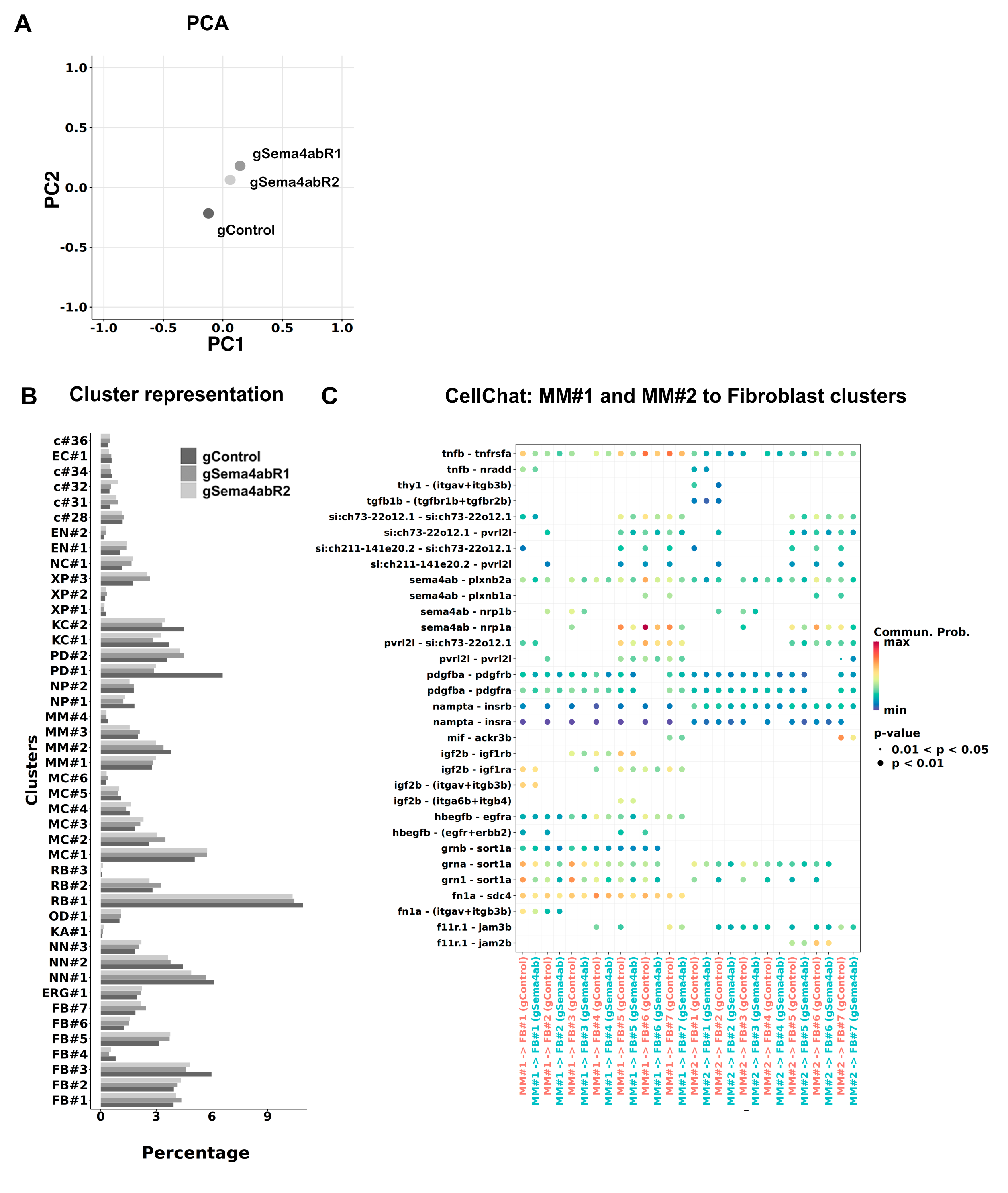

Supplement: S2 Fig — (A) Principal component analysis (PC1 and PC2) shows that gSema4ab replicates (R1 and R2) are more closely related to each other than to gControl. (B) A graph indicates similar percentages of cells per cluster across scRNA-seq samples. (C) A dot plot illustrates the communication probabilities of all ligand-receptor interactions identified by CellChat between microglia clusters MM#1 and MM#2 and fibroblast clusters. No dot indicates no interaction detected. (TIF) [file pbio.3003865.s002.tif]

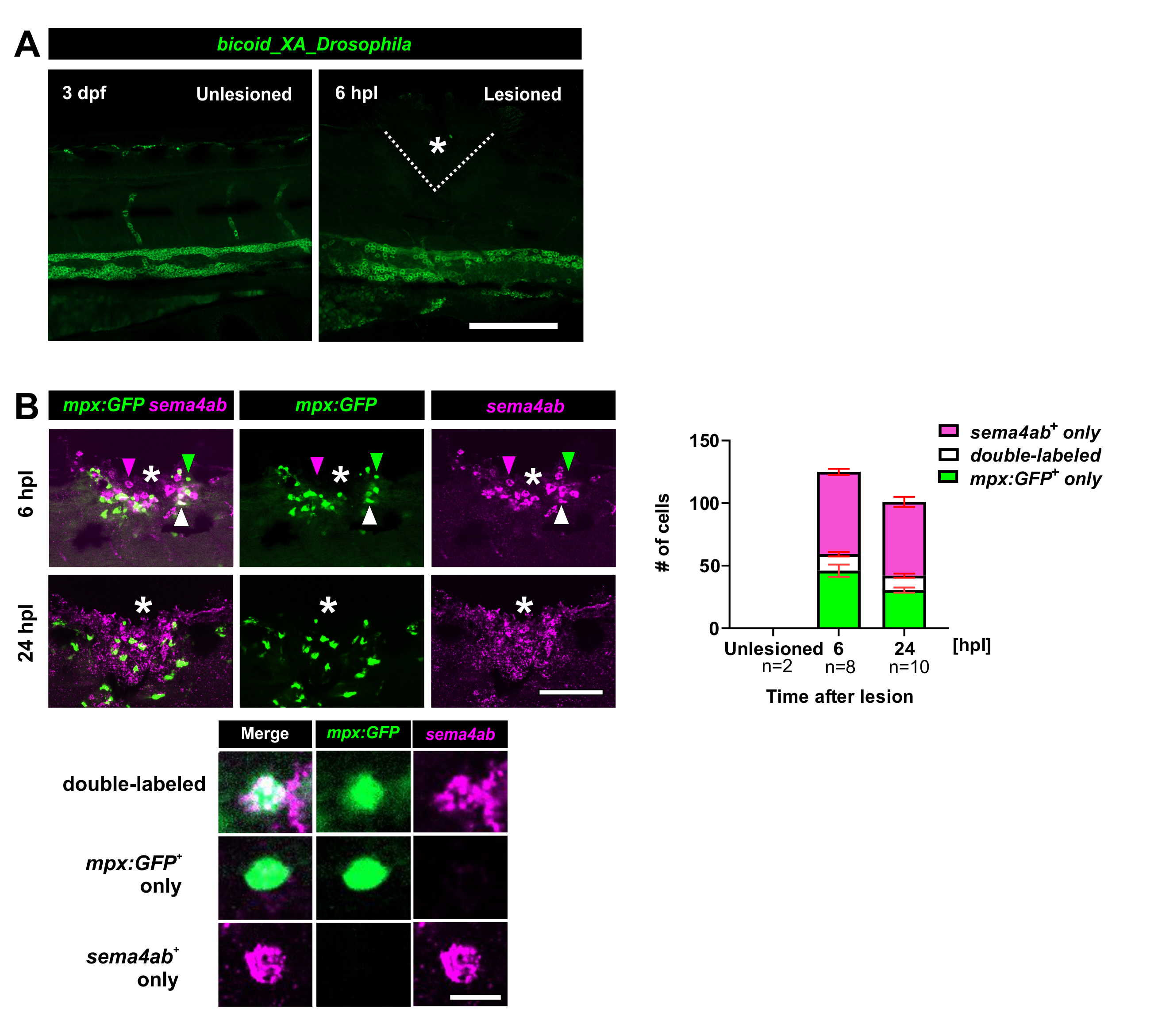

Supplement: S3 Fig — (A) HCR-FISH for the negative control gene (Drosophila BicoidXA) shows non-specific labeling in some blood vessels, but not in the spinal cord in uninjured and injured larvae. (B) Multiplexed HCR-FISH at 6 and 24 hpl shows that some sema4ab+ cells express mpx:GFP (white arrows: double-positive cells; green arrows: mpx:GFP+ only; magenta arrows: sema4ab+ only). Scale bars: 100 µm (A, B), 10 µm (inset). Data files for graphs available in S3 Data. (TIF) [file pbio.3003865.s003.tif]

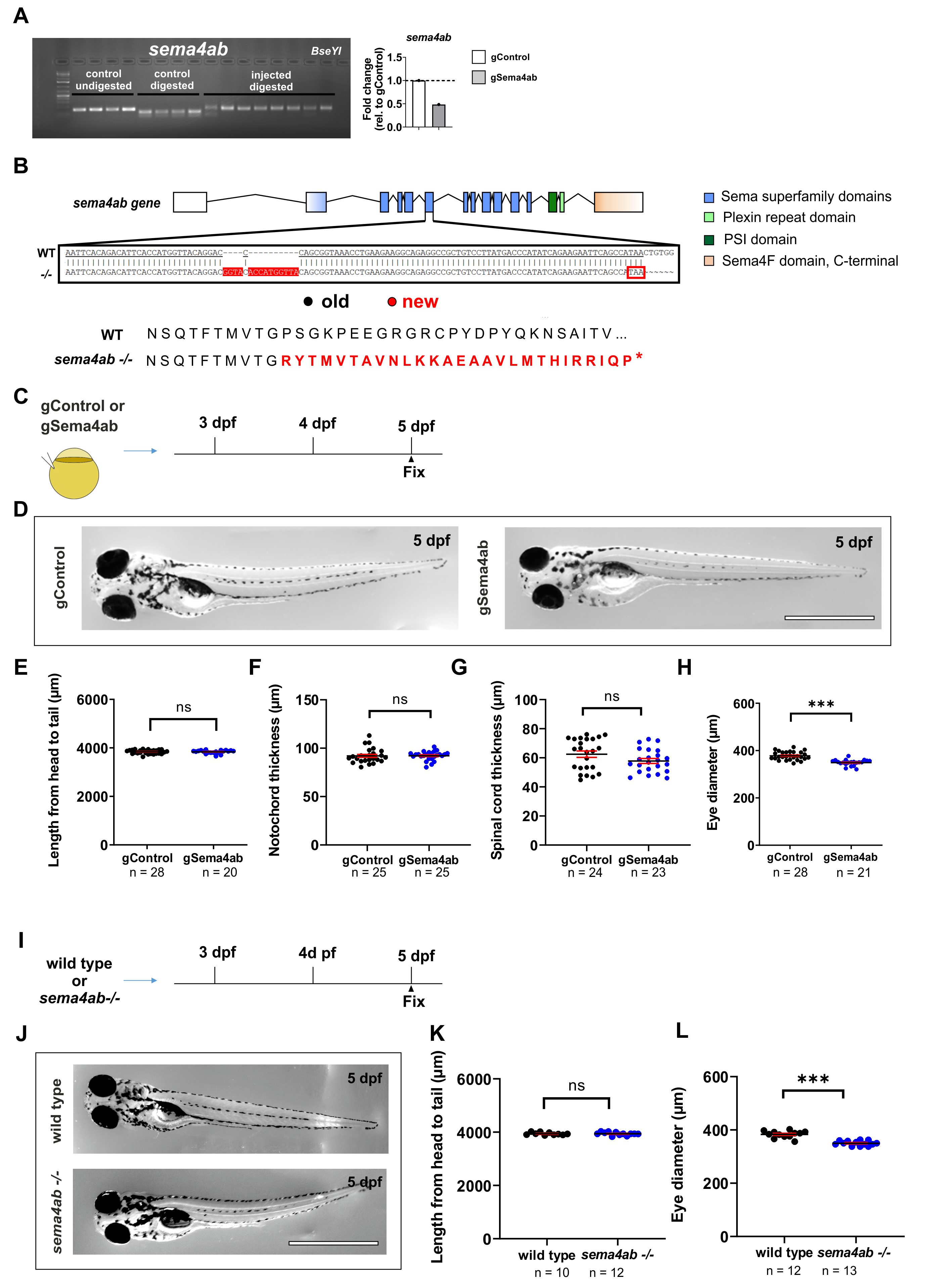

Supplement: S4 Fig — (A) RFLP and qPCR showing gRNA injection efficiency for sema4ab. For RFLP each lane represents one larva with and without digestion with the indicated restriction enzymes and with and without targeting these sites with gRNAs as indicated. Targeting the recognition sites with haCR gRNAs leads to efficient somatic mutation, as indicated by the almost complete resistance to digestion. Note a ~50% reduction in mRNA abundance detected by qRT-PCR. (B) A schematic of the sema4ab gene structure and position of the mutation in the sema4ab germline mutant is shown. Note the insertion of 14 bp in exon 6 (sequence underlaid in red) and the frameshift that predicts a stop codon (red frame and asterisk). (C) A schematic indicating the experimental design for D-H is shown. (D) Photomicrographs of gControl and gSema4ab larvae are shown at 5 dpf. (D–H) Quantifications show no differences in body length (E: gControl: 3,836 µm ± 15.65; gSema4ab: 3,838 µm ± 15.72; Unpaired t test: p = 0.9917), notochord thickness (F: gControl: 92.09 µm ± 1.527; gSema4ab: 92.20 µm ± 0.9314; Mann–Whitney U test: p = 0.3423) or spinal cord thickness (G: gControl: 62.49 µm ± 2.215; gSema4ab: 57.83 µm ± 1.677; Mann–Whitney U test: p = 0.1134) between gControl and gSema4ab larvae. Eye diameter is slightly reduced in gSema4ab larvae (H: gControl: 378.2 µm ± 3.320; gSema4ab: 349.3 µm ± 2.784; Unpaired t test: p < 0.0001). (I) A schematic indicating the experimental design for I-K is shown. (J) Photomicrographs show wild type and germline mutant larvae for sema4ab at 5 dpf. (K) No difference was observed in body length of wild type and mutant larvae (wild type: 3,941 µm ± 15.95; sema4ab −/−: 3,940 µm ± 17.50; t test: Unpaired t test: p = 0.6855). (L) Eye diameter of mutants is slightly decreased (wild type: 383.3 µm ± 3.788; sema4ab −/−: 349.9 µm ± 2.56; Unpaired t test: p < 0.0001). Each dot for qRT-PCR represents a pool of 50 larvae. β-actin was used as housekeeping gene. Raw data for qRT-PCR can be found in [file pbio.3003865.s004.tif]

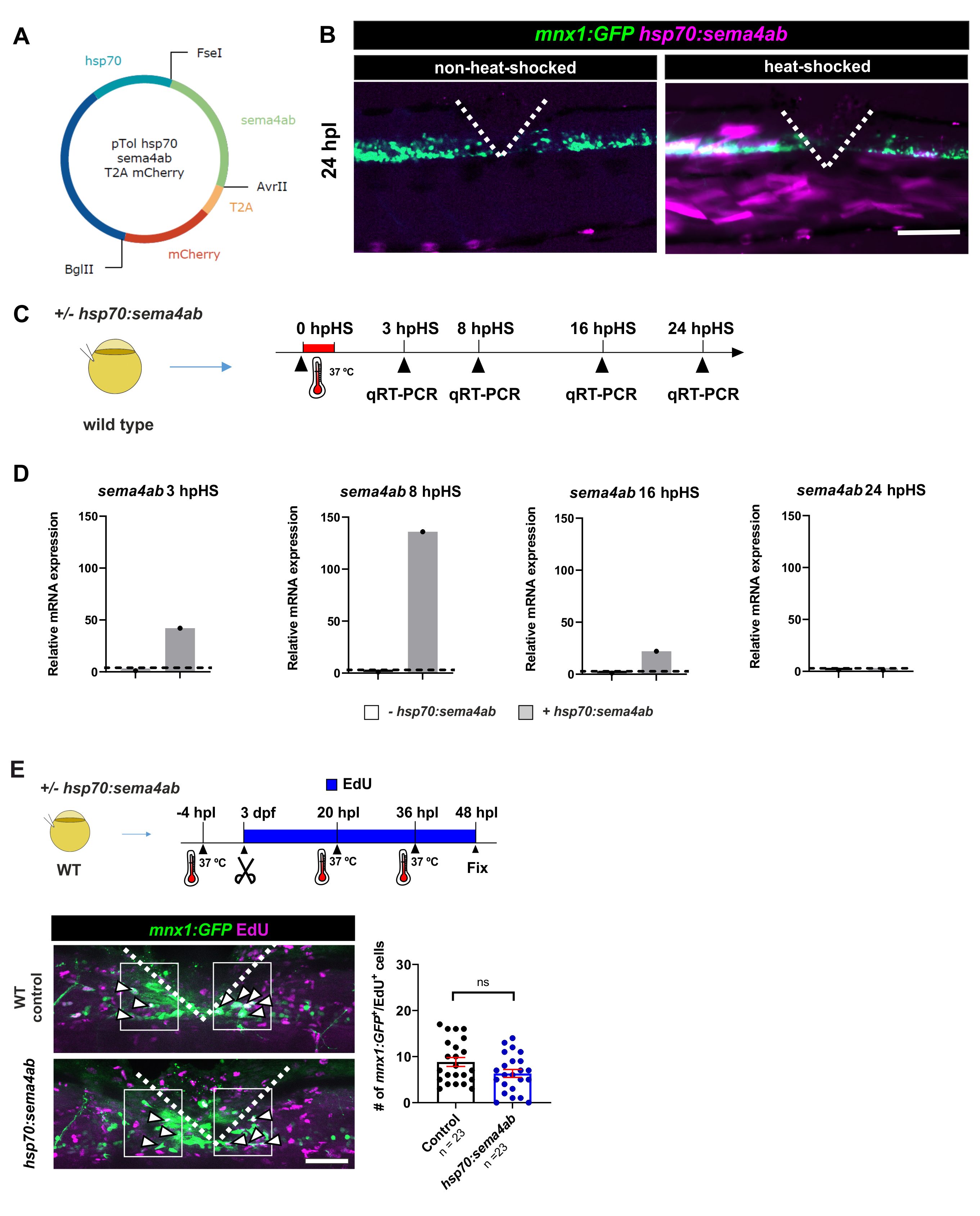

Supplement: S5 Fig — (A) A map of the heat-shock vector injected to over-express sema4ab and a reporter (mCherry) is shown. (B) Heat-shock leads to detectability of mCherry protein mainly in muscle cells surrounding the injury site (position of ventral spinal cord indicated by mnx1:GFP transgene) at 24 hpl. (C, D) Experimental timeline to assess sema4ab over-expression by qRT-PCR after a single heat shock is shown in (C). qRT-PCR analysis shows that a single heat-shock leads to strongly increased sema4ab expression for at least 16 hours post heat-shock (hpHS, D). (E) Over-expression of sema4ab does not elicit any changes in the number of newly generated neurons after spinal lesion (F; gControl: 8.8 cells per larva ± 0.98; hsp70:sema4ab: 6.4 cells per larva ± 0.86; Mann–Whitney U test: p = 0.3850). Each dot for qRT-PCR represents a pool of 50 larvae. β-actin was used as housekeeping gene. Raw data for qRT-PCR can be found in S5 and S6 Data. Error bars show SEM. Dotted lines show the injury site in B and E. Scale bars: 50 µm. Data files for graphs available in S3 Data. (TIF) [file pbio.3003865.s005.tif]

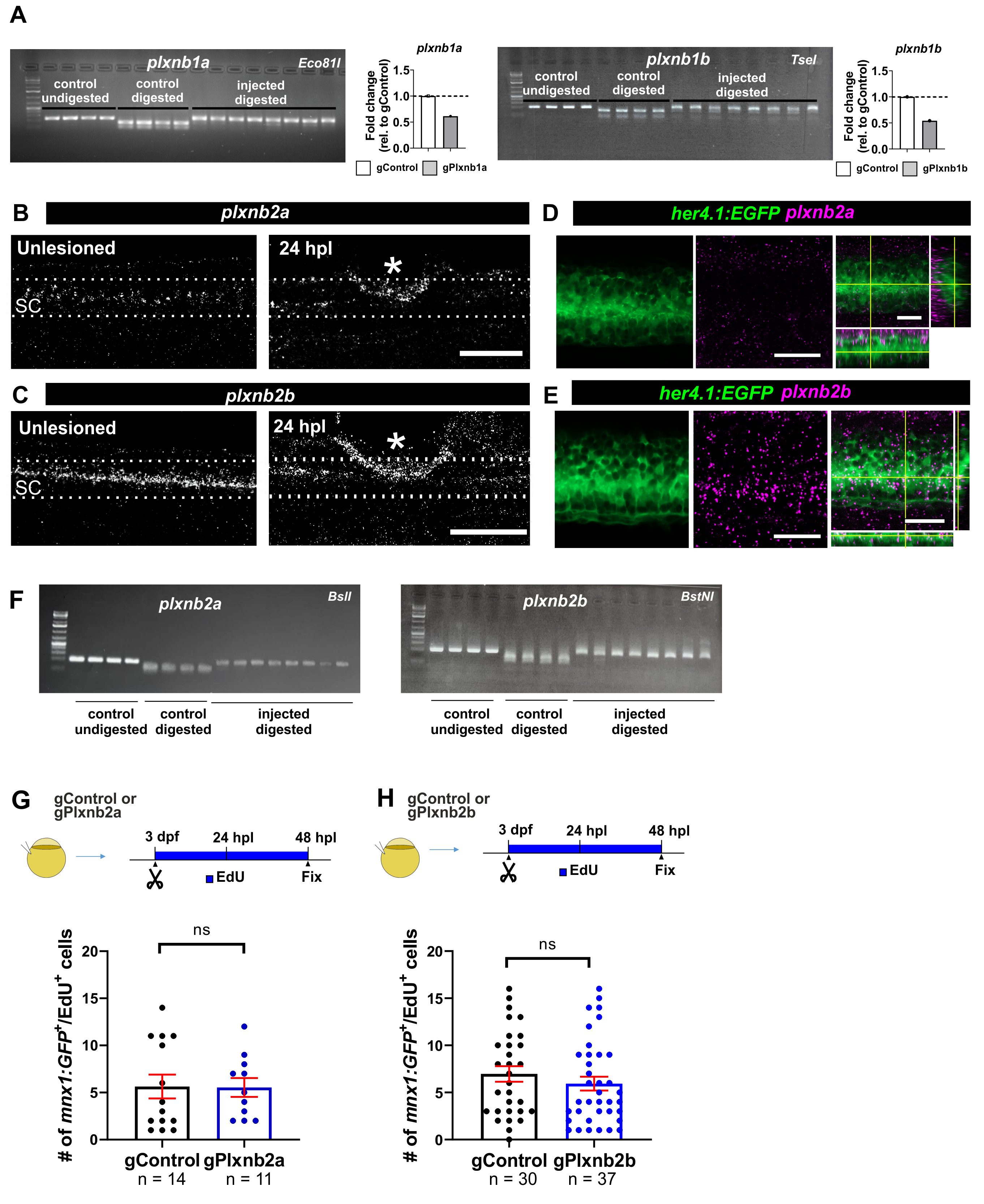

Supplement: S6 Fig — (A) RFLP and qPCR showing gRNA injection efficiency for plxnb1a and plxnb1b. For RFLP each lane represents one larva with and without digestion with the indicated restriction enzymes and with and without targeting these sites with gRNAs as indicated. Targeting the recognition sites with haCR gRNAs leads to efficient somatic mutation, as indicated by the almost complete resistance to digestion. Note a ~50% reduction in the RNA detected by qRT-PCR for either receptor. (B, C) HCR-FISH shows expression of plxnb2a (B) and plxnb2b (C) in a narrow domain in the spinal cord that does not change after lesion. Additional signal can be observed in the injury site after lesion for both genes (asterisks). (D, E) High magnifications of HCR-FISH for plxnb2a and plxnb2b show no detectable expression of plxnb2a at the level of ERGs (D), but of plxnb2b in her4.1:EGFP+ cells (E). (F) RFLP showing high gRNA injection efficiency for plxnb2a and plxnb2b. Each lane represents one larva with and without digestion with the indicated restriction enzymes and with and without targeting these sites with gRNAs as indicated. Targeting the recognition sites with haCR gRNAs leads to efficient somatic mutation, as indicated by the almost complete resistance to digestion. (G, H) haCR gene disruptions of plxnb2a (G, gControl: 5.6 cells per larva ± 1.26; gPlxnb2a: 5.5 cells per larva ± 1.00; Mann–Whitney U test: p = 0.6938) or plxnb2b (H, gControl: 6.9 cells per larva ± 0.83; gPlxnb2b: 5.9 cells per larva ± 0.73; Mann–Whitney U test: p = 0.3336) do not lead to changes in the number of newly generated motor neurons, compared to lesioned gControl-injected larvae. Each dot for qRT-PCR represents a pool of 50 larvae. β-actin was used as housekeeping gene. Raw data for qRT-PCR can be found in S5 and S6 Data. Error bars show SEM. Dotted lines delineate the position of the spinal cord. Scale bars: 100 µm (B, C), 25 µm (D, E). Data files for graphs available in S3 Data. Original gels with no adjustments can be [file pbio.3003865.s006.tif]

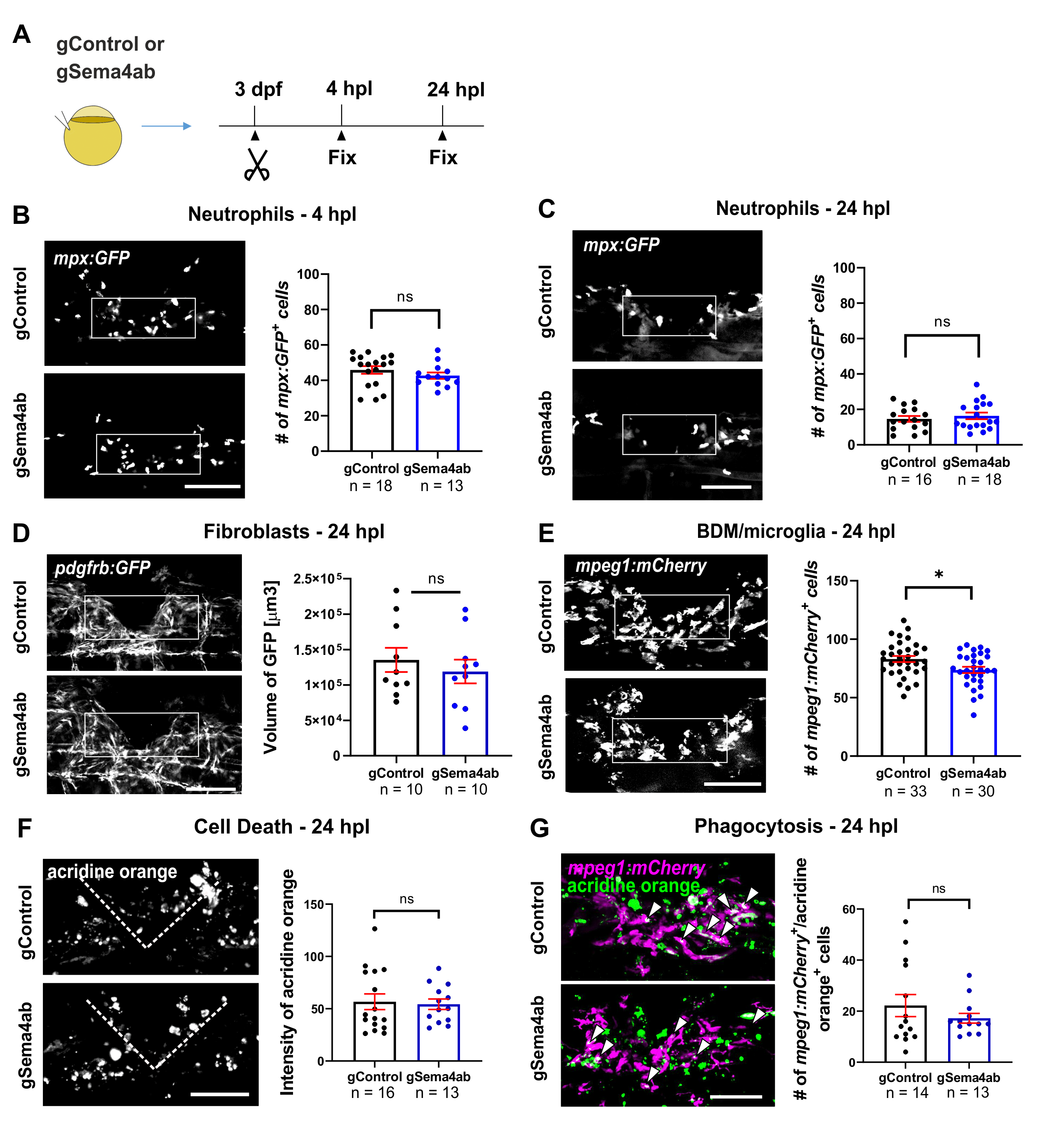

Supplement: S7 Fig — (A) A schematic indicating the experimental design for B–E is shown. (B, C) Neutrophil numbers do not show differences at 4 hpl (B, gControl: 45.89 cells per larva ± 2.1; gSema4ab: 42.69 cells per larva ± 1.8; Mann–Whitney U test: p = 0.1854) and 24 hpl (C, gControl: 14.63 cells per larva ± 1.7; gSema4ab: 16.33 cells per larva ± 2.5; Mann–Whitney U test: p = 0.5005) in sema4ab somatic mutants compared to control gRNA-injected larvae. (D) The abundance of fibroblast-like cells, measured by GFP-positive volume, shows no differences between controls and somatic mutants for sema4ab at 24 hpl (gControl: 135,342 µm3 ± 17,127; gSema4ab: 118,911 µm3 ± 16,774; Mann–Whitney U test: p = 0.6842). (E) There is a reduction in the number of BDMs/microglia in gSema4ab compared to gControl larvae larvae at 24 hpl (gControl: 83.03 cells per larva ± 9.3; gSema4ab: 73.73 cells per larva ± 3.7; Mann–Whitney U test: p = 0.0156). (F) Cell death, measured by average labeling intensity of acridine orange in the lesion site, shows no differences between experimental groups (gControl: 56.67 ± 7.5; gSema4ab 54.27 ± 5.0; Mann–Whitney U test: p = 0.8123). (G) BDMs/microglia phagocytosis, evaluated by counting mpeg1:mCherry+ cells with engulfed acridine orange+ particles, shows no differences between groups (gControl: 22.21 cells per larva ± 4.327; gSema4ab 17.23 cells per larva ± 1.912; Mann–Whitney U test: p = 0.9714). White boxes designate quantification windows. Dotted lines outline the edges of the injury site. Error bars show SEM. Scale bars: 50 µm. Data files for graphs available in S3 Data. (TIF) [file pbio.3003865.s007.tif]

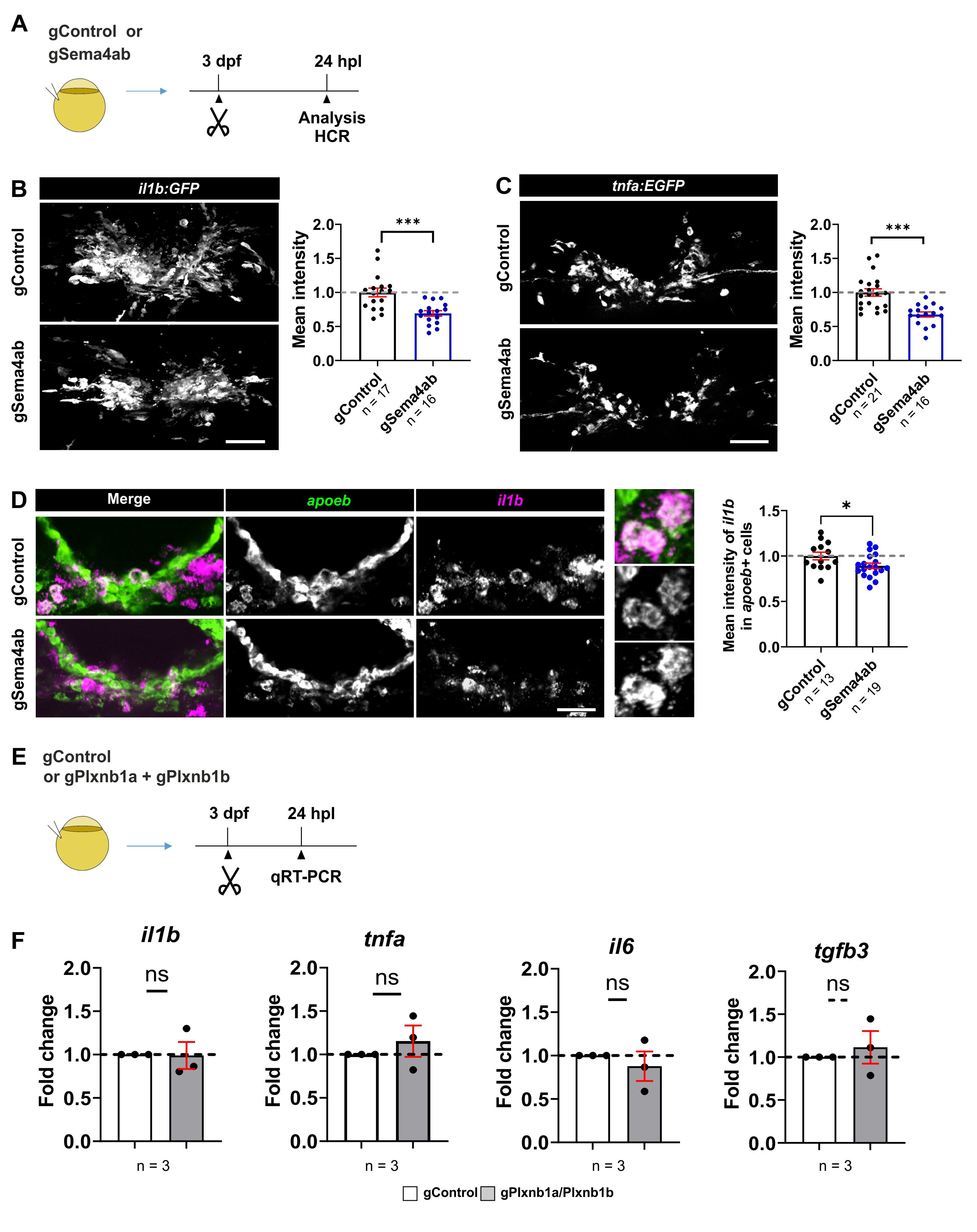

Supplement: S8 Fig — (A) A schematic indicating the experimental design for cytokine quantification in situ is shown. (B) il1b:GFP fluorescence intensity reported in transgenic fish is decreased in lesioned sema4ab somatic mutants compared to control gRNA-injected fish at 24 hpl (−1.45 fold-change; Unpaired t test: p = 0.0004). (C) tnfa:EGFP fluorescence intensity reported in transgenic animals is decreased in lesioned sema4ab somatic mutants compared to control injected fish at 24 hpl (−1.49 fold-change; Unpaired t test: p < 0.0001). (D) il1b expression in microglia (apoeb+ cells) detected by HCR shows a reduction of 11% in mean intensity (Unpaired t test: p = 0.0380). (E) A schematic indicating the experimental design for qRT-PCR. (F) qRT-PCR analyses of major pro-inflammatory cytokines (il1b, tnfa, il6) and tgfb3 in plxnb1a/plxnb1b double somatic mutants show no differences to injured control gRNA-injected larvae at 24 hpl (One-sample t test; il1b: p = 0.537; tnfa: p = 0.4840; il6: p = 0.5464; tgfb3: p = 0.687). Each dot for qRT-PCR represents a pool of 50 larvae. β-actin was used as housekeeping gene. Raw data for qRT-PCR can be found in S5 and S6 Data. Error bars show SEM. Scale bars: 50 µm. Data files for graphs available in S3 Data. (TIF) [file pbio.3003865.s008.tif]

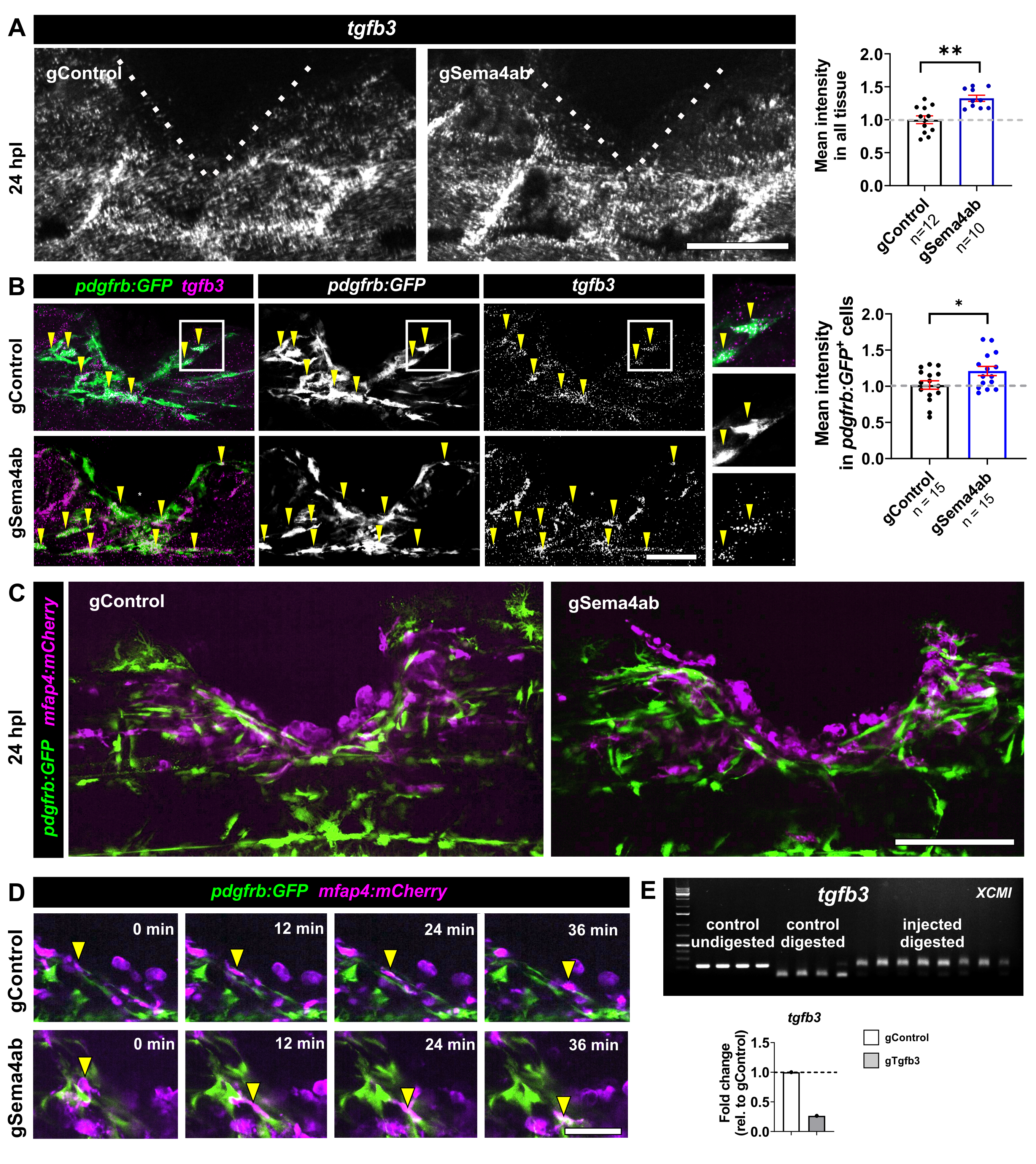

Supplement: S9 Fig — (A) HCR-FISH for tgfb3 shows an increase in fluorescence intensity in lesioned sema4ab somatic mutants compared to lesioned control gRNA-injected fish at 24 hpl (gControl: 1.0 ± 0.058; gSema4ab: 1.3 ± 0.046; Unpaired t test: p = 0.0011). Dotted lines show the injury site. (B) HCR-FISH at 24 hpl shows tgfb3 expression in fibroblast-like cells (pdgfrb:GFP+; arrowheads). The mean intensity of the tgfb3 signal in pdgfrb:GFP+ cells shows an increase in sema4ab haCR-injected larvae at 24 hpl (+1.22-fold change, Unpaired t test p = 0.0322). (C) Individual frames of time-lapse movies show mfap4:mCherry+ cells in close contact to pdgfrb:GFP+ cells in both control gRNA-injected animals and in sema4ab somatic mutants. (D) Time series of movie frames show that mfap4:mCherry+ cells (arrowheads) migrate along fibroblast processes in both conditions. (E) RFLP and qPCR showing gRNA injection efficiency for tgfb3. For RFLP each lane represents one larva with and without digestion with the indicated restriction enzymes and with and without targeting these sites with gRNAs as indicated. Targeting the recognition sites with haCR gRNAs leads to efficient somatic mutation, as indicated by the almost complete resistance to digestion. Note a ~ 50% decay in the RNA detected by qPCR. Each dot for qRT-PCR represents a pool of 50 larvae. β-actin was used as housekeeping gene. Raw data for qRT-PCR can be found in S5 and S6 Data. Scale bars: 100 µm (A, C,), 50 µm (B) 15 µm (D). Data files for graphs available in S3 Data. Original gels with no adjustments can be found in S1 Raw Images. (TIF) [file pbio.3003865.s009.tif]

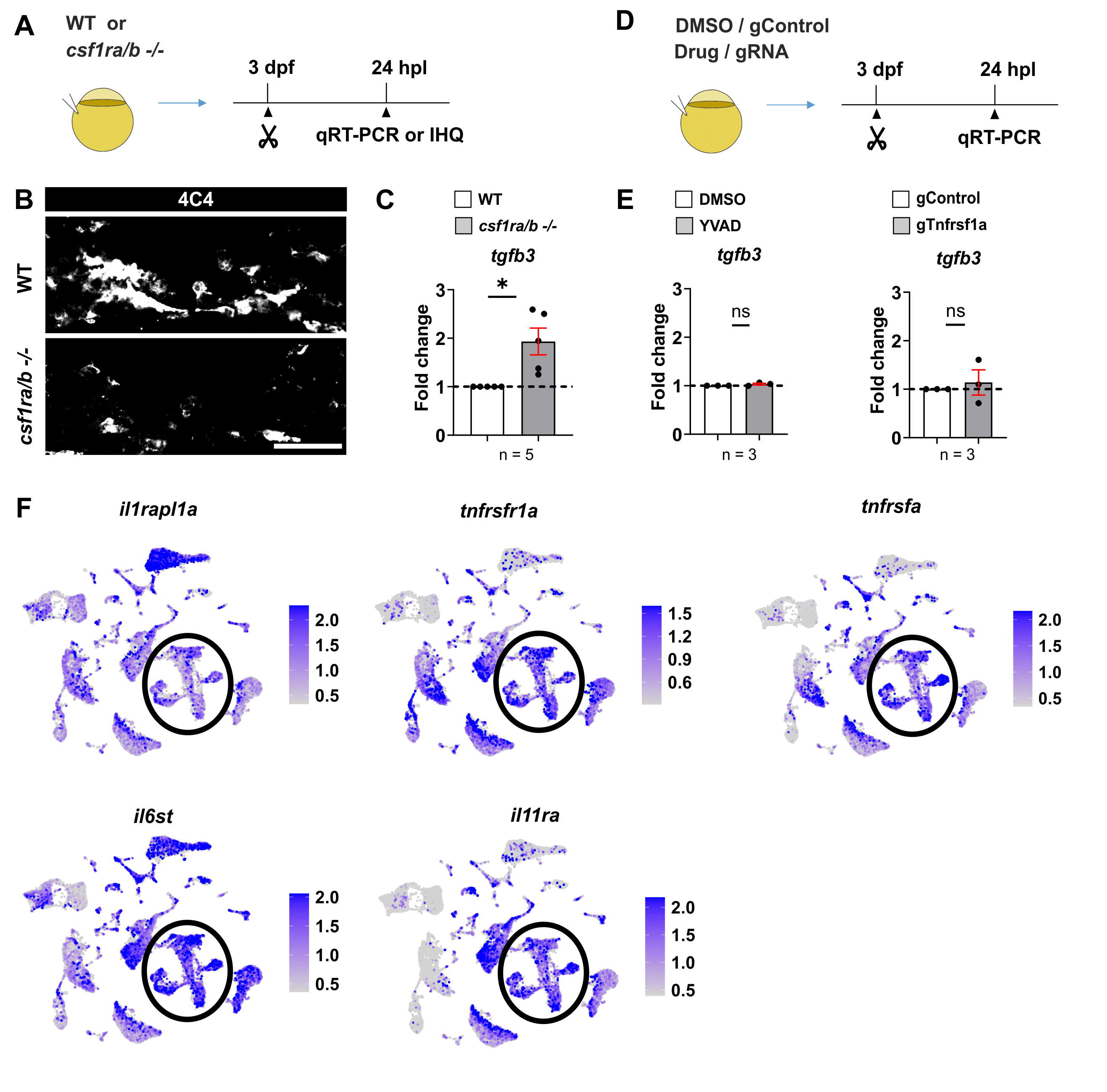

Supplement: S10 Fig — (A–C) A Microglia depletion line shows an increase in tgfb3 levels at 24 hpl by qRT-PCR (One-sample t test: p = 0.0281). (D, E) Impairing cytokine signaling by YVAD (impairs il1b cleavage) and tnfrsf1a loss of function, results in no meaningful changes in tgfb3 levels at 24 hpl by qRT-PCR (One-sample t test; YVAD: p = 0.2303; gTnfrsf1a: p = 0.6461). (F) Feature plots showing main pro-inflammatory cytokine receptor expression pattern. Note a strong expression in fibroblasts (circle). Each dot for qRT-PCR represents a pool of 50 larvae. β-actin was used as housekeeping gene. Raw data for qRT-PCR can be found in S5 and S6 Data. Scale bars: 50 µm. Data files for graphs available in S3 Data. (TIF) [file pbio.3003865.s010.tif]

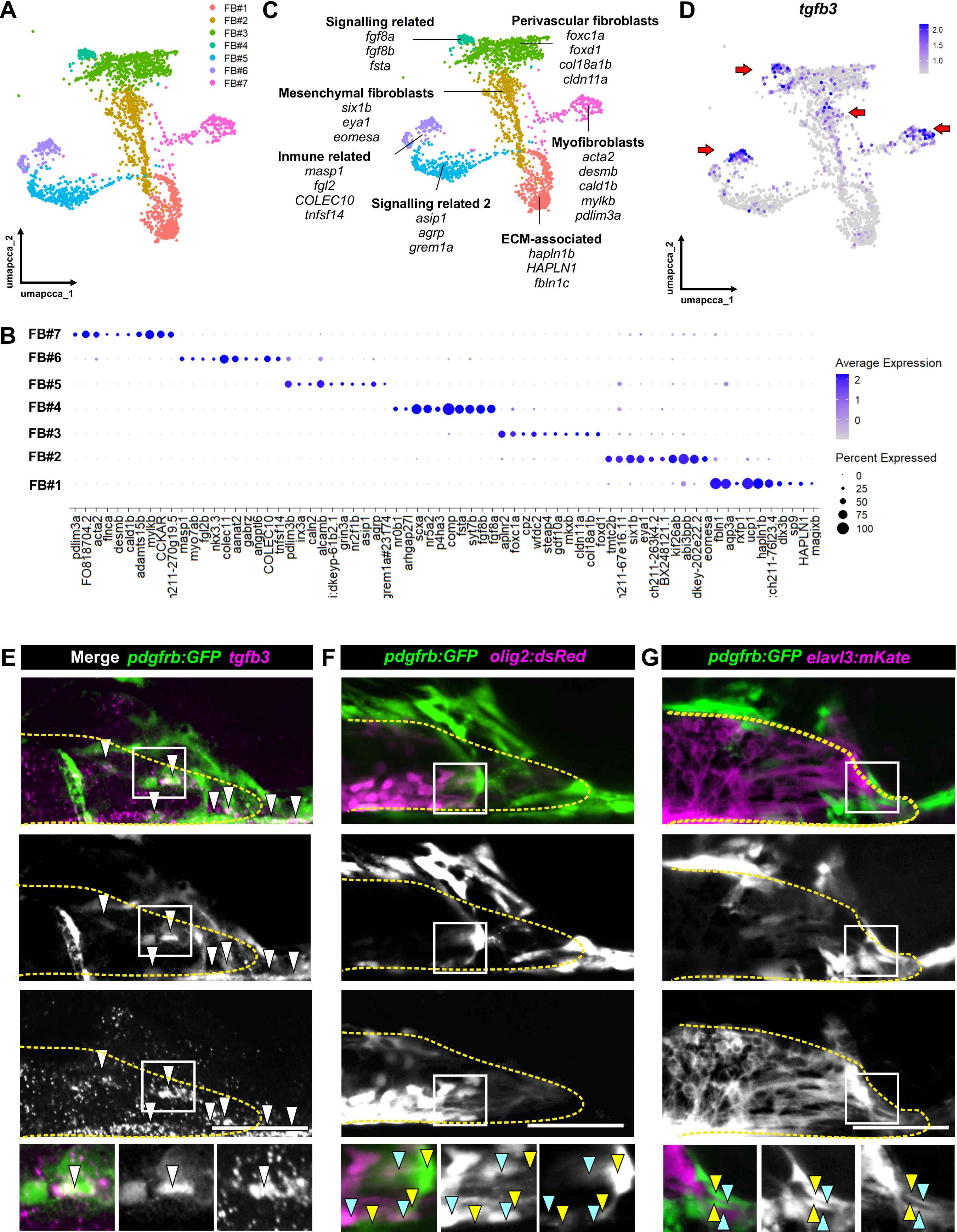

Supplement: S11 Fig — (A) UMAP of fibroblasts subset showing 7 clusters of cells. (B) Top 10-ranked gene analysis showing differential expressing genes among clusters. (C) UMAP showing genes used for annotation and tentative cluster names. (D) Feature plot showing expression pattern of tgfb3. Arrows point enriched clusters for the gene. (E) Single-Stacks of the lesion site at the level of the spinal cord showing tgfb3-expressing fibroblasts (arrow). (F) Live imaging showing that fibroblasts (green, yellow arrows) are in the vicinity of motor neuron progenitors (magenta, cyan arrows). (G) Live imaging showing that fibroblasts (green, yellow arrows) are in close contact with growing axons (magenta, cyan arrows). Yellow dotted lines mark the spinal cord. Scale bars: 50 µm. (TIF) [file pbio.3003865.s011.tif]

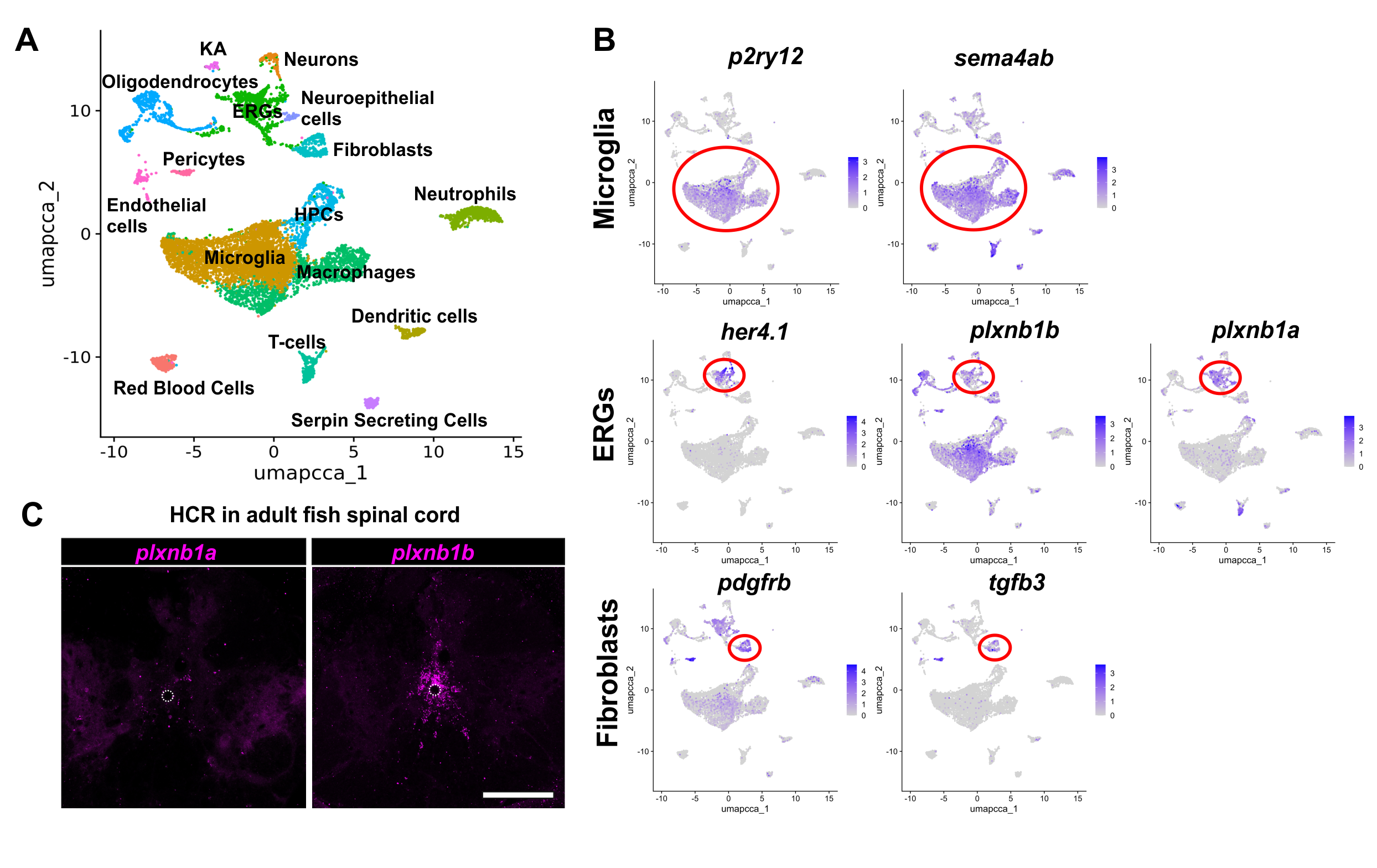

Supplement: S12 Fig — (A) UMAP of the main cell types in the lesion site of adult zebrafish from Cigliola et al. 2023 Nat Commun 14:4857 is shown. (B) Feature plots show the expression of reference genes for relevant cell types (microglia: p2ry12; ERGs: her4.1; and fibroblasts-like cells: pdgfrb), as well as the expression of genes of interest for this study, sema4ab, plxnb1a, plxnb1b and tgfb3. Circles label expression in cell types that is conserved between larvae and adults. (C) HCR-FISH in cross sections of unlesioned spinal cords of 4-month-old adult zebrafish show expression of plxnb1a and plxnb1b in the ventricular zone. Dorsal is up and ventral is down. Dotted circle shows the central canal. Scale bar: 50 µm. (TIF) [file pbio.3003865.s012.tif]

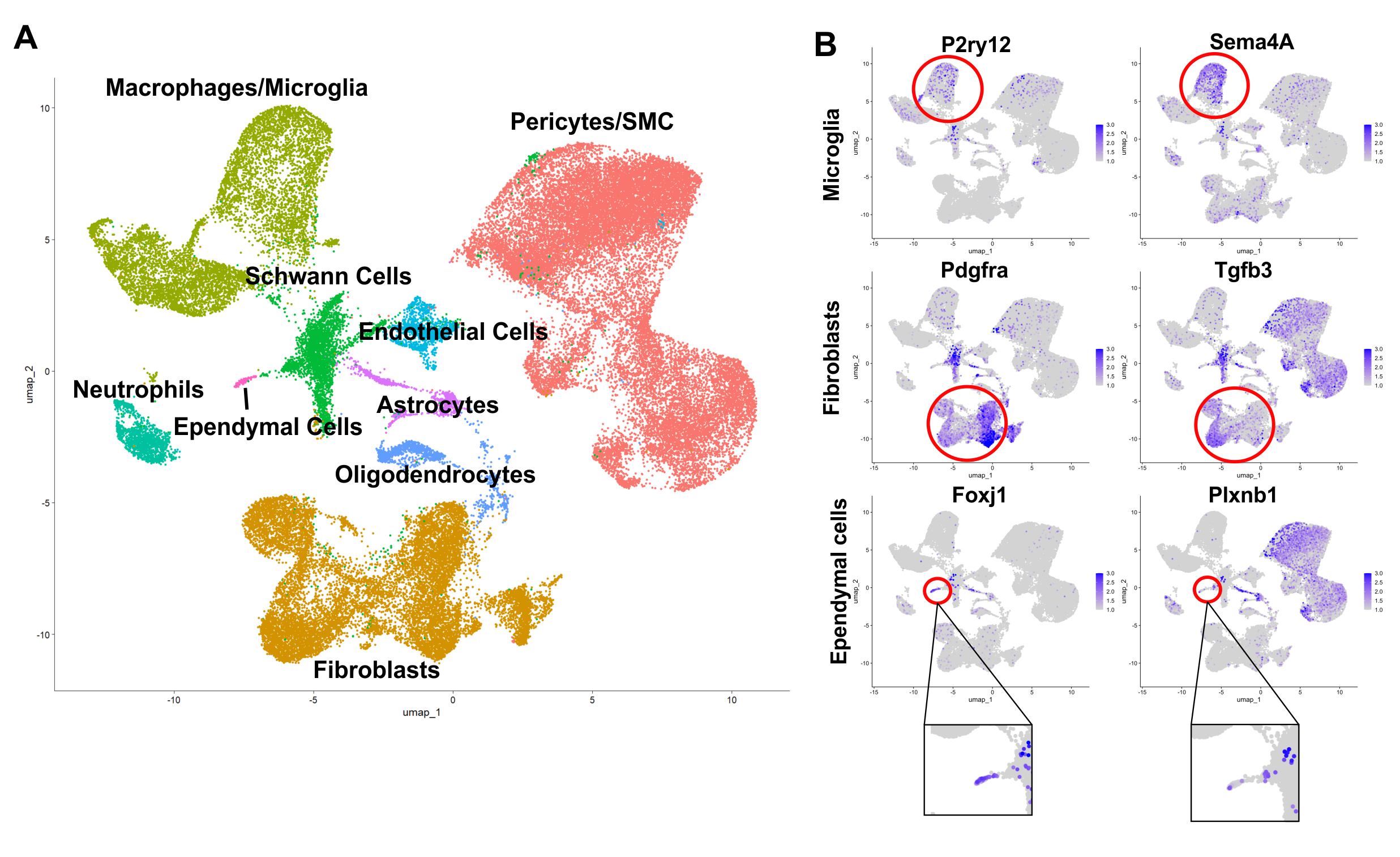

Supplement: S13 Fig — (A) A UMAP representation indicating the main cell types in a mouse spinal lesion from Xue et al., 2024 Nat Commun 15:6321 is shown. (B) Expression pattern of mouse ortholog genes Sema4A, Tgfb3 and Plxnb1 is conserved in microglia (P2ry12), fibroblasts (Pdgfra) and ependymal cells (Foxj1), respectively. Circles label expression in cell types that is conserved between zebrafish and mouse. (TIF) [file pbio.3003865.s013.tif]

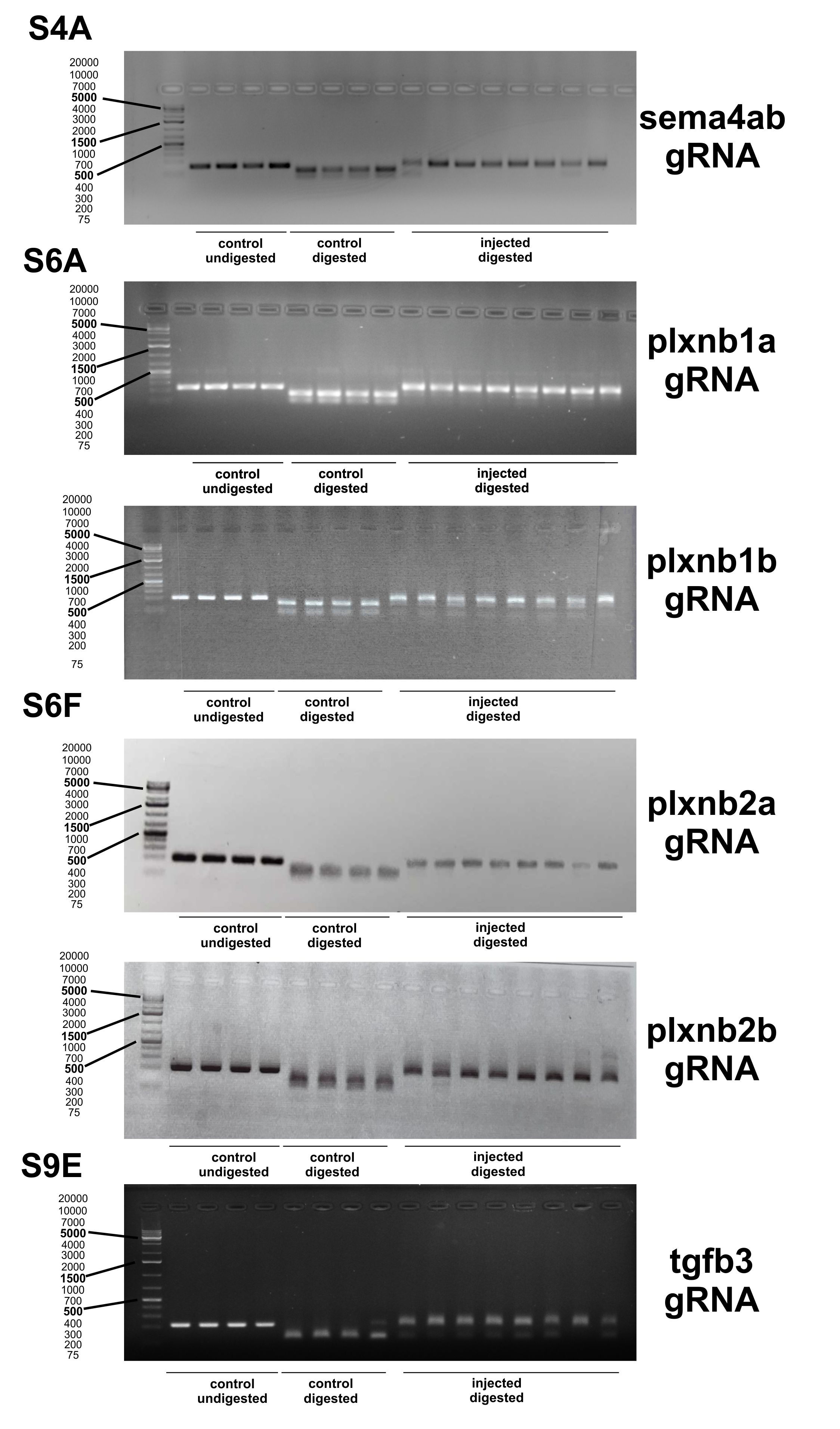

Supplement: S1 Raw Images — Gels were acquired using the Axygen Gel Documentation System (Corning, NY, USA) and imaged from the pocket to the electrophoresis front. No pockets or lane were excluded from the image. (TIF) [file pbio.3003865.s032.tif]
