# Supplementary material for: The microglia-derived protein Sema4ab attenuates regenerative neurogenesis after spinal cord injury in zebrafish
Source: PLoS Biol. 2026 Jun 18;24(6):e3003865. doi: 10.1371/journal.pbio.3003865 (PMC13309017; doi:10.1371/journal.pbio.3003865)
Supplement: S1 Table — (DOCX) [file pbio.3003865.s014.docx]

|  | **gControl** | **gSema4abR1** | **gSema4abR2** |
| --- | --- | --- | --- |
| **All Cells** | 14389 | 14384 | 14163 |
| **Cells removed** | 2601 | 2763 | 2833 |
| **Cells remained** | 11788 | 11621 | 11330 |
| **Percentage** | 81,92 | 80,79 | 80,00 |
