# Supplementary material for: The microglia-derived protein Sema4ab attenuates regenerative neurogenesis after spinal cord injury in zebrafish
Source: PLoS Biol. 2026 Jun 18;24(6):e3003865. doi: 10.1371/journal.pbio.3003865 (PMC13309017; doi:10.1371/journal.pbio.3003865)
Supplement: S2 Table — (DOCX) [file pbio.3003865.s015.docx]

|  | **nFeature_RNA** | | | **nCount_RNA** | | |
| --- | --- | --- | --- | --- | --- | --- |
|  | **mean** | **median** | **total** | **mean** | **median** | **total** |
| **gControl** | 2419,3 | 2343 | 28518887 | 9925,6 | 7101 | 117002401 |
| **gSema4abR1** | 2218,7 | 2038 | 25783341 | 9548,0 | 6581 | 110957581 |
| **gSema4abR2** | 2217,1 | 2045 | 25119458 | 9768,7 | 6839 | 110678876 |
