# Supplementary material for: The microglia-derived protein Sema4ab attenuates regenerative neurogenesis after spinal cord injury in zebrafish
Source: PLoS Biol. 2026 Jun 18;24(6):e3003865. doi: 10.1371/journal.pbio.3003865 (PMC13309017; doi:10.1371/journal.pbio.3003865)
Supplement: S3 Table — (DOCX) [file pbio.3003865.s016.docx]

|  | ***sema4ab* mapped total reads** | **target site total reads** | **target site intact reads** |
| --- | --- | --- | --- |
| **gControl** | 16945 | 151 | 151 |
| **gSema4abR1** | 9880 | 4 | 0 |
| **gSema4abR2** | 10384 | 6 | 0 |
