# Supplementary material for: The microglia-derived protein Sema4ab attenuates regenerative neurogenesis after spinal cord injury in zebrafish
Source: PLoS Biol. 2026 Jun 18;24(6):e3003865. doi: 10.1371/journal.pbio.3003865 (PMC13309017; doi:10.1371/journal.pbio.3003865)
Supplement: S4 Table — (DOCX) [file pbio.3003865.s017.docx]

| **scRNA-seq reads of *sema4ab* gRNA target site in gSema4ab** | |
| --- | --- |
| **Reference sequence** | CACAGACATTCACCATGGTTACAGGACCCAGCGGTAAACCTGAAGAAG |
| **gSema4abR1** | . .. CAGACATTCACCATGGTTACA---------------------------------------------------- |
|  | .. . CAGACATTCACCATGGTTACAGG-------------------TAAACCTGAAGAAG |
|  | . ... . . GACATTCACCATGGTTA--------------------------------------------------------- |
|  | . .. .. . GACATTCACCATGGTTACAG---------------CGGTAAACCTGAAGAAG |
| **gSema4abR2** | CACAGACATTCACCATGGTTACAGG------------------------------------------------ |
|  | CACAGACATTCACCATGGTTACAGG--------------------AAACCTGAAGAAG |
|  | CACAGACATTCACCATGGTTA--------------------------------------------------------- |
|  | . .. CAGACATTCACCATGGTTACAG---------------CGGTAAACCTGAAGAAG |
|  | . .. . . . .ACATTCACCATGGTTACAGGAACACATTCACCATGGTTACAGGA |
|  | CACAGACATTCACCATGGTTA--------------------------------AACCTGAAGAAG |
| Non-targeted sequence Targeted sequence Missing/New | |
