# Supplementary material for: The microglia-derived protein Sema4ab attenuates regenerative neurogenesis after spinal cord injury in zebrafish
Source: PLoS Biol. 2026 Jun 18;24(6):e3003865. doi: 10.1371/journal.pbio.3003865 (PMC13309017; doi:10.1371/journal.pbio.3003865)
Supplement: S5 Table — The table shows the correspondence between each cluster obtained by unsupervised clustering and the correspondent main cell type and cluster name assigned. (DOCX) [file pbio.3003865.s018.docx]

| Annotation of Seurat clusters | | | |
| --- | --- | --- | --- |
| Cluster | **Main cell type** | **Marker genes** | **Abbreviation** |
| 28 | c#28 | *NA* | c#28 |
| 31 | c#31 | *NA* | c#31 |
| 32 | c#32 | *NA* | c#32 |
| 34 | c#34 | *NA* | c#34 |
| 36 | c#36 | *NA* | c#36 |
| 35 | Endothelial Cells | *kdrl, cldn5b* | EC#1 |
| 40 | ENS (enteric nervous system) | *phox2aa, phox2b* | EN#2 |
| 27 | ENS (enteric nervous system) | *phox2aa, phox2b* | EN#1 |
| 10 | Fibroblast-Like Cells | *col12a1a, pdgfrb* | FB#5 |
| 17 | Fibroblast-Like Cells | *col12a1a, pdgfrb* | FB#7 |
| 25 | Fibroblast-Like Cells | *col12a1a, pdgfrb* | FB#6 |
| 3 | Fibroblast-Like Cells | *col12a1a, pdgfrb* | FB#3 |
| 5 | Fibroblast-Like Cells | *col12a1a, pdgfrb* | FB#2 |
| 33 | Fibroblast-Like Cells | *col12a1a, pdgfrb* | FB#4 |
| 6 | Fibroblast-Like Cells | *col12a1a, pdgfrb* | FB#1 |
| 9 | Keratinocytes | *pfn1, krt4* | KC#2 |
| 12 | Keratinocytes | *pfn1, krt4* | KC#1 |
| 11 | Macrophages/Microglia | *mfap4, mpeg1.1* | MM#2 |
| 37 | Macrophages/Microglia | *mfap4, mpeg1.1* | MM#4 |
| 15 | Macrophages/Microglia | *mfap4, mpeg1.1* | MM#1 |
| 21 | Macrophages/Microglia | *mfap4, mpeg1.1* | MM#3 |
| 30 | Muscle | *meox1, mylz3* | MC#5 |
| 13 | Muscle | *meox1, mylz3* | MC#2 |
| 2 | Muscle | *meox1, mylz3* | MC#1 |
| 24 | Muscle | *meox1, mylz3* | MC#4 |
| 19 | Muscle | *meox1, mylz3* | MC#3 |
| 38 | Muscle | *meox1, mylz3* | MC#6 |
| 8 | Neural cells | *elavl3, elavl4* | NN#2 |
| 1 | Neural cells | *elavl3, elavl4* | NN#1 |
| 20 | Neural cells | *elavl3, elavl4* | NN#3 |
| 42 | Neural cells (Kolmer Agduhr) | *npcc, sst1.1* | KA#1 |
| 18 | Neural cells | *fabp7a, sox2* | ERG#1 |
| 29 | Neural cells (oligodendrocyte) | *mbpb, mpz* | OD#1 |
| 26 | Neutrophils | *mpx, lyz* | NP#1 |
| 22 | Neutrophils | *mpx, lyz* | NP#2 |
| 23 | Notochord | *shha, gas2b* | NC#1 |
| 4 | Peridermis | *krt5, krt91* | PD#1 |
| 7 | Peridermis | *krt5, krt91* | PD#2 |
| 0 | Red Blood Cells | *hbbe1.3, hbbe2* | RB#1 |
| 4 | Red Blood Cells | *hbbe1.3, hbbe2* | RB#2 |
| 43 | Red Blood Cells | *hbbe1.3, hbbe2* | RB#3 |
| 41 | Xantophores | *gch2, aox5* | XP#1 |
| 16 | Xantophores | *gch2, aox5* | XP#3 |
| 39 | Xantophores | *gch2, aox5* | XP#2 |
