# Supplementary material for: The microglia-derived protein Sema4ab attenuates regenerative neurogenesis after spinal cord injury in zebrafish
Source: PLoS Biol. 2026 Jun 18;24(6):e3003865. doi: 10.1371/journal.pbio.3003865 (PMC13309017; doi:10.1371/journal.pbio.3003865)
Supplement: S6 Table — (DOCX) [file pbio.3003865.s019.docx]

| Top 10 downregulated genes in MM#1 (gSema4ab vs gControl) | | | | | | |
| --- | --- | --- | --- | --- | --- | --- |
| Gene | **p_val** | **avg_log2FC** | **pct.1** | **pct.2** | **p_val_adj** | **Fold Change** |
| fthl31 | 4.53E-10 | -2.8108973 | 0.08 | 0.259 | 1.19E-05 | -7.0172090 |
| grn1 | 7.24E-18 | -2.7803628 | 0.367 | 0.625 | 1.90E-13 | -6.8702511 |
| lyz | 2.71E-09 | -2.5651207 | 0.219 | 0.416 | 7.09E-05 | -5.9180452 |
| dyrk1aa | 1.81E-08 | -2.2728607 | 0.033 | 0.162 | 0.00047467 | -4.8328048 |
| acacb | 1.75E-06 | -2.1746032 | 0.062 | 0.181 | 0.04588534 | -4.5146157 |
| fam78ab | 2.73E-06 | -2.1453837 | 0.068 | 0.191 | 0.07138602 | -4.4240991 |
| mxf | 4.21E-09 | -2.1047832 | 0.24 | 0.438 | 0.00011024 | -4.3013312 |
| b4galt1 | 1.27E-06 | -2.0767341 | 0.05 | 0.166 | 0.03338913 | -4.2185117 |
| ifitm1 | 2.02E-06 | -2.0587836 | 0.13 | 0.266 | 0.05283023 | -4.1663487 |
| scp2b | 1.19E-06 | -2.0462101 | 0.092 | 0.225 | 0.03110672 | -4.1301956 |
