# Supplementary material for: The microglia-derived protein Sema4ab attenuates regenerative neurogenesis after spinal cord injury in zebrafish
Source: PLoS Biol. 2026 Jun 18;24(6):e3003865. doi: 10.1371/journal.pbio.3003865 (PMC13309017; doi:10.1371/journal.pbio.3003865)
Supplement: S7 Table — Each column represents the targeted sequence of a gene and the primers and restriction enzymes used to validate the efficiency of the mutation. (DOCX) [file pbio.3003865.s020.docx]

| **List of targeted genes using CRISPR/Cas9** | | | |
| --- | --- | --- | --- |
| **Gene** | **RE** | **Sequence** | **Primer** |
| ***sema4ab*** | BseYI | ACCATGGTTACAGGACCCAG | F: GCTCATGTCCACAGAATTCACA |
|  |  |  | R: GCCACTGTTGATATACTTACCCA |
| ***plxnb1a*** | Eco81I | GGCAGAACTGCCCTCAGGCT | F: GAAGTGGATGAGGAAACCGG |
|  |  |  | R: CGTATCTACAGGCCTCTCGG |
| ***plxnb1b*** | TseI | CACCATACTGTGTTGCTGCG | F: TGGGATGGTGTGTGTGACTT |
|  |  |  | R: CGTCTCTTCCTCTGCCTCAA |
| ***plxnb2a*** | BsaIl | ACCTCCCGGCTTGTCCTCAAG | F: CATGGTTCCGTTTCGCCTAA |
|  |  |  | R: CATCGACACATGAGCTGGCA |
| ***plxnb2b*** | BstNI | GGACCACGCTGGCCGGCTGC | F: AAAGTGGCTCGGATGGAGAC |
|  |  |  | R: GAGTGCAGTACCTTGACGGA |
| ***tgfb3*** | XCMI | GAATCCATCCAGCAGATCCC | F: GGGTCAGATCCTCAGCAAAC |
|  |  |  | R: GAGATCCCTGGATCATGTTGA |
| ***tnfrsfr1a*** | BstNI | ATAGTTTGCTTGTTCCAGGT | F: CAGGAATGCAGTGCAGAAAA |
|  |  |  | R: TGACAATCCAGTGCTATTTTGG |
