# Supplementary material for: The microglia-derived protein Sema4ab attenuates regenerative neurogenesis after spinal cord injury in zebrafish
Source: PLoS Biol. 2026 Jun 18;24(6):e3003865. doi: 10.1371/journal.pbio.3003865 (PMC13309017; doi:10.1371/journal.pbio.3003865)
Supplement: S8 Table — (DOCX) [file pbio.3003865.s021.docx]

| Primers use for cloning the *mfap4:mCherry-CAAX;myl7:mCerulean* vector | |
| --- | --- |
| Primer | **Primer sequence (5’-3’)** |
| *mfap4* | **Fw:** ttt ttt gag ctc ctc gag gcg ttt ctt ggt ac |
|  | **Rev:** ttt ttt ggc gcg cct gga tcc cac gat cta aag tca tga ag |
| *mCherry-CAAX* | **Fw:** aaa aaa gaa ttc gcc acc atg gtg agc aag ggc gag g |
|  | **Rev:** aaa aaa tta att aat cag gag agc aca cac ttg cag ctc atg cag ccg ggg cca ctc tca tca gga ggg ttc agc tta gat ctg agt ccg gac ttg tac agc tcg tcc atg ccg aga gtg |
