# Supplementary material for: The microglia-derived protein Sema4ab attenuates regenerative neurogenesis after spinal cord injury in zebrafish
Source: PLoS Biol. 2026 Jun 18;24(6):e3003865. doi: 10.1371/journal.pbio.3003865 (PMC13309017; doi:10.1371/journal.pbio.3003865)
Supplement: S9 Table — (DOCX) [file pbio.3003865.s022.docx]

| Primers use for cloning the *hsp70l:sema4ab-t2a-mcherry* vector | |
| --- | --- |
| Primer | **Primer sequence (5’-3’)** |
| *sema4ab* | **Fw:** tatGGCCGGCCCAGGATGGTGATTTCGGCATCACT |
|  | **Rev:** atatCCTAGGGATGGGTGTGTTGGGCAGGT |
| *T2a-mCherry* | **Fw:** atatCCTAGGGAGGGCAGAGGAAGTCTTCTAACATGCGGTGACG |
|  | **Rev:** atatAGATCTCTTGTACAGCTCGTCCATGCCG |
