# Supplementary material for: The microglia-derived protein Sema4ab attenuates regenerative neurogenesis after spinal cord injury in zebrafish
Source: PLoS Biol. 2026 Jun 18;24(6):e3003865. doi: 10.1371/journal.pbio.3003865 (PMC13309017; doi:10.1371/journal.pbio.3003865)
Supplement: S10 Table — (DOCX) [file pbio.3003865.s023.docx]

| qRT-PCR gene primer list | |
| --- | --- |
| Gene name | **Primer sequence (5’-3’)** |
| *β-actin* | Fw: 5’-CACTGAGGCTCCCCTGAATCCC-3’  Rv: 5’-CGTACAGAGAGAGCACAGCCTGG-3’ |
| *sema4ab* | Fw: 5’-TTCTGTGGCTGGGACCTTTC-3’  Rv: 5’-TGAGGTTCACACTGGAGCAC-3’ |
| *plxnb1a* | Fw: 5’-GAGAGGTGCTCGGTGAACAG-3’  Rv: 5’-GGTTTGCTGGGATCGTGTTG-3’ |
| *plxnb1b* | Fw: 5’-GAGAGCACCGTGGACACAAG-3’  Rv: 5’-CAGGTGGCATGTCAACTATTCAGG-3’ |
| *il1b* | Fw: 5’-ATGGCGAACGTCATCCAAGA-3’  Rv: 5’-GAGACCCGCTGATCTCCTTG-3’ |
| *tnfa* | Fw: 5’-TCACGCTCCATAAGACCCAG-3  Rv: 5’-GATGTGCAAAGACACCTGGC-3’ |
| *il6* | Fw: 5’-CAGCACGGAAAGATGTCTAAC-3’  Rv: 5’-GTTCTTGTCAGCGCTGCCC-3’ |
| *saa* | Fw: 5’-GCCGCTGGAGGTGCAAAG-3’  Rv: 5’-CCTTGCAGAGCCTCCCG-3’ |
| *il11b* | Fw: 5’-CAAATGAACGCaAATGAGTTGAC-3’  Rv: 5’-CTGTCTGTCCGAGcTCTTG-3’ |
| *tgfb1a* | Fw: 5’-GCTGTATGCGCAAGCTTTACA-3’  Rv: 5’-GGACAATTGCTCCACCTTGTG-3 |
| *tgfb3* | Fw: 5’-AAAACGCCAGCAACCTGTTC-3  Rv: 5’-CCTCAACGTCCATCCCTCTG-3’ |
