# Supplementary material for: The microglia-derived protein Sema4ab attenuates regenerative neurogenesis after spinal cord injury in zebrafish
Source: PLoS Biol. 2026 Jun 18;24(6):e3003865. doi: 10.1371/journal.pbio.3003865 (PMC13309017; doi:10.1371/journal.pbio.3003865)
Supplement: Attachment — Submitted filename: _Docampo Seara et al_RevLetter.docx [file pbio.3003865.s035.docx]

**Reviewer #1**

I've reviewed the manuscript by Docampo-Seara submitted to PLoS Biology. The manuscript identifies a role for sema4ab in modulating the injury response environment after spinal cord transection. The authors provide evidence that sema4ab acts (largely) in microglia and modulates regenerative neurogenesis via plexin receptors and by modulating tgfb3 expression from fibroblasts. The work nicely starts to break down the complexity of the injury environment by utilizing scRNA-Seq datasets to identify key players and cell types and their actions and then testing these hypotheses with functional manipulations.  Overall, I quite liked the manuscript. The topic is interesting and the study is well done. I have a few nits to pick that I detail below, but overall, I found the work to be solid, interesting and likely to move the needle in the field in a meaningful way.  Indeed, this was a fun paper to review and is a nice piece of work, of which that the authors should be proud.

**General comments/concerns:**

COMMENT: The introduction could tell a better story to set the reader up to understand the complexity of this study. For example, the concept of balancing regenerative neurogenesis was not well explained, and it was not immediately clear why KOs that increase proliferation and regenerative neurogenesis may do this at the expense of axon regrowth/functional recovery. The introduction reads like a list of fact or bullet points that do not craft a story to highlight the study.

RESPONSE: We thank the reviewer for this comment. In the revised version, we now introduce early on the idea that successful regeneration requires coordination between multiple processes, including ERG proliferation, axon regrowth, and circuit reintegration; and also describe and highlighted the importance of balancing regenerative neurogenesis. We also now specify in the Discussion that a direct connection between regenerative neurogenesis and axonal regrowth is not clear and the two processes could be independent.

COMMENT: I'd also like to see a bit more scholarship in the references provided as these are fairly limited in some instances. For example, the authors should add a citation for the neutrophil infiltration timeline in the first paragraph under "Disruption of sema4ab reduces the number of microglia but does not change numbers of other invading cell types."

RESPONSE: We thank the reviewer for this comment and have changed the section accordingly.

COMMENT: Some of the text could be streamlined and/or edited a bit better. It almost reads in places like the manuscript was written by two people and merged, but not seamlessly. It is an exciting story, but the text, at times, was difficult to wade through and this could detract from the impact of the work.

RESPONSE: We thank the reviewer for this comment and have edited the manuscript to be hopefully more streamlined. Changes are highlighted in the manuscript.

COMMENT: I have two "major" concerns that I'd like to see the Authors address: More comprehensive co-labelling of sema4ab, mcherry, tgfb3, and pdgfrb in the same tissue needs to be done, with quantification, to support the assertions made by the authors.

RESPONSE: We agree with the reviewer and have addressed this point with extensive additional multiplexed HCR-FISH experiments and quantifications. We combined transgenic markers for fibroblasts and macrophages with *tgfb3 in situ* detection in new Fig 8F and with *sema4ab* *in situ* detection in new Fig 8G. These new experiments fully confirmed our previous conclusion that *sema4ab* is mainly expressed by microglia/macrophages and *tgfb3* by fibroblasts.

COMMENT: The authors quantified sema4ab expression in macros/microglia (Fig. 3) and neutrophils but did not quantify plxnb1a/b expression in ERGs (Fig. 4, only images).

RESPONSE: We have now quantified *plxnb1a* and *plxnb1b* expression in ERGs. This confirms our earlier qualitative conclusion that expression hardly changes as a consequence of a lesion (see new Fig 4E).

COMMENT: The authors also do not quantify tgfb3 in fibroblasts (Fig. 8) despite the assertion that "we detect tgfb3 expression only in fibroblasts "in the Discussion.

RESPONSE: We have now added these quantifications in new Fig 8F (see also our response above).

COMMENT: The authors indicate that plxnb1a/b are expressed by ERGs, and their model/assertions indicate that ERGs would be the cell type immediately affected by the sema4ab-plxnb1a/b signaling axis. Thus, the authors should quantify EdU in her4.1:EGFP+ ERGs in addition to the mnx1:EGFP experiments shown in Fig. 5.

RESPONSE: We thank the reviewer for raising this point. We have now performed these quantifications in new Fig 7D together with similar experiments after *tgfb3* disruption (Fig 9C), which has provided interesting additional insight. Differently from the consequences of *sema4ab* KO, where we find an increase in both EdU-labeled-progenitors and motor neurons, we find a depletion of EdU labelled progenitors and an increase in EdU-labeled motor neurons after Plexin receptor disruption (Figs 7C, D). This is consistent with activation of Plexin receptors attenuating neuronal differentiation. In addition, we performed new experiments that show that in the absence of *tgfb3*, EdU labelled progenitors, as well as EdU-labelled motor neurons are depleted, suggesting that *tgfb3* promotes progenitor proliferation (Fig 9C). Hence, the *sema4ab* ko phenotype is consistent with a potential compound phenotype of reduced inhibition of neuronal differentiation (via Plexin-receptors not being activated) and increased progenitor proliferation (via systemic increase of *tgfb3*). This is of course indirect stoichiometric evidence and we cautiously discuss this possibility as an explanation for our signal interactions on p. 22.

**Additional (minor) comments:**

COMMENT: The authors show that fewer microglia infiltrate the injury site with sema4ab KO (Fig. S7). The authors then show that pro-inflammatory markers decrease in the sema4ab KO model at a timepoint "when microglia dominated the injury site" (Fig. 8). They then conclude that there is a "shift from pro-inflammatory to an anti-inflammatory lesion environment in the absence of sema4ab function". The way this part is written (p20) does not account for the decrease in microglia infiltration, which likely contributes to the drop in pro-inflammatory marker expression. It would be helpful if the authors could incorporate the macrophage/microglia data panels from Fig. S7 into a main figure and discuss these results alongside the cytokine expression data, rather than under a separate subheading.

RESPONSE: We thank the reviewer for this point. The BDM/microglia quantification previously shown in S7 Fig has now been moved into the main figures and is presented integrated with the cytokine expression (new Fig 8). We now acknowledge that reduced cytokine abundance can be due to both reduced expression by microglia and reduced number of microglia in the Results on p. 16.

COMMENT: SB431542 inhibits multiple ALK receptors and can impact other tgfb family members (ex., activin, BMP, TGFb1/2). The results section discussing the SB431542 experiments should be worded more conservatively to reflect this and not link the findings directly with tgfb3.

RESPONSE: We agree and explicitly state the potential multiple actions of the drug in the limitation paragraph on p. 25 now: “Third, despite the pharmacological inhibition of *tgfb* signaling (SB431542) aligns with the results obtained using somatic mutants for *tgfb3*, the drug can affect multiple ALK receptors, and therefore the conclusions obtained from this experiment should be considered cautiously.”

COMMENT: I think it would be helpful to include an explicit paragraph about study limitations (systemic KOs, etc.) in the Discussion, to balance what is unknown with what advances are made here.

RESPONSE: We added a limitation paragraph in the discussion on p. 25 to address this and other limitations of the study.

COMMENT: I would like to see clarification regarding the "immune cell dataset" mentioned in the second paragraph of the results section for supplemental figure 1A. The authors state that immune cells were enriched by "FACS sorting for GFP expression under the mpeg1.1 regulatory sequences." Do neutrophils express mpeg1.1? Maybe these are mpeg1.1+ neutrophil-like cells, but should be noted as such unless the authors are certain they are neutrophils.

RESPONSE: We have added an explanatory sentence to the Results on p. 5: “This strongly enriched BDMs and microglia, but the sample also contained neutrophils and other cell types, possibly because *mpeg1.1* was also expressed in other cell types...”.

COMMENT: Nothing really to do here unless this is interesting enough to mention in the Discussion…..the tgfb3 expression pattern in supplemental Fig. 9A appears to be less patterned for the gSema4ab group relative to gControls. The gControls have a pattern that seems confined by somite. Are the regenerating motor neurons in the sema4ab mutants are dysfunctional? is there any aberrant sprouting, etc.? Retrograde tracing or some assessment of function would be interesting to do here. Maybe this is nothing, and this is a bit out of my expertise, but it seemed interesting to me.

RESPONSE: We thank the reviewer for this comment. We re-examined the pattern of *tgfb3* and found that any differences were non-systematic and within the fish-to-fish variability. We therefore have chosen more representative images for both conditions to avoid this concern (S9A Fig).

The reviewer also mentions integration, sprouting and functionality of newly generated motor neurons, which is a very interesting topic on its own. However, in the present study, motor neurons are only used as indicators of neurogenesis. Analyses of integration would require sophisticated tools that unfortunately go beyond the scope of this study. We explicitly address possible connections between neurogenesis and maladaptive circuitry repair, as well as the possibility of independent action of *sema4ab* in axon growth vs neurogenesis in a new paragraph in the Discussion: “We show that *sema4ab* promotes axonal growth…”.

**Reviewer #2**

In this study, Docampo-Seara and colleagues investigated the role of sema4ab in neurogenesis following larval zebrafish spinal cord injury (SCI). Using single-cell RNA-sequencing (scRNA-seq) and in situ hybridization, the authors show that sema4ab is primarily expressed by and likely secreted by microglia and blood-derived macrophages. Sema4ab perturbation using CRISPR-mediated deletion increases motor neuron production but simultaneously hinders functional recovery following spinal cord injury. Plxnb1a and plxnb1b, two sema4ab receptors primarily expressed in ependymo-radial glial cells (ERGs), produced a similar effect on neurogenesis when both perturbed at the same time. Sema4ab perturbation altered a broad range of other signalling pathways after SCI. Most notably, sema4ab perturbation increased expression of the pro-neurogenesis factor tgfb3 in fibroblasts and alters the composition of the inflammatory milieu. The authors propose that sema4ab has both a direct and indirect effect on neurogenesis. Sema4ab can directly inhibit neuronal differentiation by binding the plxnb1a/b receptors on ERGs and can also alter the composition of the inflammatory milieu to decrease tgfb3 expression in fibroblasts.

Overall, this study presents a novel negative regulatory mechanism of neurogenesis that may help maintain progenitor pool sizes after SCI. It is intriguing that sema4ab acts as a microglia-derived signal to regulate neural progenitor pools and fibroblasts behavior during regeneration. The data are compelling and will be of interest for the field - especially for those involved in cell crosstalk during tissue regeneration. However, some concerns need to be addressed before the paper can be considered for publishing in Plos Biology.

**Main Comments:**

COMMENT: 1.      In their scRNA-seq data, the authors claim MM#3 and MM#4 likely correspond to blood-derived macrophages (BDM), given their low expression of microglia markers and their high expression of BDM markers (Figure 1F). Although I agree that MM#3 and MM#4 exhibit much lower expression of microglia markers, they appear to have the same expression of BDM markers when compared to MM#1 and MM#2. Some of the BDM markers, namely grn1 and irf8, appear more highly expressed in MM#1 and MM#2 compared to MM#3 and MM#4. Other markers like siglec15l, ccl19a.1, and hepacam2 show very low expression percentages, and therefore may not be robustly indicative of BDM identity. The authors should clarify how the BDM markers were selected and how these expression patterns support the assignment of MM#3 and MM#4 as BDMs.

RESPONSE: We thank the reviewer for highlighting this aspect of the scRNA-seq data. The marker genes used to discriminate microglia from BDMs were selected based on a set of genes by Zambusi et al., 2022, who analysed microglia following traumatic brain injury, as previously indicated in the manuscript. While the majority of these markers and some additional markers we curated support the distinction between microglia and BDMs, some markers are apparently not conserved between CNS regions and/or developmental stages. We more clearly address this caveat now on p. 7: “However, some of the markers did not fully agree with the assigned cell identities. This may be due to regional heterogeneity within the CNS and/or differences across zebrafish developmental stages that affect marker expression by BDMs and microglia.”

COMMENT: 2.      In Fig. 5D, the sema4ab-/-; hsp70:sema4ab panel shows more overall EdU labeling than the WT panel, which is not reflected in the quantification. Please clarify whether the image is representative and if non motor-neuron EdU labeling contributes to this discrepancy.

RESPONSE: We thank the reviewer for pointing this out. The difference is indeed a non-systematic consequence of inter-animal variation and we have replaced the image in question with are more representative one.

COMMENT: 3.      In figure 5D, does heat shock alone, in absence of injury, increases neurogenesis?

RESPONSE: We have addressed this question experimentally in new Fig 5E. Over-expressing *sema4ab* in unlesioned animals attenuates ongoing developmental generation of motor neurons by 32%. Hence, an injury signal is not needed for the negative action of *sema4ab* on neurogenesis. This is consistent with expression of Plexin receptors in spinal progenitor cells in unlesioned animals. We now give this interpretation on p. 12-13:” We therefore over-expressed *sema4ab* in unlesioned wildtype animals…”

COMMENT: 4.      Do other semaphorins show injury-induced regulation in scRNAseq dataset?

RESPONSE: We have analysed the scRNA-seq dataset for other semaphorin family members in BDMs and microglia. Indeed, *sema4ab* transcripts show that highest abundance and upregulation after lesion of all detectable *sema* genes. We have added a dot plot in S1E Fig to the manuscript and describe the data ion the Results on p. 5: ”Interestingly, of all the semaphorins detectably expressed in this dataset, *sema4ab* was markedly more strongly expressed and also upregulated after lesion, compared to all other family members, including the paralog *sema4aa* (S1E Fig).”

COMMENT:

5.      The model proposes that microglial sema4ab indirectly suppresses fibroblast-derived tgfb3 by altering the inflammatory milieu. While the direct sema4ab-Plxnb1a/b effects on ERGs are supported by ligand-receptor expression, this indirect mechanism is less directly tested. Using the scRNA-seq data, can the authors assess whether fibroblasts express receptors for cytokines altered in sema4ab mutants (il1b, tnfa, il6, saa, il11b)? This analysis would strengthen the proposed link between microglial signaling changes and fibroblast tgfb3 regulation.

RESPONSE: We thank the reviewer for this suggestion. We have now included feature plots for the main receptors of the mentioned cytokines and highlighted the expression in fibroblasts (new S10F Fig). This indicates multiple potential signal/receptor interactions from immune cells to fibroblasts and we mention this on p. 16 in the Results and on p. 24 in the Discussion. In addition, we functionally tested Il1b signalling and Tnf signalling (see response to comment 8).

COMMENT: 6.      In Fig. 5 and 6, the observation that enhanced neurogenesis coincides with impaired axon regrowth and swimming recovery is intriguing. Additional discussion clarifying how excessive neurogenesis may disrupt lesion organization, axon guidance, or circuit integration would help reconcile these findings. Is there evidence that newly generated neurons in sema4ab mutants are mis-specified, improperly integrated, or failing to extend axons?

RESPONSE: Unfortunately, to assess the differentiation of newly generated neurons and possible alterations of that in the absence of *sema4ab* would require quantitative genetic lineage tracing models that we cannot easily establish for this study. In principle, excessive neurogenesis can perturb system function. For example, excessive neurogenesis in the hippocampus after stroke impairs memory and can be rescued by experimentally reducing neurogenesis (Cuartero *et al*, 2019). This could be similar in our case. Alternatively, Sema4ab could act directly on axon guidance. We mention these considerations in a new paragraph in the Discussion on p. 24: “We show that *sema4ab* promotes axonal growth…”.

COMMENT: 7.      The authors show that sema4ab disruption leads to both reduced microglial proliferation/number and broad suppression of inflammatory gene programs. However, it remains unclear whether downstream effects on fibroblast tgfb3 expression and neurogenesis are driven primarily by altered microglial activation state, reduced microglial abundance, or a combination of both.

RESPONSE: We thank the reviewer for this interesting question. We now demonstrate that individual microglial cells show a relatively modest reduction in *il1b* expression in semi-quantitative HCR (11%). Given the relatively modest decrease observed, this result suggest that a combination of altered microglial activation state and microglial abundance shapes the injury milieu and may influence fibroblast behaviour after *sema4ab* disruption. This new experiment has been incorporated into S8D Fig, and the findings are now discussed in the revised manuscript on p. 16: “Overall, a combination of a reduction in microglia number, activation state, and influence on other lesion site cells led to a shift…”

COMMENT: 8.      While the authors show increased fibroblast-derived tgfb3 expression in sema4ab mutants (Fig. 2F; Fig. 8D-F), it remains unclear whether this reflects direct microglia-fibroblast signaling or an indirect consequence of the altered inflammatory milieu. This could be addressed by testing whether tgfb3 upregulation persists following microglial depletion or suppression, and/or by cytokine rescue experiments to determine whether inflammatory cues are sufficient to drive fibroblasts tgfb3 upregulation Clarifying whether microglia are necessary intermediates in this pathway would strengthen the proposed.

RESPONSE: To address this interesting point, we performed *tgfb3* qRT-PCR analyses in *csf1ra/csf1rb* double mutants, which shows a microglia-specific depletion. This showed an increase in *tgfb3* expression in the absence of microglia, similar to disrupting *sema4ab.* Hence, microglia are necessary for *tgfb3* control. In addition, we tested candidate pathways (*il1b, tnfrsf1a*). These had negative results, such that the exact mechanism by which microglia act on fibroblast tgfb3 expression remains elusive. We added these additional experiments as new S10 Fig to the manuscript and describe them on p. 17: “To determine whether microglia were indeed responsible…”

COMMENT: 9.      Seven fibroblast clusters are identified by scRNA-seq, yet tgfb3 regulation is discussed largely at the population level. Which fibroblast subtypes contribute most strongly to tgfb3 upregulation?

RESPONSE:

We have now performed sub-clustering of the fibroblasts, identifying specific *tgfb3+* populations (S11 Fig). However, we could not see increased expression in the *tgfb3+* fibroblast clusters in scRNA-seq. Therefore, determining whether a specific sub-cluster of fibroblasts is responsible for the increased expression of *tgfb3* needs to be addressed in future research. We now give the sub-cluster analysis and this explanation on p. 18 in the Results.

COMMENT 9 continued: Are tgfb3-expressing fibroblasts spatially enriched near ERGs or axon growth zones, supporting a local paracrine effect?

RESPONSE: We added histology images showing that *tgfb3+* fibroblasts are present in the lesion site at a spinal cord level, where axons grow, and on the surface of the spinal stumps, in which the ERGs are located (S11E Fig). Live imaging indicates contact of axons with fibroblasts in the lesion and close proximity of fibroblasts and ERGs in S11F and S11G Figs.

**Minor Comments:**

COMMENT: 1.      On page 43, replace "agvg_log2FC" with "avg_log2FC"

RESPONSE: We corrected this mistake.

COMMENT: 2.      In the methods section under Quality Control and Doublet Removal, the authors define one of their selection criteria was "percent.mt > 20 %". Did the authors intend to put "percent.mt < 20 %"? High mitochondrial mRNA content typically indicates a cell is dying.

RESPONSE: Thank you for pointing this out. This was a typo and is now corrected.

COMMENT: 3.      The qRT-PCR analyses reflect whole-lesion tissue. Where possible, the authors should clarify how these bulk changes relate to cell-type-specific transcriptional changes observed by HCR-FISH and scRNA-seq.

RESPONSE: We added this aspect along the result section.

COMMENT: 4.      The modest reduction in microglial number shown in Suppl. Fig. 7E-F raises the question of whether cell density effects could contribute to altered signalling; this should be discussed.

RESPONSE: This is now included in main Fig 8A (see response to main Comment 7)

COMMENT: 5.      The opposing regulation of tgfb1a and tgfb3 is intriguing. Do these ligands have distinct or antagonistic roles in this context?

RESPONSE: Tgfb1 and Tgfb3 may have opposing roles in terms of scar resolution; Tgfb1 has been described as pro-fibrotic, whereas Tgfb3 has been associated with scar resolution and scar-less healing in wound injury models (Lichtman et al, 2016). The observed reduction of *tgfb1a* expression in our spinal cord injury model and simultaneous upregulation of *tgfb3* after *sema4ab* disruption may thus lead to a more pro-regenerative environment. We now mention this in the discussion on p. 24: “Interestingly, we observed reduced expression of *tgfb1…”*.
