# Supplementary material for: The microglia-derived protein Sema4ab attenuates regenerative neurogenesis after spinal cord injury in zebrafish
Source: PLoS Biol. 2026 Jun 18;24(6):e3003865. doi: 10.1371/journal.pbio.3003865 (PMC13309017; doi:10.1371/journal.pbio.3003865)
Supplement: Peer review history

# Editor report: Decision Letter 0

**DOI:** 10.1371/journal.pbio.3003865.r001

**Authors:** Lucas Smith

6 Nov 2025

***HAVE YOU ASSIGNED THE AE? IF NOT CANCEL THIS DECISION AND GO BACK!***

***REMEMBER TO CLOSE ALL REDUNDANT AE DISCUSSIONS***

Dear Dr Docampo Seara,

Thank you for submitting your manuscript entitled "Microglia attenuate regenerative neurogenesis via sema4ab after spinal cord injury in zebrafish" for consideration as a Research Article by PLOS Biology.

Your manuscript has now been evaluated by the PLOS Biology editorial staff [as well as by an academic editor with relevant expertise *EDIT AS APPLICABLE*] and I am writing to let you know that we would like to send your submission out for external peer review.

However, before we can send your manuscript to reviewers, we need you to complete your submission by providing the metadata that is required for full assessment. To this end, please login to Editorial Manager where you will find the paper in the 'Submissions Needing Revisions' folder on your homepage. Please click 'Revise Submission' from the Action Links and complete all additional questions in the submission questionnaire.

Once your full submission is complete, your paper will undergo a series of checks in preparation for peer review. After your manuscript has passed the checks it will be sent out for review. To provide the metadata for your submission, please Login to Editorial Manager (https://www.editorialmanager.com/pbiology) within two working days, i.e. by Nov 10 2025 11:59PM.

If your manuscript has been previously peer-reviewed at another journal, PLOS Biology is willing to work with those reviews in order to avoid re-starting the process. Submission of the previous reviews is entirely optional and our ability to use them effectively will depend on the willingness of the previous journal to confirm the content of the reports and share the reviewer identities. Please note that we reserve the right to invite additional reviewers if we consider that additional/independent reviewers are needed, although we aim to avoid this as far as possible. In our experience, working with previous reviews does save time.

If you would like us to consider previous reviewer reports, please edit your cover letter to let us know and include the name of the journal where the work was previously considered and the manuscript ID it was given. In addition, please upload a response to the reviews as a 'Prior Peer Review' file type, which should include the reports in full and a point-by-point reply detailing how you have or plan to address the reviewers' concerns.

During the process of completing your manuscript submission, you will be invited to opt-in to posting your pre-review manuscript as a bioRxiv preprint. Visit http://journals.plos.org/plosbiology/s/preprints for full details. If you consent to posting your current manuscript as a preprint, please upload a single Preprint PDF.

Feel free to email us at plosbiology@plos.org if you have any queries relating to your submission.

Kind regards,

Lucas

Lucas Smith, Ph.D.

Senior Editor

PLOS Biology

lsmith@plos.org

# Peer review: Decision Letter 1

**DOI:** 10.1371/journal.pbio.3003865.r002

**Authors:** Lucas Smith, Lucas Smith

23 Jan 2026

Dear Dr Docampo Seara,

Thank you again for your patience while your manuscript "Microglia attenuate regenerative neurogenesis via sema4ab after spinal cord injury in zebrafish" was peer-reviewed at PLOS Biology. Your manuscript has now been evaluated by the PLOS Biology editors, an Academic Editor with relevant expertise, and by several independent reviewers. In light of the reviews, which you will find at the end of this email, we would like to invite you to revise the work to thoroughly address the reviewers' reports.

As you will see below, the reviewers report that the study is interesting and generally well done. However both reviewers have provided suggestions to strengthen the study further, and we think their comments will need to be thoroughly addressed before we can consider your study for publication. While many of the reviewer comments can be addressed with textual changes, we think you should also add the new analyses and experiments proposed by Reviewer 2. We do note that reviewer 2's comment 8 may be challenging to address. So while we think that it would be important for you to try the approach proposed by this reviewer - if for some reason that does not work and additional troubleshooting would delay publication, then we are open to you addressing that point with text additions.

As a last editorial note, the Academic Editor has suggested that you please add a clearly-labeled limitations paragraph to the discussion. In that paragraph, we think you can address any reviewer comments that are not experimental addressable and other limitations they identify.

Given the extent of revision needed, we cannot make a decision about publication until we have seen the revised manuscript and your response to the reviewers' comments. Your revised manuscript is likely to be sent for further evaluation by all or a subset of the reviewers.

In addition to these revisions, you will need to complete some formatting changes, which you will receive in a follow up email. A member of our team will be in touch with a set of requests shortly.

We expect to receive your revised manuscript within 3 months. Please email us (plosbiology@plos.org) if you have any questions or concerns, or would like to request an extension.

At this stage, your manuscript remains formally under active consideration at our journal; please notify us by email if you do not intend to submit a revision so that we may withdraw it.

**IMPORTANT - SUBMITTING YOUR REVISION**

Your revisions should address the specific points made by each reviewer. Please submit the following files along with your revised manuscript:

1. A 'Response to Reviewers' file - this should detail your responses to the editorial requests, present a point-by-point response to all of the reviewers' comments, and indicate the changes made to the manuscript.

*NOTE: In your point-by-point response to the reviewers, please provide the full context of each review. Do not selectively quote paragraphs or sentences to reply to. The entire set of reviewer comments should be present in full and each specific point should be responded to individually, point by point.

You should also cite any additional relevant literature that has been published since the original submission and mention any additional citations in your response.

2. In addition to a clean copy of the manuscript, please also upload a 'track-changes' version of your manuscript that specifies the edits made. This should be uploaded as a "Revised Article with Changes Highlighted" file type.

*Re-submission Checklist*

When you are ready to resubmit your revised manuscript, please refer to this re-submission checklist: https://plos.io/Biology_Checklist

To submit a revised version of your manuscript, please go to https://www.editorialmanager.com/pbiology/ and log in as an Author. Click the link labelled 'Submissions Needing Revision' where you will find your submission record.

Please make sure to read the following important policies and guidelines while preparing your revision:

*Published Peer Review*

Please note while forming your response, if your article is accepted, you may have the opportunity to make the peer review history publicly available. The record will include editor decision letters (with reviews) and your responses to reviewer comments. If eligible, we will contact you to opt in or out. Please see here for more details:

https://blogs.plos.org/plos/2019/05/plos-journals-now-open-for-published-peer-review/

*PLOS Data Policy*

Please note that as a condition of publication PLOS' data policy (http://journals.plos.org/plosbiology/s/data-availability) requires that you make available all data used to draw the conclusions arrived at in your manuscript. If you have not already done so, you must include any data used in your manuscript either in appropriate repositories, within the body of the manuscript, or as supporting information (N.B. this includes any numerical values that were used to generate graphs, histograms etc.). For an example see here: http://www.plosbiology.org/article/info%3Adoi%2F10.1371%2Fjournal.pbio.1001908#s5

*Blot and Gel Data Policy*

We require the original, uncropped and minimally adjusted images supporting all blot and gel results reported in an article's figures or Supporting Information files. We will require these files before a manuscript can be accepted so please prepare them now, if you have not already uploaded them. Please carefully read our guidelines for how to prepare and upload this data: https://journals.plos.org/plosbiology/s/figures#loc-blot-and-gel-reporting-requirements

*Protocols deposition*

To enhance the reproducibility of your results, we recommend that if applicable you deposit your laboratory protocols in protocols.io, where a protocol can be assigned its own identifier (DOI) such that it can be cited independently in the future. Additionally, PLOS ONE offers an option for publishing peer-reviewed Lab Protocol articles, which describe protocols hosted on protocols.io. Read more information on sharing protocols at https://plos.org/protocols?utm_medium=editorial-email&utm_source=authorletters&utm_campaign=protocols

Thank you again for your submission to our journal. We hope that our editorial process has been constructive thus far, and we welcome your feedback at any time. Please don't hesitate to contact us if you have any questions or comments.

Sincerely,

Luke

Lucas Smith, Ph.D.

Senior Editor

PLOS Biology

lsmith@plos.org

------------------------------------

REVIEWS:

Reviewer #1: I've reviewed the manuscript by Docampo-Seara submitted to PLoS Biology. The manuscript identifies a role for sema4ab in modulating the injury response environment after spinal cord transection. The authors provide evidence that sema4ab acts (largely) in microglia and modulates regenerative neurogenesis via plexin receptors and by modulating tgfb3 expression from fibroblasts. The work nicely starts to break down the complexity of the injury environment by utilizing scRNA-Seq datasets to identify key players and cell types and their actions and then testing these hypotheses with functional manipulations. Overall, I quite liked the manuscript. The topic is interesting and the study is well done. I have a few nits to pick that I detail below, but overall, I found the work to be solid, interesting and likely to move the needle in the field in a meaningful way. Indeed, this was a fun paper to review and is a nice piece of work, of which that the authors should be proud.

General comments/concerns:

The introduction could tell a better story to set the reader up to understand the complexity of this study. For example, the concept of balancing regenerative neurogenesis was not well explained, and it was not immediately clear why KOs that increase proliferation and regenerative neurogenesis may do this at the expense of axon regrowth/functional recovery. The introduction reads like a list of fact or bullet points that do not craft a story to highlight the study.

I'd also like to see a bit more scholarship in the references provided as these are fairly limited in some instances. For example, the authors should add a citation for the neutrophil infiltration timeline in the first paragraph under "Disruption of sema4ab reduces the number of microglia but does not change numbers of other invading cell types."

Some of the text could be streamlined and/or edited a bit better. It almost reads in places like the manuscript was written by two people and merged, but not seamlessly. It is an exciting story, but the text, at times, was difficult to wade through and this could detract from the impact of the work.

I have two "major" concerns that I'd like to see the Authors address:

More comprehensive co-labeling of sema4ab, mcherry, tgfb3, and pdgfrb in the same tissue needs to be done, with quantification, to support the assertions made by the authors. The authors quantified sema4ab expression in macros/microglia (Fig. 3) and neutrophils but did not quantify plxnb1a/b expression in ERGs (Fig. 4, only images). The authors also do not quantify tgfb3 in fibroblasts (Fig. 8) despite the assertion that "we detect tgfb3 expression only in fibroblasts" in the Discussion.

The authors indicate that plxnb1a/b are expressed by ERGs, and their model/assertions indicate that ERGs would be the cell type immediately affected by the sema4ab-plxnb1a/b signaling axis. Thus, the authors should quantify EdU in her4.1:EGFP+ ERGs in addition to the mnx1:EGFP experiments shown in Fig. 5.

Additional (minor) comments:

The authors show that fewer microglia infiltrate the injury site with sema4ab KO (Fig. S7). The authors then show that pro-inflammatory markers decrease in the sema4ab KO model at a timepoint "when microglia dominated the injury site" (Fig. 8). They then conclude that there is a "shift from pro-inflammatory to an anti-inflammatory lesion environment in the absence of sema4ab function". The way this part is written (p20) does not account for the decrease in microglia infiltration, which likely contributes to the drop in pro-inflammatory marker expression. It would be helpful if the authors could incorporate the macrophage/microglia data panels from Fig. S7 into a main figure and discuss these results alongside the cytokine expression data, rather than under a separate subheading.

SB431542 inhibits multiple ALK receptors and can impact other tgfb family members (ex., activin, BMP, TGFb1/2). The results section discussing the SB431542 experiments should be worded more conservatively to reflect this and not link the findings directly with tgfb3.

I think it would be helpful to include an explicit paragraph about study limitations (systemic KOs, etc.) in the Discussion, to balance what is unknown with what advances are made here.

I would like to see clarification regarding the "immune cell dataset" mentioned in the second paragraph of the results section for supplemental figure 1A. The authors state that immune cells were enriched by "FACS sorting for GFP expression under the mpeg1.1 regulatory sequences." Do neutrophils express mpeg1.1? Maybe these are mpeg1.1+ neutrophil-like cells, but should be noted as such unless the authors are certain they are neutrophils.

Nothing really to do here unless this is interesting enough to mention in the Discussion…..the tgfb3 expression pattern in supplemental Fig. 9A appears to be less patterned for the gSema4ab group relative to gControls. The gControls have a pattern that seems confined by somite. Are the regenerating motor neurons in the sema4ab mutants are dysfunctional? is there any aberrant sprouting, etc.? Retrograde tracing or some assessment of function would be interesting to do here. Maybe this is nothing, and this is a bit out of my expertise, but it seemed interesting to me.

Reviewer #2: In this study, Decampo-Seara and colleagues investigated the role of sema4ab in neurogenesis following larval zebrafish spinal cord injury (SCI). Using single-cell RNA-sequencing (scRNA-seq) and in situ hybridization, the authors show that sema4ab is primarily expressed by and likely secreted by microglia and blood-derived macrophages. Sema4ab perturbation using CRISPR-mediated deletion increases motor neuron production but simultaneously hinders functional recovery following spinal cord injury. Plxnb1a and plxnb1b, two sema4ab receptors primarily expressed in ependymo-radial glial cells (ERGs), produced a similar effect on neurogenesis when both perturbed at the same time. Sema4ab perturbation altered a broad range of other signaling pathways after SCI. Most notably, sema4ab perturbation increased expression of the pro-neurogenesis factor tgfb3 in fibroblasts and alters the composition of the inflammatory milieu. The authors propose that sema4ab has both a direct and indirect effect on neurogenesis. Sema4ab can directly inhibit neuronal differentiation by binding the plxnb1a/b receptors on ERGs and can also alter the composition of the inflammatory milieu to decrease tgfb3 expression in fibroblasts.

Overall, this study presents a novel negative regulatory mechanism of neurogenesis that may help maintain progenitor pool sizes after SCI. It is intriguing that sema4ab acts as a microglia-derived signal to regulate neural progenitor pools and fibroblasts behavior during regeneration. The data are compelling and will be of interest for the field - especially for those involved in cell crosstalk during tissue regeneration. However, some concerns need to be addressed before the paper can be considered for publishing in Plos Biology.

Main Comments:

1. In their scRNA-seq data, the authors claim MM#3 and MM#4 likely correspond to blood-derived macrophages (BDM), given their low expression of microglia markers and their high expression of BDM markers (Figure 1F). Although I agree that MM#3 and MM#4 exhibit much lower expression of microglia markers, they appear to have the same expression of BDM markers when compared to MM#1 and MM#2. Some of the BDM markers, namely grn1 and irf8, appear more highly expressed in MM#1 and MM#2 compared to MM#3 and MM#4. Other markers like siglec15l, ccl19a.1, and hepacam2 show very low expression percentages, and therefore may not be robustly indicative of BDM identity. The authors should clarify how the BDM markers were selected and how these expression patterns support the assignment of MM#3 and MM#4 as BDMs.

2. In Fig. 5D, the sema4ab-/-; hsp70:sema4ab panel shows more overall EdU labeling than the WT panel, which is not reflected in the quantification. Please clarify whether the image is representative and if non motor-neuron EdU labeling contributes to this discrepancy.

3. In figure 5D, does heat shock alone, in absence of injury, increases neurogenesis?

4. Do other semaphorins show injury-induced regulation in scRNAseq dataset?

5. The model proposes that microglial sema4ab indirectly suppresses fibroblast-derived tgfb3 by altering the inflammatory milieu. While the direct sema4ab-Plxnb1a/b effects on ERGs are supported by ligand-receptor expression, this indirect mechanism is less directly tested. Using the scRNA-seq data, can the authors assess whether fibroblasts express receptors for cytokines altered in sema4ab mutants (il1b, tnfa, il6, saa, il11b)? This analysis would strengthen the proposed link between microglial signaling changes and fibroblast tgfb3 regulation.

6. In Fig. 5 and 6, the observation that enhanced neurogenesis coincides with impaired axon regrowth and swimming recovery is intriguing. Additional discussion clarifying how excessive neurogenesis may disrupt lesion organization, axon guidance, or circuit integration would help reconcile these findings. Is there evidence that newly generated neurons in sema4ab mutants are mis-specified, improperly integrated, or failing to extend axons?

7. The authors show that sema4ab disruption leads to both reduced microglial proliferation/number and broad suppression of inflammatory gene programs. However, it remains unclear whether downstream effects on fibroblast tgfb3 expression and neurogenesis are driven primarily by altered microglial activation state, reduced microglial abundance, or a combination of both.

8. While the authors show increased fibroblast-derived tgfb3 expression in sema4ab mutants (Fig. 2F; Fig. 8D-F), it remains unclear whether this reflects direct microglia-fibroblast signaling or an indirect consequence of the altered inflammatory milieu. This could be addressed by testing whether tgfb3 upregulation persists following microglial depletion or suppression, and/or by cytokine rescue experiments to determine whether inflammatory cues are sufficient to drive fibroblasts tgfb3 upregulation Clarifying whether microglia are necessary intermediates in this pathway would strengthen the proposed

9. Seven fibroblast clusters are identified by scRNA-seq, yet tgfb3 regulation is discussed largely at the population level. Which fibroblast subtypes contribute most strongly to tgfb3 upregulation? Are tgfb3-expressing fibroblasts spatially enriched near ERGs or axon growth zones, supporting a local paracrine effect?

Minor Comments:

1. On page 43, replace "agvg_log2FC" with "avg_log2FC"

2. In the methods section under Quality Control and Doublet Removal, the authors define one of their selection criteria was "percent.mt > 20 %". Did the authors intend to put "percent.mt < 20 %"? High mitochondrial mRNA content typically indicates a cell is dying.

3. The qRT-PCR analyses reflect whole-lesion tissue. Where possible, the authors should clarify how these bulk changes relate to cell-type-specific transcriptional changes observed by HCR-FISH and scRNA-seq.

4. The modest reduction in microglial number shown in Suppl. Fig. 7E-F raises the question of whether cell density effects could contribute to altered signaling; this should be discussed.

5. The opposing regulation of tgfb1a and tgfb3 is intriguing. Do these ligands have distinct or antagonistic roles in this context?

# Author comment: Author response to Decision Letter 2

**DOI:** 10.1371/journal.pbio.3003865.r003

20 Apr 2026

Attachment Submitted filename: _Docampo Seara et al_RevLetter.docx

# Editor report: Decision Letter 2

**DOI:** 10.1371/journal.pbio.3003865.r004

**Authors:** Lucas Smith, Lucas Smith, Lucas Smith

8 May 2026

Dear Dr Docampo Seara,

Thank you for your patience while we considered your revised manuscript "Microglia attenuate regenerative neurogenesis via sema4ab after spinal cord injury in zebrafish" for publication as a Research Article at PLOS Biology. This revised version of your manuscript has been evaluated by the PLOS Biology editors and the Academic Editor.

The Academic Editor is largely satisfied with the revision, although s/he has a number of last minor points which we think should be addressed in a last revision before we can accept your study. These are included below my signature. We are likely to accept this manuscript, provided you satisfactorily address the remaining points.

**IMPORTANT: Please also make sure to address the following data and other policy-related requests:

1) TITLE: we would like to propose a tweak to the title. If you agree, we suggest you change it to "The microglia-derived protein sema4ab attenuates regenerative neurogenesis after spinal cord injury in zebrafish"

2) ETHICS STATEMENT: Thank you for providing an ethics statement detailing the protocol numbers and approval body for your animal care and use protocol. In addition to these details, we need you to explicitely state to what national, or international guidelines your protocol adhered to.

3) DATA: Thank you for providing your gene expression data on GEO. I did not see a reviewer token to access this data (sorry if I missed it!). Can you provide me one? Please note, this dataset will need to be made public at the time of publication.

4) DATA: Thanks also for providing the other underlying data for your paper as supplementary excel files. Please add a sentence to each figure legend (including supplemental) pointing readers to the relevant data file, where the underling data can be found.

5) BLOTS AND GELS: Please note that we require the original, uncropped and minimally adjusted images supporting all blot and gel results reported in an article's figures or Supporting Information files. Please provide a supplementary file, titled 'S1_raw_images' containing annotated versions of the original uncropped and unadjusted images related to the blots and gels presented in your study. Please carefully read our guidelines for how to prepare and upload this data: https://journals.plos.org/plosbiology/s/figures#loc-blot-and-gel-reporting-requirements

6) CODE: Per journal policy, if you have generated any custom code during the course of this investigation, please make it available without restrictions. Please ensure that the code is sufficiently well documented and reusable, and that your Data Statement in the Editorial Manager submission system accurately describes where your code can be found. More information on our Code Policy, what and how to share can be found here: https://journals.plos.org/plosbiology/s/code-availability

Please note that we cannot accept sole deposition of code in GitHub, as this could be changed after publication. However, you can archive this version of your publicly available GitHub code to Zenodo. Once you do this, it will generate a DOI number, which you will need to provide in the Data Accessibility Statement (you are welcome to also provide the GitHub access information). See the process for doing this here: https://docs.github.com/en/repositories/archiving-a-github-repository/referencing-and-citing-content

As you address these items, please take this last chance to review your reference list to ensure that it is complete and correct. If you have cited papers that have been retracted, please include the rationale for doing so in the manuscript text, or remove these references and replace them with relevant current references. Any changes to the reference list should be mentioned in the cover letter that accompanies your revised manuscript.

In addition to these revisions, you may need to complete some formatting changes, which you will receive in a follow up email. A member of our team will be in touch with a set of requests shortly. If you do not receive a separate email within a few days, please assume that checks have been completed, and no additional changes are required.

We expect to receive your revised manuscript within two weeks.

To submit your revision, please go to https://www.editorialmanager.com/pbiology/ and log in as an Author. Click the link labelled 'Submissions Needing Revision' to find your submission record. Your revised submission must include the following:

- a cover letter that should detail your responses to any editorial requests, if applicable, and whether changes have been made to the reference list

- a Response to Reviewers file that provides a detailed response to the reviewers' comments (if applicable, if not applicable please do not delete your existing 'Response to Reviewers' file.)

- a track-changes file indicating any changes that you have made to the manuscript.

NOTE: If Supporting Information files are included with your article, note that these are not copyedited and will be published as they are submitted. Please ensure that these files are legible and of high quality (at least 300 dpi) in an easily accessible file format. For this reason, please be aware that any references listed in an SI file will not be indexed. For more information, see our Supporting Information guidelines:

https://journals.plos.org/plosbiology/s/supporting-information

*Published Peer Review History*

Please note that you may have the opportunity to make the peer review history publicly available. The record will include editor decision letters (with reviews) and your responses to reviewer comments. If eligible, we will contact you to opt in or out. Please see here for more details:

https://plos.org/published-peer-review-history/

*Press*

Should you, your institution's press office or the journal office choose to press release your paper, please ensure you have opted out of Early Article Posting on the submission form. We ask that you notify us as soon as possible if you or your institution is planning to press release the article.

*Protocols deposition*

To enhance the reproducibility of your results, we recommend that if applicable you deposit your laboratory protocols in protocols.io, where a protocol can be assigned its own identifier (DOI) such that it can be cited independently in the future. Additionally, PLOS ONE offers an option for publishing peer-reviewed Lab Protocol articles, which describe protocols hosted on protocols.io. Read more information on sharing protocols at https://plos.org/protocols?utm_medium=editorial-email&utm_source=authorletters&utm_campaign=protocols

Please do not hesitate to contact me should you have any questions.

Sincerely,

Luke

Lucas Smith, Ph.D.

Senior Editor

lsmith@plos.org

PLOS Biology

------------------------------------------------------------------------

COMMENTS FROM THE ACADEMIC EDITOR (which I have slightly edited):

After reading the revised manuscript, I think it would be ready for acceptance after some minor text changes. Mainly, I think it would be valuable for the authors to revise the text to be cautious about placing sema4ab function in microglia given that they never tested cell-autonomy of sema4ab in microglia. Here are some examples, but it would be helpful for the authors to thoroughly review the paper for other instances.

-Cell-autonomy text changes.

In the introduction the authors state "Hence, sema4ab+ microglia" which suggests cell-autonomy was tested. Please change that language to more conservative text. For example, "Hence, we propose that sema4ab+ microglia..." would be appropriate.

The text "These data show that tgfb3 is a strong neurogenesis-promoting signal in fibroblasts and that it is controlled via sema4ab expression in microglia." should be revised to avoid confusing the reader that cell-autonomy was tested for sema4ab.

The authors state "we identify microglia-derived sema4ab as..." also implies cell-autonomy.

-Other minor comments:

Please provide a citation for "we found that MM#1 and MM#2 expressed higher levels of microglial marker genes" indicating how microglial marker genes were selected.

In figure 8C,D, and E, why is each transcript on a different graph?

# Author comment: Author response to Decision Letter 3

**DOI:** 10.1371/journal.pbio.3003865.r005

15 May 2026

Attachment Submitted filename: _Docampo_Seara_et_al_RevLetter_auresp_3.docx

# Editor report: Decision Letter 3

**DOI:** 10.1371/journal.pbio.3003865.r006

**Authors:** Lucas Smith, Lucas Smith, Lucas Smith, Lucas Smith

21 May 2026

Dear Dr Docampo Seara,

Thank you for your patience while we considered your revised manuscript "The microglia-derived protein sema4ab attenuates regenerative neurogenesis after spinal cord injury in zebrafish" for publication as a Research Article at PLOS Biology. This revised version of your manuscript has been evaluated by the PLOS Biology editors and the Academic Editor.

Looking through your revision and the new underlying data files that you have provided, we noted a few issues and inconsistencies that should be addressed before we can accept your study. I have detailed our concerns, below. While we have listed the specific examples that popped out at us, while we were looking at your revision, I would note that this may not be an exhaustive list. As this will likely be the last opportunity for to ensure the accuracy of your data I suggest that you take a careful look through the entire manuscript to ensure there are not other similar issues.

1) Looking at your response to our previous question about Figure 8, we realized that your Figure 8C-E does not actually show the data from your control animals (and I could not find this detail in the underlying data files either). I see you have indicated the average control value as a dotted line, set to one - but this gives no sense for the variance of that group, etc. We ask that you please update this figure to include the control data as its own bar. Please be sure to update your underlying data files, as well.

^related to this point, I see that Figures S4A, S5D, S6A, S9E, S10 have the same issue. Please look through your entire manuscript update these figures and underlying data files to include the control data as a separate bar as needed.

2) Please take a general look through the manuscript and figures to make sure readers can easily find the necessary information to fully assess the results of your study. As two examples:

-In the figures above please indicate in the figure legend what you used as a housekeeping gene for those experiments

-I noticed that Figure S10 has no details about what stats were used, etc. Please update this (and any other figure legend that needs it) to include those relevant details.

3) We noticed the sample size reported in Fig 8D vary from 3-5, however my understanding is that you are profiling animals from the same experimental condition in these graphs. Why do the n's differ between genes, in this panel?

4) We noticed that for the qPCR studies (including those in figure 8) that you are using a 1 sample t-test. We were wondering if a unpaired 2 sample t-test is more appropriate (as the control and mutant fish are separate biological groups...). If you agree, please repeat the analyses with the appropriate test, or please explain why a 1 sample t-test is appropriate in this context.

5) When skimming through the new underlying data that you provide in your S2_data file, I noticed that there are some discrepancies between the underlying data and the values reported in your figure. For example, fig 8A reports n=9/group but the underlying data file includes n=8 for gControl and n=10 for gSema4ab. I also noticed that the average values for these groups does not match between the data in S2_data and Fig 8A. Please take a close look through all of the data in your figures and in the underlying data, to ensure they are accurate and consistent.

6) In figure 8,F, I noticed that you report that tgfb3 is not expressed in mfap4 cells (with a value of 0)... however, by eye, it seems that there is some overlap between the mfap4 cells and the tgfb3 puncta in the representative images (ex bottom and leftmost orange arrow?). Please clarify whether there is any overlap between these signals and justify the quantification of 0 for those cells.

7) Apologies for not catching this earlier, but as part of our data policy, we require that you provide FCS files and a picture showing the successive plots and gates that were applied to the FCS files, for all FACS data generated in your study. As these files are usually quite big, you can deposit them in the Flow Repository (http://flowrepository.org/) or another data repository (like zenodo). Please make sure they are publicly available.

8) Thank you for providing the uncropped gel images related to your study as S1_raw_images file. Please update this file to annotate the gels with relevant information, as described here: https://journals.plos.org/plosbiology/s/figures#loc-blot-and-gel-reporting-requirements

ex. a complete file will include the following details:

Authors should annotate the gel image to clearly indicate the loading order, identity of experimental samples, method used to capture the image, and to specify which figure panel was generated from that original image. Molecular weight markers should be included or indicated on the raw image, and any lanes not included in the final figure should be marked with an “X” above the lane label on the original blot/gel image. All labeling and annotation should be performed without obscuring any data or background bands

**IMPORTANT: Along with your next revision, please be sure to include a point by point response for these requests and a track changes version of your paper. Please clearly detail, in your response, all changes made to the data in your figures and/or underlying data files. If you update the data or statistics, please explain exactly what was changed, and if/how this has altered the results of that comparison (including the p value). Please include an explanation for any changes. While I have primarily discussed the issues that I noticed with figure 8, as mentioned, I strongly encourage you to look through all of the figures presented in your study. If you need to make corrections to other figures, or other parts of the underlying data file, please be sure to clearly detail these changes as well.

Once you have made these changes, we will aim to discuss your revision with the Academic Editor - although we reserve the right to contact the original reviewers, if needed.

In addition to these revisions, you may need to complete some formatting changes, which you will receive in a follow up email. A member of our team will be in touch with a set of requests shortly. If you do not receive a separate email within a few days, please assume that checks have been completed, and no additional changes are required.

We expect to receive your revised manuscript within two weeks.

To submit your revision, please go to https://www.editorialmanager.com/pbiology/ and log in as an Author. Click the link labelled 'Submissions Needing Revision' to find your submission record. As mentioned, your revised submission must include the following:

- a cover letter that should detail your responses to any editorial requests, if applicable, and whether changes have been made to the reference list

- a Response to Reviewers file that provides a detailed response to the reviewers' comments (if applicable, if not applicable please do not delete your existing 'Response to Reviewers' file.)

- a track-changes file indicating any changes that you have made to the manuscript.

NOTE: If Supporting Information files are included with your article, note that these are not copyedited and will be published as they are submitted. Please ensure that these files are legible and of high quality (at least 300 dpi) in an easily accessible file format. For this reason, please be aware that any references listed in an SI file will not be indexed. For more information, see our Supporting Information guidelines:

https://journals.plos.org/plosbiology/s/supporting-information

*Published Peer Review History*

Please note that you may have the opportunity to make the peer review history publicly available. The record will include editor decision letters (with reviews) and your responses to reviewer comments. If eligible, we will contact you to opt in or out. Please see here for more details:

https://plos.org/published-peer-review-history/

*Press*

Should you, your institution's press office or the journal office choose to press release your paper, please ensure you have opted out of Early Article Posting on the submission form. We ask that you notify us as soon as possible if you or your institution is planning to press release the article.

*Protocols deposition*

To enhance the reproducibility of your results, we recommend that if applicable you deposit your laboratory protocols in protocols.io, where a protocol can be assigned its own identifier (DOI) such that it can be cited independently in the future. Additionally, PLOS ONE offers an option for publishing peer-reviewed Lab Protocol articles, which describe protocols hosted on protocols.io. Read more information on sharing protocols at https://plos.org/protocols?utm_medium=editorial-email&utm_source=authorletters&utm_campaign=protocols

Please do not hesitate to contact me should you have any questions.

Sincerely,

Luke

Lucas Smith, Ph.D.

Senior Editor

lsmith@plos.org

PLOS Biology

# Author comment: Author response to Decision Letter 4

**DOI:** 10.1371/journal.pbio.3003865.r007

2 Jun 2026

Attachment Submitted filename: _Docampo_Seara_et_al_RevLetter_auresp_4.docx

# Editor report: Decision Letter 4

**DOI:** 10.1371/journal.pbio.3003865.r008

**Authors:** Lucas Smith, Lucas Smith, Lucas Smith, Lucas Smith, Lucas Smith

5 Jun 2026

Dear Dr Docampo Seara,

Thank you for the submission of your revised Research Article "The microglia-derived protein sema4ab attenuates regenerative neurogenesis after spinal cord injury in zebrafish" for publication in PLOS Biology and thank you for addressing our last editorial comments in this revision. On behalf of my colleagues and the Academic Editor, Cody J. Smith, I am pleased to say that we can in principle accept your manuscript for publication, provided you address any remaining formatting and reporting issues. These will be detailed in an email you should receive within 2-3 business days from our colleagues in the journal operations team; no action is required from you until then. Please note that we will not be able to formally accept your manuscript and schedule it for publication until you have completed any requested changes.

Please take a minute to log into Editorial Manager at http://www.editorialmanager.com/pbiology/, click the "Update My Information" link at the top of the page, and update your user information to ensure an efficient production process.

PRESS

We frequently collaborate with press offices. If your institution or institutions have a press office, please notify them about your upcoming paper at this point, to enable them to help maximise its impact. If the press office is planning to promote your findings, we would be grateful if they could coordinate with biologypress@plos.org. If you have previously opted in to the early version process, we ask that you notify us immediately of any press plans so that we may opt out on your behalf.

We also ask that you take this opportunity to read our Embargo Policy regarding the discussion, promotion and media coverage of work that is yet to be published by PLOS. As your manuscript is not yet published, it is bound by the conditions of our Embargo Policy. Please be aware that this policy is in place both to ensure that any press coverage of your article is fully substantiated and to provide a direct link between such coverage and the published work. For full details of our Embargo Policy, please visit http://www.plos.org/about/media-inquiries/embargo-policy/.

Thank you again for choosing PLOS Biology for publication and supporting Open Access publishing. We look forward to publishing your study.

Sincerely,

Luke

Lucas Smith, Ph.D.

Senior Editor

PLOS Biology

lsmith@plos.org
